# Macrophage-reprogramming calcium alginate microspheres enhance exosome-mediated antigen cross-presentation to boost embolization-immunotherapy in hepatocellular carcinoma

**DOI:** 10.1016/j.mtbio.2026.103459

**Published:** 2026-07-18

**Authors:** Xiaoju Guo, Shiji Fang, Liyun Zheng, Mengzhu Han, Yiming Ding, Jiale Chen, Pan Qin, Yeyu Zhang, Gaofeng Shu, Zhongwei Zhao, Xiaoqiang Li, Chenying Lu, Minjiang Chen, Jiansong Ji

**Affiliations:** a Zhejiang Key Laboratory of Imaging and Interventional Medicine, the Fifth Affiliated Hospital of Wenzhou Medical University, Lishui, 323000, China; b Department of Radiology, Lishui Central Hospital, the Fifth Affiliated Hospital of Wenzhou Medical University, Lishui, 323000, China; c Cancer center, Lishui Central Hospital, the Fifth Affiliated Hospital of Wenzhou Medical University, Lishui, 323000, China; d Suzhou Institute of Biomedical Engineering and Technology, Chinese Academy of Sciences, Suzhou, 215163, China

**Keywords:** TACE, Microspheres, Tumor-associated macrophages, Exosome-mediated antigen cross-presentation, Immunotherapy, Hepatocellular carcinoma

## Abstract

Transarterial chemoembolization (TACE) is a first-line therapeutic modality for hepatocellular carcinoma (HCC). Nevertheless, its therapeutic efficacy remains constrained by the hostile tumor microenvironment (TME), typified by acidity and an immunosuppressive milieu. Here, multifunctional microspheres (RC6CaAlgMS) were developed to neutralize the acidic TME and relieve immunosuppression. The uniform-sized calcium alginate microspheres were fabricated using microfluidic technology, incorporating pH-responsive CaCO_3_ nanocarriers to efficiently encapsulate R848 and C6-ceramide (C6). Their physicochemical properties were characterized, and the embolization efficiency was validated using decellularized liver and rabbit kidney models. Furthermore, their antitumor activities and mechanism were evaluated in both *in vitro* and *in vivo*. R848 and C6 were efficiently encapsulated into RC6CaAlgMS, where they acted synergistically to reprogram tumor-associated macrophages (TAMs) toward an M1-like phenotype and to enhance both exosome secretion and exosome-mediated antigen cross-presentation. RC6CaAlgMS produced uniform vascular embolization and efficiently occluded the renal arterial branches. *In vitro* studies demonstrated that RC6CaAlgMS synergized with DOX-based chemotherapy to suppress the growth of murine HCC by neutralizing acidic TME and remodeling the immune landscape. When combined with PD-L1 blockade therapy, DOX-loaded RC6CaAlgMS effectively inhibited both primary and distant tumors, eliciting an abscopal-like effect driven by enhanced antigen dissemination and T-cell priming. In an orthotopic rat TACE model, the combination of DOX-loaded RC6CaAlgMS with PD-L1 blockade achieved complete tumor eradication. Collectively, this study establishes a multifunctional microsphere platform that effectively remodels and overcomes the post-TACE immunosuppressive TME, offering a potent strategy for integrating embolization with immunotherapy in HCC.

## Graphical abstract

**Figure:**
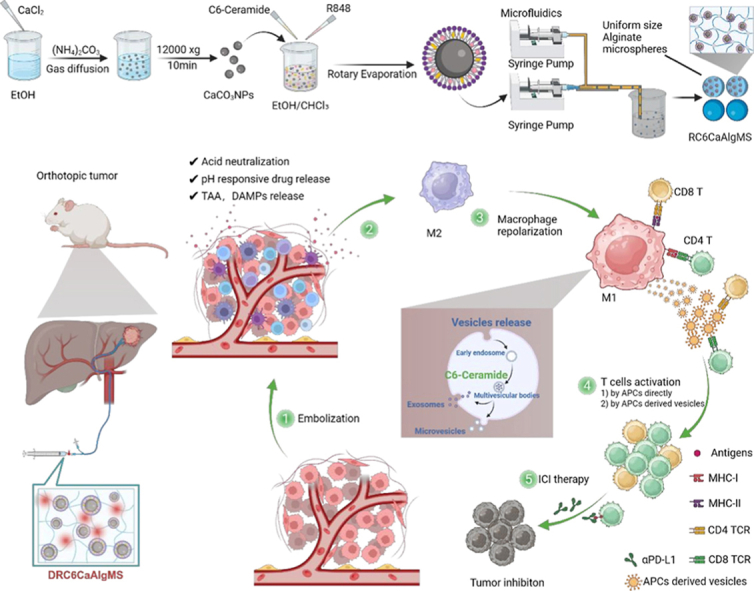

## 1 Introduction

Primary liver cancer remains a major global health burden, ranking as the sixth most frequently diagnosed malignancy and the third leading cause of cancer-related mortality worldwide [1,2]. Hepatocellular carcinoma (HCC), the predominant histological subtype, is frequently diagnosed at advanced stages, limiting curative treatment options. Transarterial chemoembolization (TACE) is the standard first-line therapy for intermediate-stage HCC [3]. Nonetheless, the objective response rate remains approximately 50% [4]. In recent years, immune-checkpoint inhibitors, including anti PD-1/PD-L1 and anti CTLA-4 antibodies, have achieved encouraging clinical outcomes in systemic treatment of HCC [5,6], prompting extensive efforts to integrate TACE with immunotherapy.

Mechanistically, TACE induces ischemic tumor necrosis and promotes the release of tumor-associated antigens, transiently generating an immunologically permissive microenvironment [7]. This has provided a strong rationale for TACE-based combination immunotherapy. Nevertheless, clinical benefits remain limited, particularly in advanced disease, largely due to the persistence of a profoundly immunosuppressive tumor microenvironment (TME), which constrains immune cell activation, trafficking, and effector function. Consequently, there is an urgent need to develop advanced embolic platforms capable of simultaneously delivering therapeutic agents and modulating the post-TACE immune milieu.

Accumulating evidence indicates that tumor progression is critically governed by the tumor microenvironment TME [8]. After TACE, the occlusion of supply arteries severely exacerbates hypoxia and lactate accumulation, inducing profound extracellular acidosis. Mechanistically, this lactic acid buildup directly drives immune dysfunction by inhibiting T-cell proliferation, cytokine production, and cytotoxicity [9,10]. Simultaneously, lactate downregulates dendritic cell functions to drive tolerogenic differentiation and polarizes tumor-associated macrophages (TAMs) toward the immunosuppressive M2 phenotype [11], establishing a pro-tumor, immunosuppressive state [12]. This acidic post-TACE microenvironment not only facilitates tumor survival, metastasis, and immune evasion [[13], [14], [15]], but also drives systemic immunosuppression [[16], [17], [18]]. To break this pathophysiological cascade, alleviating lactate accumulation and neutralizing tumor acidity have emerged as promising strategies to enhance embolic efficacy. Clinically, administering sodium bicarbonate to neutralize TME acidity markedly improves embolization outcomes [19,20]. Furthermore, we have demonstrated that CaCO_3_-based microspheres provide a prolonged neutralizing effect, achieving superior and sustained therapeutic outcomes [21,22].

In addition to metabolic dysregulation, TAMs constitute the dominant immune cell population within the HCC microenvironment and represent a major obstacle to effective immunotherapy [23]. TAMs are generally categorized into pro-inflammatory M1-like macrophages with antitumor activity and anti-inflammatory M2-like macrophages with pro-tumor activity. In advanced HCC, TAMs predominantly exhibit an M2 phenotype, characterized by impaired phagocytic capacity and the promotion of immune escape through multiple mechanisms [24]. Importantly, TACE further induces the expansion of TREM2^+^ macrophage subsets, which hinders the infiltration and function of CD8^+^ T cells, thereby correlating with adverse prognostic outcomes [25]. Accordingly, pharmacological reprogramming of M2-TAMs toward an M1 phenotype using Toll-like receptor agonists, such as R848, has emerged as an effective strategy to reverse immunosuppression and enhance antitumor immune responses [26].

As professional antigen-presenting cells within the TME, macrophages play a central role in orchestrating sustained T cell activation and antitumor immunity [27,28]. Crucially, TACE-induced ischemic infarction and tumor necrosis trigger a massive, transient wave of tumor-associated antigen (TAA) release, alongside damage-associated molecular patterns (DAMPs), into the local milieu. In an immunologically favorable environment, professional antigen-presenting cells would typically capture these shed TAAs and process them onto major histocompatibility complex (MHC) molecules to prime naive T cells [29]. However, multiple features of the TME, including physical barriers and immunosuppressive signaling, restrict these cellular interactions and impair functional cooperation. Recent studies indicate that extracellular vesicles, such as exosomes, function as non-contact communication vehicles, effectively traversing spatial barriers to deliver antigens and stimulatory signals [30,31]. Notably, exosomes originating from M2-like macrophages have been shown to promote stemness, proliferation, drug resistance, and metastasis in HCC cells. In contrast, exosomes derived from M1-like macrophages exhibit a robust capacity for antigen cross-presentation [32]. Consequently, correcting post-TACE acidic and reprogramming TAMs toward an M1 phenotype, while simultaneously stimulating exosome release, may greatly amplify antitumor immune responses [33]. In this context, ceramide analogues have been shown to induce multivesicular body formation and promote exosome biogenesis [34]. Among them, C6-ceramide (C6) is a bioactive sphingolipid that regulates cell proliferation, apoptosis, and immune signaling [35], and has emerged as an attractive molecular tool to enhance vesicle-mediated immune communication.

Here, we prepared calcium alginate microspheres co-loaded with R848 and C6 (RC6CaAlgMS) to enhance the therapeutic efficacy of TACE. With a uniform size distribution and pH-responsive behavior, RC6CaAlgMS achieved efficient vascular embolization while neutralizing the acidic TME following TACE. Through R848 induced macrophage immunomodulation and C6 driven vesicle biogenesis, RC6CaAlgMS reprogrammed TAMs and promoted exosome-mediated antigen cross-presentation. *In vitro*, macrophage polarization, phagocytosis assays, exosome characterization, and T cell co-culture systems were employed to delineate the mechanistic link between macrophage-reprogramming, exosome release, and CD8^+^ T cell activation. *In vivo,* murine and orthotopic rat HCC models demonstrated that doxorubicin-loaded RC6CaAlgMS remodel the TME and elicit robust local and systemic antitumor immunity. Collectively, this work establishes a multifunctional embolic platform that couple tumor acid neutralization with immune reprogramming, offering a promising strategy to improve embolization-immunotherapy for HCC treatment.

## 2 Results and discussion

### 2.1 Macrophages correlate with TACE efficacy in HCC

**Fig. 1:**
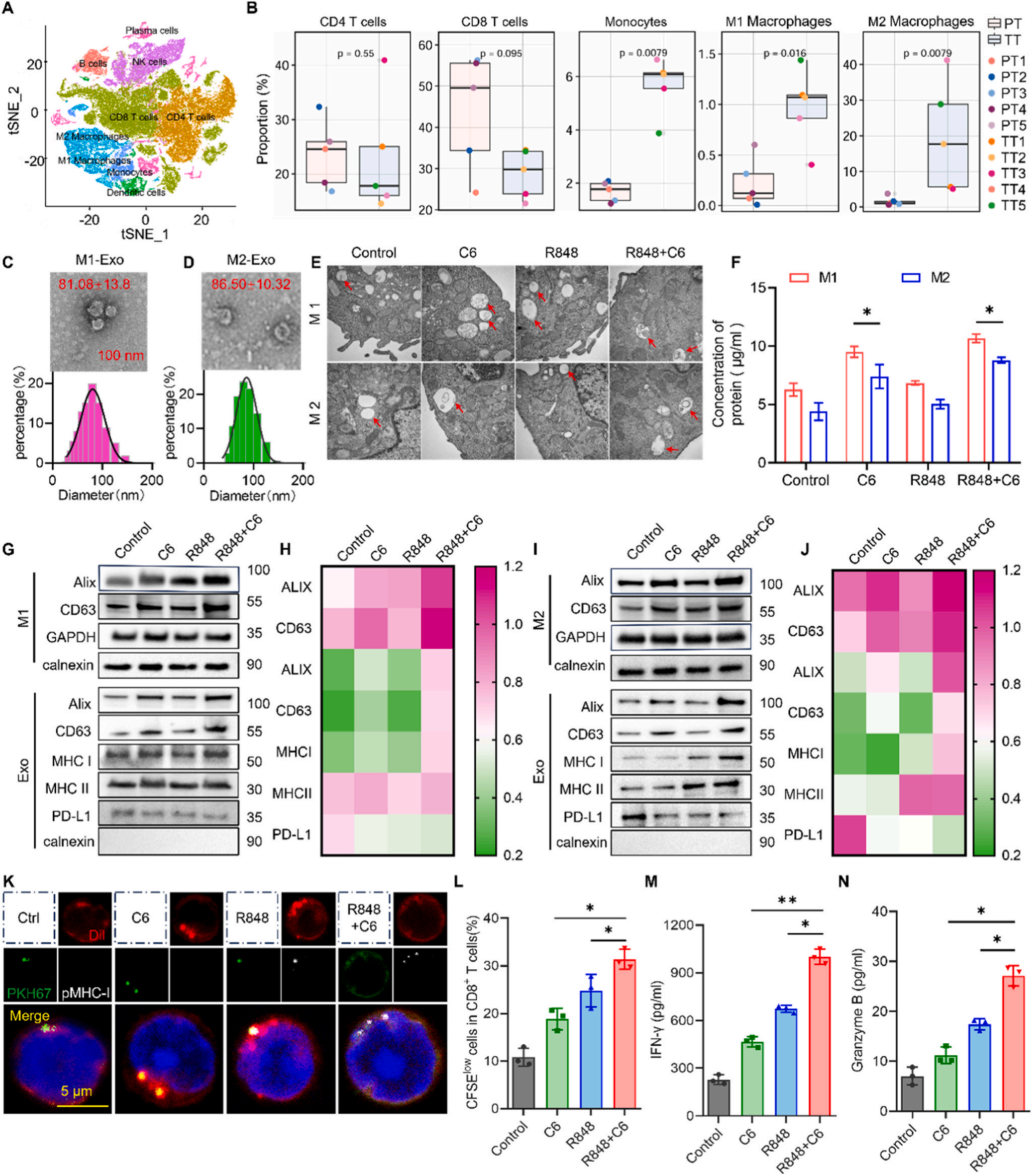
C6 and R848 synergistically promoted macrophage reprogramming and immune function. (A) The t-SNE plot displayed the annotation of cells in the nine clusters. (B) Box plots showing the relative proportions of immune cell clusters across the PT and TT groups in the discovery cohort (n = 5 per group). PT, primary tumor; TT, TACE tumor. Representative TEM images and size distribution of exosomes isolated from (C) M2-like macrophages and (D) M1-like macrophages. Scale bar: 100 nm. (E) Representative TEM images showing the formation of MVBs and ILVs in M1-like macrophages and M2-like macrophages under different treatment conditions (n = 3, R848 = 1 μg/mL, C6 = 4 μg/mL) (F) Protein concentration of exosomes derived from M1-and M2-like macrophages. (G) Western blot analysis of CD63 and Alix expression in M1-and (I) M2-like macrophages lysates and exosomes. MHC II, MHC I, and PD-L1 expression were also examined in exosomal fractions. (H, J) The statistical heatmap showing the relative expression levels of the indicated proteins in M1-and M2-like macrophages and their exosomes. (K) Representative CSLM images showing the co-localization of PKH67-labeled exosomes (green) with Alexa Fluor 594-labeled pMHCI (white) and Dil-stained CD8^+^ T cells membrane (red) after a 2-h co-culture. Cell nuclei were counterstained with DAPI (blue). Scale bar: 5 μm. (L) Semi-quantitative analysis of CFSE^low^ CD8^+^ T cells following co-culture with the indicated exosomes. (M) Concentration of IFN-γ and (N) Granzyme B in the co-culture supernatant measured by ELISA. Data are presented as mean ± SD. (n = 3 biologically independent samples). The statistical analysis was performed with ANOVA. P > 0.05 indicated no significance between two groups. *P < 0.05, **P < 0.01.

To delineate the immune landscape shaped by TACE in HCC, we analyzed publicly available single-cell RNA-sequencing (scRNA-seq) from PRJNA793914. t-SNE clustering identified nine major immune populations, including B cells, CD4^+^ and CD8^+^ T cells, NK cells, DCs, monocytes, M1-like and M2-like macrophages, and plasma cells (Fig. 1A). Compared to primary tumors, the TACE-treated tumors exhibited an increased overall number of myeloid cells, including monocytes and macrophages, was increased, whereas a significant reduction was observed in the infiltration of natural killer (NK) cells and CD8^+^ T cells (Fig. 1B and S1). Further investigation identified a substantial increase in the proportion of M2-like macrophages (p = 0.0079), which emerged as the dominant subset and accounted for nearly 20% of total immune infiltrates. These M2-TAMs exhibited a canonical immunosuppressive signature characterized by upregulated PD-L1 expression, enhanced production of inhibitory cytokines, and impaired antigen-presenting capacity, collectively supporting diminished T-cell priming, functional hypo-responsiveness, and progressive T-cell exhaustion [[36], [37], [38]]. Together, these findings suggest that TACE, despite its cytoreductive intent, may unintentionally drive the TME toward an immunosuppressive, M2 polarized state that restricts effective antitumor immunity.

Furthermore, we analyzed the GSE104580 RNA-seq dataset to compare the transcriptional profiles of TACE responders and non-responders. Immune cell infiltration analysis using CIBERSORT revealed distinct patterns between these two groups, marked by significant differences in the infiltration of macrophages, including M0, M1, and M2 subtypes (Fig. S2). These results indicate that alterations in macrophages polarization states are associated with the response to TACE. Collectively, our findings establish a link between macrophages polarization status and TACE efficacy, suggesting that reprogramming macrophages phenotypes post-TACE could represent a promising therapeutic strategy to enhance treatment outcomes, particularly when combined with immunotherapy.

### 2.2 C6 and R848 synergistically enhance the antigen-presenting capacity of reprogrammed macrophages via exosome release

Within TME, M2-like macrophages represent the predominant phenotype with immunosuppressive properties and hinder the efficacy of TACE-induced immune responses [39]. Whereas M2-TAMs exhibit diminished antigen-presenting capacity, M1-polarized macrophages possess stronger pro-inflammatory potential but remain limited in mediating long-range antigen dissemination [33]. To overcome these spatial and functional limitations, exosome-mediated antigen presentation has emerged as a promising strategy to potentiate antitumor immune responses. We hypothesize that a combined approach, which reprograms M2-TAMs towards an M1 phenotype while concurrently stimulating the release of antigen-laden exosomes from M1-like macrophages, would create a potent and synergistic stimulus to ignite a robust and systemic antitumor immune response.

To test this hypothesis, we used R848 to reprogram immunosuppressive M2-like macrophages, in combination with C6, which robustly augments the secretion of immunomodulatory exosomes from macrophages [40]. We first assessed the cytotoxicity of two agents in Hepa1-6 cells, M1-and M2-like macrophages. R848 alone did not cause cell viability decreased in any of these three cell types, even at high concentration to 64 μg/mL (Fig. S3). Notably, C6 exhibited no detectable toxicity toward macrophages at concentrations up to 25.8 μg/mL, yet induced marked dose-dependent cytotoxicity in Hepa1-6 tumor cells with an IC_50_ of 11.88 μg/mL (Fig. S4), indicating its preferential antitumor activity while preserving immune cell viability. To investigate macrophage reprogramming, bone marrow-derived macrophages (BMDMs) were educated into TAM-like phenotypes using Hepa1-6 conditioned medium and subsequently treated with vehicle control, R848, C6, or both agents for 24 h. Flow cytometry (FCM) analysis showed that R848 significantly increased the proportion of F4/80^+^CD86^+^ M1-like macrophages to 39.0 ± 4.3%, while the combination treatment further elevated this population to 53.0 ± 4.7%. In parallel, R848 reduced F4/80^+^CD206^+^ M2-like macrophages to 33.2 ± 1.4%, with the C6 and R848 combination further decreasing the fraction to 22.6 ± 3.3%. Although the difference was not significant, C6 alone did slightly increase M1-like macrophages and slightly decrease the percentage of M2-like macrophages (Fig. S5). This result is consistent with previous reports indicating that C6 can redirect TAMs towards an antitumor M1 phenotype by suppressing intracellular ROS levels [35]. Moreover, ELISA assays demonstrated that treatment with R848+C6 treatment markedly enhanced the secretion of IL-12 and TNF-α in macrophages supernatants compared with either agent alone (Fig. S6). These results indicate that R848 effectively triggers the polarization of macrophages toward M1 phenotype, and that C6 does not diminish the efficacy of R848. Secondly, we investigated the effect of the C6 and R848 combination on exosomes release from macrophages. Exosomes isolated from M1-and M2-polarized BMDMs were characterized by dynamic light scattering (DLS), which showed mean particle diameters of 81.08 ± 13.8 nm and 86.50 ± 10.32 nm, respectively. Transmission electron microscopy (TEM) further verified the canonical cup-shaped morphology of isolated vesicles, with sizes consistent with DLS measurements (Fig. 1C and D). Notably, intraluminal vesicles (ILVs) serve as the intracellular precursors of exosomes, which are released extracellularly following multivesicular body (MVB) fusion with the plasma membrane [41]. TEM revealed that, compared with vehicle control, C6 significantly increased the number of MVBs and ILVs in M1-like macrophages (Fig. 1E). These results demonstrated that C6 treatment enhanced the release of exosomes from M1-like macrophages. A similar increase was observed when C6 was combined with R848, demonstrating that R848 does not compromise the exosome-promoting activity of C6. To determine whether these effects extend to M2-like macrophages, we next assessed exosome biogenesis and secretion in this population. As shown in Fig. 1E and F, with or without R848, increased intracellular ILV/MVB formation and elevated the abundance of exosome proteins in the culture supernatant, as evidenced by a significant increase in exosome protein levels in the conditioned medium. Notably, the combination of C6 and R848 yielded a further enhancement compared with C6 alone. This effect is primarily attributable to the reprogramming of M2-like macrophages toward an M1-like phenotype, consistent with the higher exosome output observed in C6-treated M1-like macrophages relative to M2-like macrophages. Together, these results demonstrate that C6 promotes exosome release from macrophages, and that its combination with R848 further amplifies this effect primarily through reprogramming M2-like macrophages toward an M1-like phenotype.

Exosomes released by M1-and M2-like macrophages are known to display distinct immunological signatures. M1-derived exosomes generally reinforce antitumor immunity, whereas those from M2-like macrophages promote immunosuppression and tumor progression [42,43]. We therefore examined whether C6 in combination with R848 could reprogram M2-derived exosomes toward an immunostimulatory phenotype. Western blot analysis revealed that C6, but not R848 alone, led to increased expression of canonical exosome markers CD63 and ALIX, validating the enhanced exosomes release from M1-like macrophages. Consistently, the combination of C6 and R848 treatment increased the expression of CD63 and ALIX. Importantly, C6, either alone or in combination with R848, increased MHC-I and MHC-II while reducing PD-L1 in M1-derived exosomes, demonstrating their potential to enhance antigen presentation while alleviating immunosuppression (Fig. 1G and H). In M2-like macrophages, C6 similarly increased CD63 and ALIX and suppressed PD-L1, but did not alter MHC-I or MHC-II. This selective protein pattern likely reflects partial C6-induced repolarization toward an M1-like state, thereby modifying the functional properties of their secreted exosomes. Importantly, the combination of C6 and R848 induced robust upregulation of MHC-I and MHC-II with concurrent PD-L1 downregulation in M2-derived exosomes, demonstrating effective reprogramming of their immunological phenotype (Fig. 1I and J).

Productive macrophage-T cell collaboration in antitumor defense entails multiple distinct signaling modalities, including direct immunological synapse formation as well as indirect communication via extracellular vesicles. To assess the effect of exosome-mediated antigen presentation, we analyzed co-cultured T cells by confocal laser scanning microscope (CSLM). Exosomes isolated from the conditioned media were labeled PKH67 then co-incubated with Dil-labeled CD8^+^ T cells for 2h. Compared to the control, exosomes derived from C6+R848-treated macrophages showed enhanced binding to CD8^+^ T cells and were enriched in pMHC-I complexes (Fig. 1K). Furthermore, unlabeled exosomes were co-incubated with CFSE-labeled CD8^+^ T cells for five days to assess T-cell proliferation by FCM. Exosomes derived from macrophages treated with the C6+R848 combination markedly enhanced CD8^+^ T-cell expansion, with the proportion of proliferating cells reaching 30.0 ± 2.6% (Fig. 1L), which is substantially higher than that induced by exosomes from macrophages treated with either agent alone. In parallel, IFN-γ and granzyme B levels were quantified in the culture supernatant after 24 h of co-culture. CD8^+^ T cells stimulated with exosomes from the C6+R848 group produced significantly higher amounts of both cytokines compared to the single-agent groups (Fig. 1M and N). Together, these findings demonstrate that C6 and R848 reprogram macrophages-derived exosomes to enhance CD8^+^ T cell responses. Together, these findings demonstrate that C6 and R848 cooperatively reprogram macrophage-derived exosomes toward an immunostimulatory phenotype that enhances CD8 T-cell proliferation and effector function.

To further investigate how these treatments altered the proteomic profile of macrophages-derived exosomes, BMDMs were polarized into M2 phenotype using M-CSF and appropriate cytokines. After replacing with serum-free medium, M2 macrophages were treated with C6 and R848 or vehicle control for 24 h, and exosomes were subsequently isolated for proteomic profiling. Quantitative proteomics identified 18,682 exosomal proteins across conditions. Differentially expressed proteins (DEPs) were determined based on a fold change threshold (>1.5 or < −1.5) with a significance cut-off of p < 0.05, revealing 189 DEPs relative to vehicle control, of which 24 were upregulated and 136 downregulated in the C6+R848 group (Fig. S7A). To elucidate biological processes associated with these alterations, we performed Gene Ontology (GO) and pathway enrichment analyses on DEPs from M2-derived exosomes after C6+R848 exposure. As shown in Fig. S7B, GO analysis identified the biological processes affected by C6 and R848 treatment in the M2 phenotype. The processes mainly included immune response-activating signaling pathway, positive regulation of T cell migration, antigen receptor-mediated signaling pathway, antigen processing and presentation of peptide antigen via MHC I and vesicle-mediated transport. The results revealed significant enrichment in pathways related to immune regulation, vesicle transport, and antigen presentation, suggesting that exosomal proteins may facilitate antigen presentation and immune activation. Furthermore, gene set enrichment analysis (GSEA) showed that the enhanced immune function of C6 and R848 treatment were related to exosome development and antigen presentation capacity of macrophages (Fig. S7C). These results suggest that C6 and R848 treatment causes changes of genes related to modulation of immune activation. Together, these findings indicate that exosomal proteins are not only engaged in structural and transport functions but also actively contribute to immune regulation and inflammatory responses, thereby shaping the TME.

### 2.3 Preparation of R848 and C6 co-loaded CaCO_3_ microspheres

**Fig. 2:**
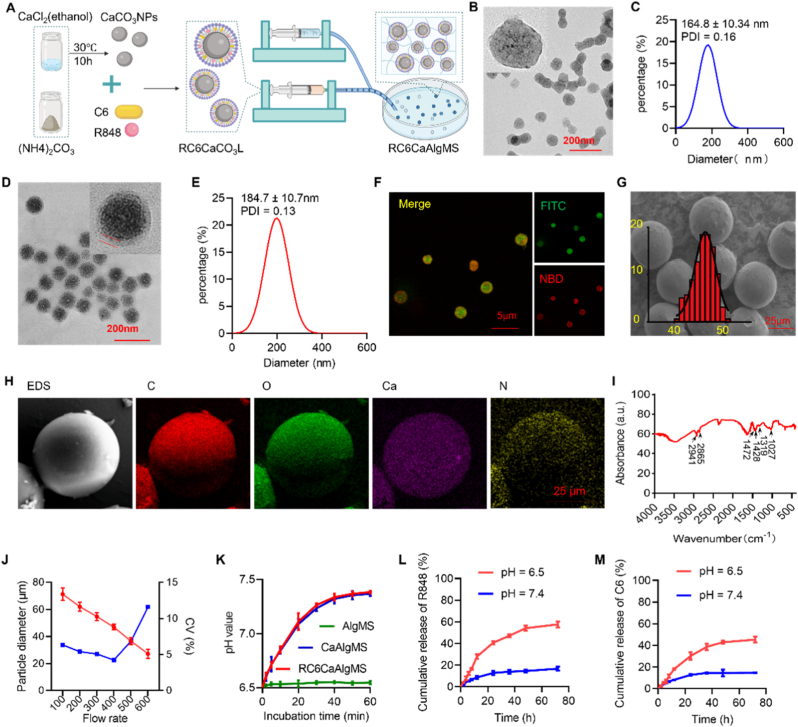
Preparation and characterization of RC6CaAlgMS. (A) Schematic illustration of the stepwise synthesis procedure of RC6CaAlgMS. (B) TEM images and (C) corresponding size distribution histogram of CaCO_3_ nanoparticles. scale bar: 200 nm. (D) TEM images and (E) corresponding size distribution histogram of the CaCO_3_L. Scale bar: 200 nm. (F) CLSM images of RC6CaLNPs. Note that the green signal (FITC) corresponds to the CaCO_3_ core, while the red signal (NBD-C6) represents the loaded drug. Scale bar: 5 μm. (G) SEM images and corresponding size distribution histogram of the RC6CaAlgMS microspheres. Scale bar: 25 μm. (H) EDS elemental mapping of RC6CaAlgMS, showing the uniform distribution of C, O, Ca, and N elements, which confirms the successful incorporation of various components. (I) FTIR spectroscopy analysis of RC6CaAlgMS and its individual components. (J) The influence of the continuous phase flow rate (100-600 μL/min) on the size and CV of RC6CaAlgMS was systematically investigated. Note: the dispersed phase flow rate was fixed at 10 μL/min and a 32G syringe needle was used. (K) The pH-responsive behavior of different microspheres. 50 mg of RC6CaAlgMS, CaAlgMS, and AlgMS were separately incubated in PBS (pH 6.5). The change in pH of the PBS was recorded over time. *In vitro* cumulative release of (L) R848 and (M) C6 from 10 mg RC6CaAlgMS in PBS at pH 7.4 and 6.5. Data are presented as mean ± SD (n = 3).

CaCO_3_ microspheres were engineered to co-load R848 and C6, serving as embolic agents for TACE. Briefly, the CaCO_3_ nanoparticles were synthesized by a vapor-diffusion method and further assembled into CaCO_3_-liposome hybrid nanoparticles (RC6CaL) via a solvent-diffusion approach. Then, the microspheres were synthesized using a simplified microfluidic device, in which paraffin containing Span-80 and CaI_2_ as the continuous phase and an aqueous solution of alginate together with the drug-loaded hybrid CaCO_3_ nanoparticles as the dispersed phase (Fig. 2A). The obtained microsphere was named as RC6CaAlgMS. DLS analysis revealed that the obtained CaCO_3_ nanoparticles had an average diameter of 164.8 ± 10.34 nm (Fig. 2C). After hybrid formation, the average diameter slightly increased to 184.7 ± 10.7 nm (Fig. 2E), and a distinct membranous structure was observed under TEM, confirming successful preparation of the hybrid nanoparticles (Fig. 2B and D). TEM observations revealed that the size of CaCO_3_ nanoparticles were consistent with DLS analysis. CLSM images confirmed the stronger colocalization (yellow) of C6 NBD Ceramide(red) with FITC-labeled CaCO_3_ (green), validating successful C6 loading into the liposomal membrane (Fig. 2F). Quantification of the encapsulation efficiency (EE) upon degradation of the CaCO_3_ lipid scaffold revealed values of 75.12 ± 9.02% for C6 and 85.00 ± 5.63% for R848 (Fig. S8).

Using the microfluidic method, monodisperse microspheres were obtained, with particle size precisely controlled by adjusting the flow rates of the dispersed and continuous phases (Fig. S9), making them suitable for diverse applications. In this study, the microspheres prepared at the continuous phase of 400 μL/min were selected. The microspheres with an average diameter of 47.02 μm with a coefficient of variation (CV) of 4.23, exhibiting a swollen diameter of about 75.27 μm (Fig. 2J and S9). Scanning electron microscopy (SEM) showed that RC6CaAlgMS exhibited a regular spherical morphology (Fig. 2G). Energy-dispersive X-ray spectroscopy (EDS) indicated that the homogeneous distribution behaviors of Ca, C, O, and N signals, with their spatial locations highly overlapping (Fig. 2H). This homogeneous distribution provides compelling evidence for the successful formation of a uniformly integrated nanocomposite, rather than a simple physical mixture of separate components. Specifically, the co-localization of Ca signals suggests the preservation of the CaCO3-based structure. Concurrently, the uniform signals of N strongly indicate the successful and even incorporation of nitrogen-containing organic components into the microsphere matrix. Fourier-transform infrared (FTIR) (Fig. 1I) revealed that the characteristic absorption bands of polysaccharides are typically observed at approximately 1027 cm^−1^ and 1319 cm^−1^, and the splitting peaks at around 1428 cm^−1^ and 1472 cm^−1^ were assigned to the asymmetric stretch in carbonate, indicating the existence of CaCO_3_. Moreover, the characteristics of methylene vibration bands at 2941 cm^−1^ and 2865 cm^−1^ in phospholipid or C6 were also observed in RC6CaAlgMS. These results suggested that RC6CaAlgMS had been successfully prepared. To evaluate the acid-neutralizing capacity of the CaCO_3_-based microspheres, we first monitored the pH variation in PBS under acidic conditions mimicking the TME. As shown in Fig. 2K, the pH value of the PBS solution (initial pH = 6.5) increased rapidly upon the addition of CaAlgMS and RC6CaAlgMS, reaching approximately 7.0 within 10 min and stabilizing at a near-neutral level (pH ∼7.2) after 30 min. In contrast, the pH of the AlgMS group remained unchanged. These results collectively demonstrate the potent and responsive acid-neutralizing capability of our RC6CaAlgMS, which is crucial for alleviating tumor acidity. Inspired by the acid-triggered degradation of CaCO_3_, the pH-responsive release of C6 and R848 from RC6CaAlgMS was investigated. As expected, both C6 and R848 were released more rapidly under acidic conditions than at physiological pH, consistent with the degradation behavior of CaCO_3_ (Fig. 2L and M).

### 2.4 RC6CaAlgMS reprograms macrophage polarization and modulates exosome function to enhance immune activation

**Fig. 3:**
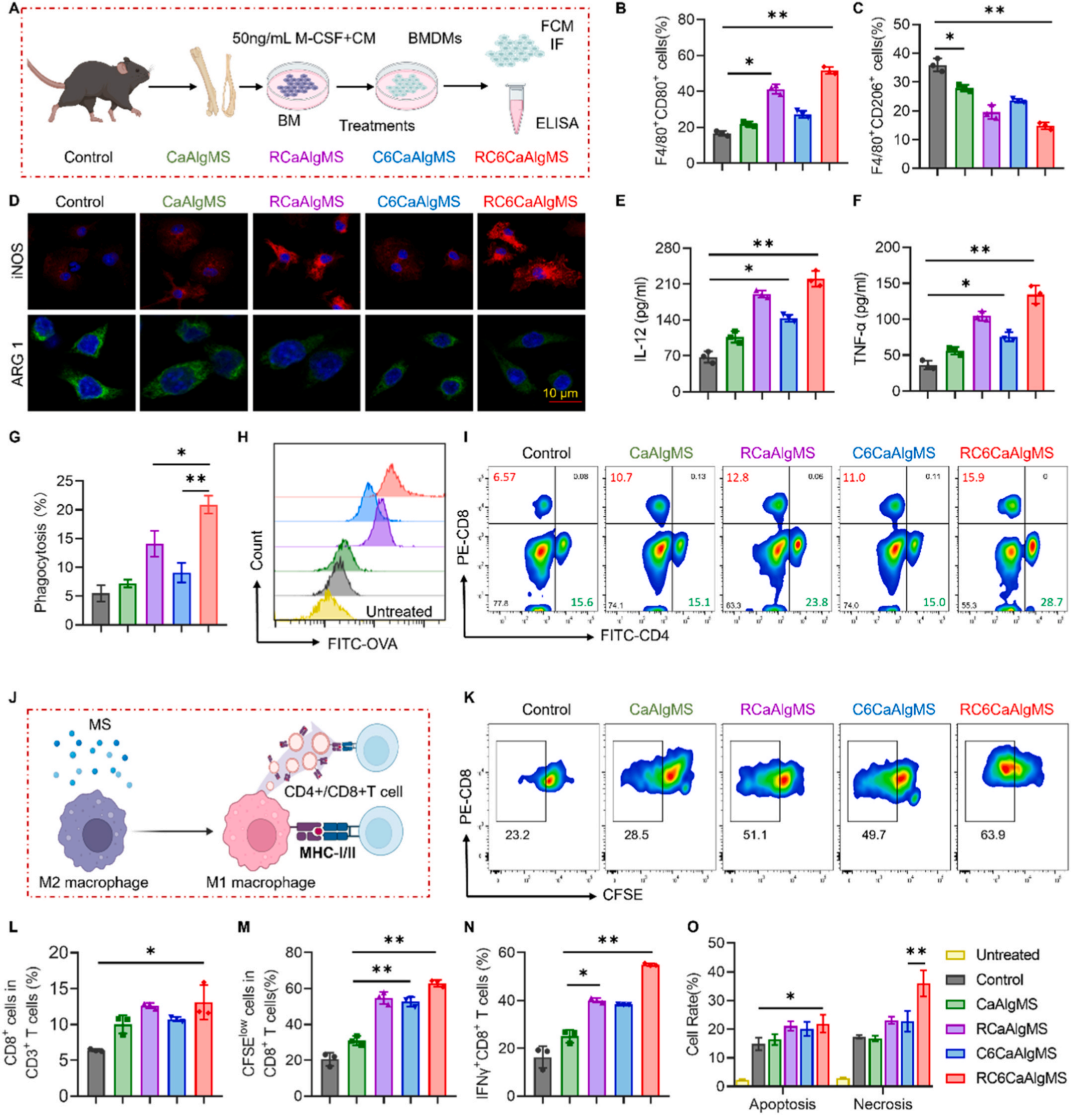
Modulation of macrophages polarization and immune function by different treatments (A) Schematic diagram illustrating the experimental design for treating TAMs with different agents *in vitro*. Semi-quantitative analysis of (B) M1 (F4/80^+^CD80^+^) and (C) M2 (F4/80^+^CD206^+^) polarization markers in TAMs after various treatments. (D) Immunofluorescence staining images showing the expression of iNOS (M1 marker) and ARG1 (M2 marker) in differently treated TAMs. Scale bar: 10 μm. ELISA quantification of the pro-inflammatory cytokines (E) IL-12 and (F) TNF-α secreted into the supernatant by the polarized TAMs. Data are mean ± SD (n = 3). (G) Semi-quantitative analysis of phagocytosis rate in various groups. (H) Representative FCM histograms showing the fluorescence intensity of FITC-OVA in TAMs under different treatment conditions. (I) Representative FCM plots of CD3^+^CD4^+^ CD8^−^ and CD3^+^CD4^−^CD8^+^ T cell populations after co-culture with 20 μg/mL exosomes. (J) Schematic illustration of the proposed mechanism by which RC6CaAlgMS reprograms macrophages to activate CD8^+^ T cell immunity. (K) Representative FCM plots and (M) semi-quantitative analysis of CFSE-labeled CD8^+^ T cells. (L) Semi-quantitative analysis of CD3^+^CD4^+^T cells and (N) CD3^+^CD8^+^IFN-γ^+^T cells after co-culture with 20 μg/mL exosomes. (O) Semi-quantitative analysis of tumor cell apoptosis when T cells were co-cultured with tumor cells in the presence of antigen and exosomes derived from differently treated macrophages. The statistical analysis was performed with ANOVA. P > 0.05 indicated no significance between two groups. *P < 0.05, **P < 0.01.

Thereafter, the capacity of RC6CaAlgMS to reprogram TAMs toward an M1 phenotype was evaluated. Briefly, BMDMs were conditioned with a 40% (v/v) mixture of tumor-derived supernatant for 24 h to generate TAMs. These TAMs were then treated with PBS, CaAlgMS, RCaAlgMS, C6CaAlgMS, or RC6CaAlgMS for 24h (Fig. 3A). FCM analysis (Fig. 3B and C and S10) showed that RC6CaAlgMS markedly increased the proportion of M1-like macrophages to 51.53 ± 1.96%, which was 3.10-fold higher than that in cells treated with PBS. Although RCaAlgMS treatment increased the proportion of M1-like macrophages, the percentage remained much lower than that induced by RC6CaAlgMS. In contrast, CaAlgMS and C6CaAlgMS slightly reduced M2 polarization. Immunofluorescence staining further revealed increased iNOS expression and reduced ARG1 expression in macrophages treated with RC6CaAlgMS (Fig. 3D). Consistently, ELISA assays indicated a significant upregulation of IL-12 and TNF-α secretion in the supernatants of RC6CaAlgMS-treated cells (Fig. 3E and F), indicating the induction of a pro-inflammatory phenotype. Together, these results demonstrate that incorporation of R848 endows RC6CaAlgMS with the capacity to drive robust M1-like phenotype.

Beyond macrophages polarization, R848 has been reported to enhance macrophages-mediated tumor cell phagocytosis [44]. We next sought to determine whether RC6CaAlgMS enhanced phagocytic activity using a co-culture system of TAMs and Hepa1-6 cells under hypoxic conditions, mimicking the hypoxic and ischemic TME following TACE *in vitro*. CLSM images indicated that a large number of Hepa1-6 cells were phagocytosed by TAMs, as evidenced by the colocalization of CFSE-labeled Hepa1-6 cells with red fluorescent tracker-labeled TAMs (Fig. S11). FCM analysis indicated that the percentage of double-positive cells (red fluorescent tracker^+^/CFSE^+^) reached 20.90 ± 1.54% in the co-culture system treated with RC6CaAlgMS, which was higher than that in any other groups (Fig. 3G). Moreover, upon incubation with RC6CaAlgMS, TAMs showed higher antigen-processing capability, as indicated by a stronger FITC-OVA fluorescence signal compared with other groups (Fig. 3H and S12). These results indicate that RC6CaAlgMS enhances both the phagocytic activity and antigen-processing capacity of macrophages.

As an antigen-presentation cell, macrophages effectively enhanced the activation of T cells through direct interactions or via exosome-mediated mechanisms [32] (Fig. 3J). We isolated exosomes from the supernatant of TAMs treated with PBS, CaAlgMS, RCaAlgMS, C6CaAlgMS, or RC6CaAlgMS. These exosomes were then co-cultured with naive CD3^+^ T cells for 24 h. FCM analysis revealed a significant expansion of T cell populations upon exposure to exosomes from the RC6CaAlgMS group. The proportion of CD4^+^ T cells increased to 30.53 ± 1.90%, and CD8^+^ T cells rose to 13.10 ± 2.43%, values that were substantially higher than those in the PBS and AlgMS control groups (Fig. 3I–L and S13A). CFSE dilution assays demonstrated that these exosomes robustly promoted the proliferation of CD8^+^ T cells, as evidenced by a significant increase in the proportion of CFSE^low^ cells (Fig. 3K and M). Beyond population expansion of CD8^+^ T cells, we assessed functional activation by measuring intracellular IFN-γ expression. A markedly higher percentage of IFN-γ^+^ cells was detected within the CD8^+^ T cell population co-cultured with exosomes from RC6CaAlgMS-treated TAMs (Fig. 3N and S13B). Furthermore, to directly evaluate the cytotoxic capacity of these activated T cells, we performed a co-culture killing assay against Hepa1-6 tumor cells. CD8^+^ T cells that had been primed with exosomes from the RC6CaAlgMS group exhibited the most potent tumoricidal activity, leading to a dramatic reduction in viable tumor cells compared to all other groups (Fig. 3O–S14). Taken together, these results demonstrate that RC6CaAlgMS effectively reprograms M2-TAMs into pro-inflammatory M1-like macrophages and activates CD4^+^ T cells and CD8^+^ T cells to selectively eliminate tumor cells, at least in part through an exosome-mediated mechanism (Fig. 3J).

### 2.5 Pharmacokinetic and neutralization evaluation of RC6CaAlgMS

**Fig. 4:**
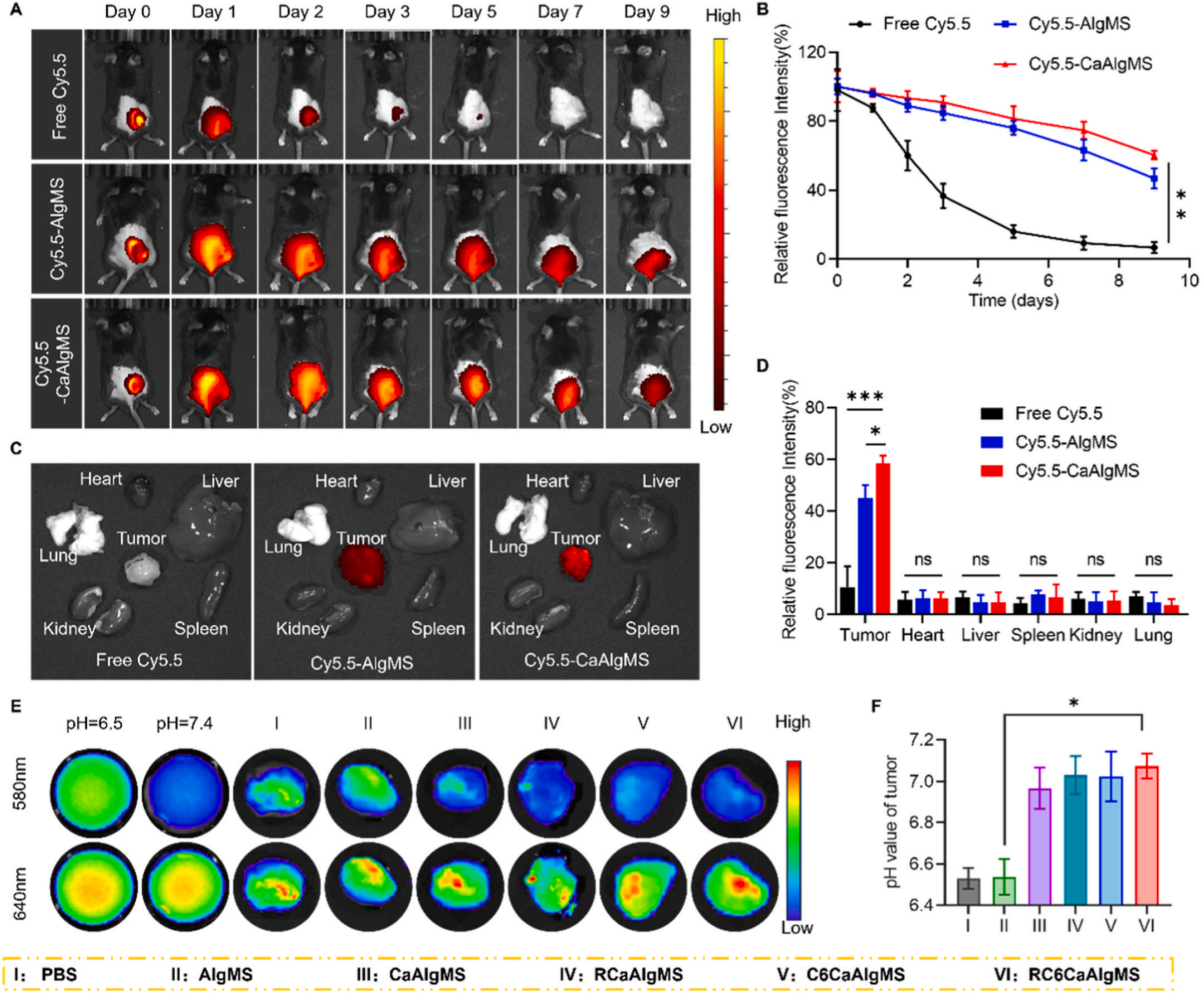
*In vivo* biodistribution, sustained release, and TME modulation of Cy5.5-CaAlgMS. (A) Evaluating the *in vivo* release behavior of 10 mg Cy5.5-CaAlgMS using the IVIS Spectrum imaging system. (B) Relative fluorescence in the tumor region from day 0 to day 9 after peritumoral injection of Cy5.5-CaAlgMS (n = 3). (C) Biodistribution of Cy5.5 fluorescence and relative fluorescence (D) in major organs and tumors. (E) Representative fluorescence images of tumor tissues harvested 3 day post various treatments. The mice were intravenously injected with a pH-sensitive dye (SNARF-4F) 20 min prior to sacrifice. (F) The pH values of tumor tissues harvested 3day post various treatments. The pH is measured as the ratio of two peaks in the SNARF emission at 580 nm and 640 nm, converting the ratio into pH using a calibration curve. The statistical analysis was performed with ANOVA. P > 0.05 indicated no significance between two groups. *P < 0.05, **P < 0.01,***P < 0.001.

Considering the pH-responsive drug release and biodegradability of CaCO_3_-AlgMS microspheres [45], we next evaluated the pharmacokinetics of the obtained microspheres using a Hepa1-6 tumor-bearing mice model. Mice with tumor volumes of approximately 200 mm^3^ were randomized into three groups and received peritumoral injections of free Cy5.5, Cy5.5-AlgMS, and Cy5.5-CaAlgMS, respectively. Fluorescence intensity in the tumor region was monitored at 0, 1, 2, 3, 5, 7and 9 days post-injection (Fig. 4A and B). Tumors treated with Cy5.5-AlgMS and Cy5.5-CaAlgMS exhibited a more sustained fluorescence signal with a relatively slower decline than that of free Cy5.5. The fluorescence signal persisted for over 9 days in the Cy5.5-AlgMS and Cy5.5-CaAlgMS groups. However, the fluorescence signal was almost completely lost after 3 days in the free Cy5.5 group. *Ex vivo* imaging of major organs and tumors collected on day 9 (Fig. 4C and D) revealed detectable Cy5.5 signals in tumors from both the Cy5.5-AlgMS and Cy5.5-CaAlgMS groups. Notably, the signals in the Cy5.5-CaAlgMS group were stronger than those in the Cy5.5-AlgMS group, indicating that mineralization with CaCO_3_ further enhances local drug accumulation and retention within tumors.

CaCO_3_-based microspheres have been shown to act as neutralizing agents [21]. Our result further demonstrated that tumors treated with CaAlgMS-based microspheres exhibited significantly increased pH values compared with PBS, as evidenced by reduced SNARF-4F fluorescence upon 580 nm excitation (Fig. 4E and F). In contrast, injection of AlgMS did not lead to an increase in pH value. These findings highlight the dual function of RC6CaAlgMS in sustaining drug release while modulating the acidic TME. Considering that R848 actively induces the secretion of pro-inflammatory cytokines, including IL-6 and TNF-α, we systematically evaluated systemic inflammatory responses and general safety profiles in Hepa1-6 tumor-bearing mice following treatment with RCaAlgMS, RC6CaAlgMS, or free R848 at different doses. Serum levels of IL-6 and TNF-α were quantified by ELISA within 48 h after injection (Fig. S15). At the same R848 dose, mice treated with free R848 exhibited a sharp surge in both cytokines, which peaked at 3 h, rapidly declined within 12 h, and returned to baseline by 48 h. This acute and transient kinetic profile is highly indicative of rapid systemic dissemination of free R848 and the associated risk of acute systemic inflammatory toxicity. In contrast, both RCaAlgMS and RC6CaAlgMS led to a gradual and sustained elevation of serum cytokine levels throughout the 48h period, maintaining a mild yet biologically effective immunostimulatory state. This prolonged immune stimulation may support durable activation of both innate and adaptive immunity while minimizing the risk of excessive systemic inflammatory responses. Overall, these results indicate that RC6CaAlgMS shifts the immune response from a transient cytokine burst toward a sustained and controlled immunostimulatory state, thereby enhancing antitumor efficacy while reducing systemic inflammatory toxicity.

### 2.6 RC6CaAlgMS enhance the antitumor efficacy of chemotherapy *in vivo*

To better simulate potential clinical applications, we further explored the incorporation of the chemotherapeutic agent doxorubicin (DOX) into the RC6CaAlgMS system to achieve drug-loaded embolic microspheres. Following drug adsorption, DOX was successfully encapsulated within the microspheres, yielding the final formulation termed DRC6CaAlgMS. Compared with DRC6AlgMS (without CaCO_3_), DRC6CaAlgMS exhibited markedly faster drug loading, achieving a DOX encapsulation efficiency of 89.31 ± 2.62% within 10 min (Fig. S16A, B). Notably, both DRC6CaAlgMS and DRC6AlgMS retained a uniform spherical morphology after drug loading, with comparable swollen diameters of 74.52 μm and 73.59 μm, respectively (Fig. S16C). Drug release assays demonstrated that DRC6CaAlgMS exhibited a markedly accelerated DOX release rate under acidic conditions with 78.1 ± 5.5% released within 48 h (Fig. S16D), suggesting its strong pH-responsive release capability. These findings indicate that RC6CaAlgMS possesses excellent potential as a chemotherapeutic drug carrier, enabling effective drug-loaded chemoembolization therapy for HCC.

**Fig. 5:**
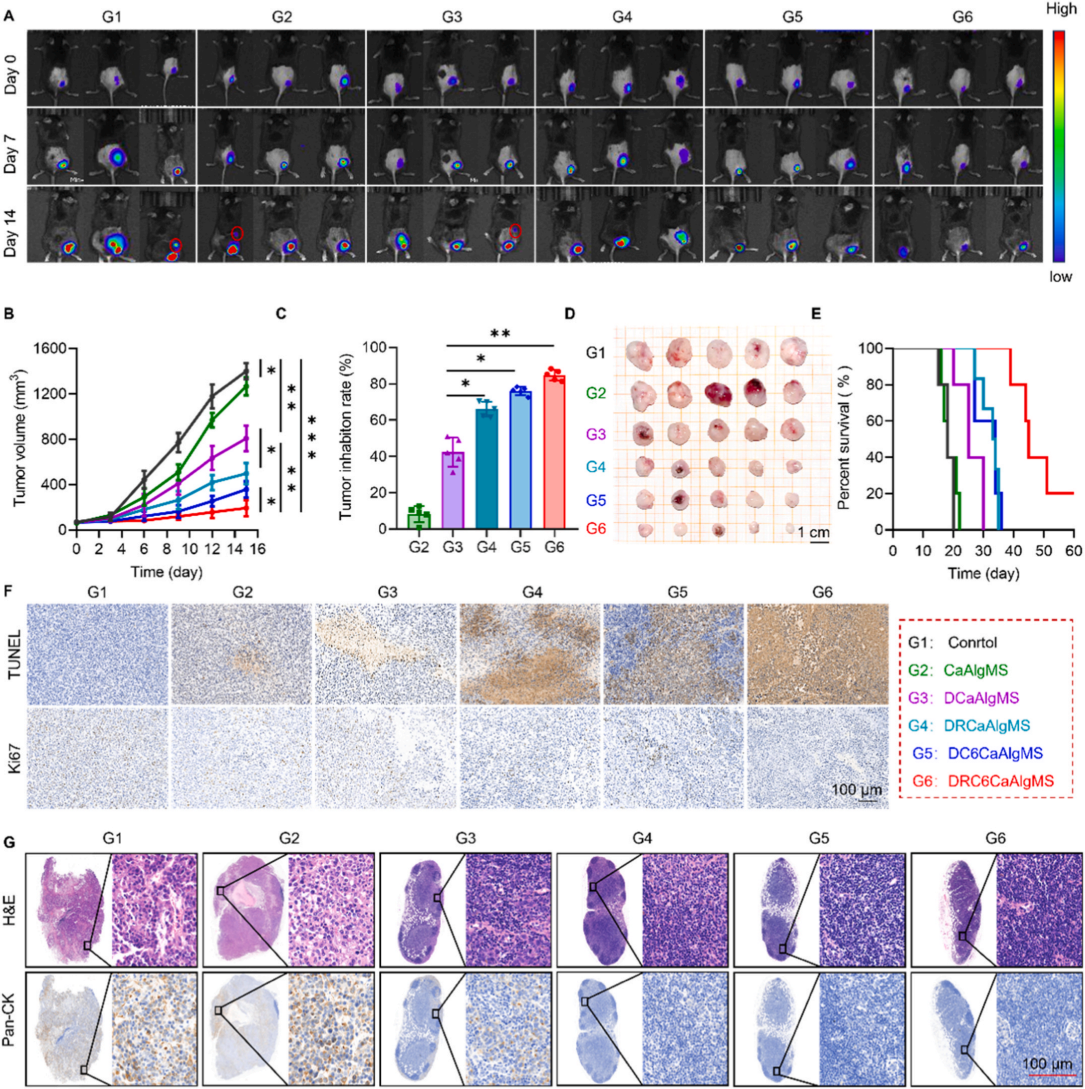
Evaluation of the antitumor efficacy of peritumoral therapy in a Hepa1-6-Luc HCC model. (A) *In vivo* bioluminescence images of representative mice from each treatment group at day 7 and 14 post-treatment, visualizing the growth of the primary Hepa1-6-Luc tumors. (B) Tumor growth curves monitored throughout the treatment period. (C) Quantitative analysis of tumor weights. Data are presented as mean ± SD (n = 5). (D) Representative photographs of excised tumors from Hepa1-6-Luc tumor-bearing mice after 15 days of treatment. (E) Kaplan-Meier survival curves of tumor bearing mice following different treatments (n = 5 mice per group). (F) Representative IHC of TUNEL assay for apoptosis and the proliferation marker Ki67 in tumor sections. Scale bar: 100 μm. Histological analysis of TDLNs. Representative images of H&E staining and pan-cytokeratin (pan-CK) IHC are shown. Scale bar: 100 μm. The statistical analysis was performed with ANOVA. P > 0.05 indicated no significance between two groups. *P < 0.05, **P < 0.01, ***P < 0.001.

Next, the antitumor efficacy of DRC6CaAlgMS was further evaluated in a subcutaneous Hepa1-6-luciferase (Hepa1-6-Luc) model. Mice with tumor volumes reaching 60-80 mm^3^, were assigned into six groups: G1, PBS; G2, CaAlgMS; G3, DCaAlgMS; G4, DRCaAlgMS; G5, DC6CaAlgMS; G6, DRC6CaAlgMS, with all formulations administered at a dose of 10 mg. Tumor growth was monitored using IVIS at designated time points post-treatment. As shown in Fig. 5 A and S17A, group G2 exhibited a slight but observable reduction in bioluminescence intensity and tumor volume compared to the PBS control group. This indicates that neutralizing the tumor acidic microenvironment with CaCO_3_ confers a certain degree of tumor growth inhibition. Compared with the PBS group, mice in group G3 exhibited significant tumor growth inhibition, an effect that may be attributed to the ability of CaCO_3_ microspheres to enhance chemotherapeutic efficacy, which is consistent with our previous findings. Although group G5 showed a non-significant reduction in tumor volume relative to group G4, potentially due to the direct tumoricidal effect of C6 noted earlier, the difference did not reach statistical significance. Furthermore, among all groups, tumors in group G6 exhibited the weakest and most confined bioluminescence signal, indicative of the superior antitumor efficacy. Notably, the DOX CaCO_3_-based microspheres co-loaded with both C6 and R848 demonstrated a significantly enhanced tumor suppression effect compared to the groups receiving either component alone (G4 and G5). Furthermore, tumor volumes measured every 3 days using a vernier caliper showed trends consistent with the bioluminescence imaging results. Compared to group G3, the tumor volume in group G4 and group G5 was reduced by approximately 1.69-fold and 2.4-fold, respectively (Fig. 5B). While mice in G6 exhibited the lowest tumor volume at day 15, corresponding to a tumor inhibition rate of 84.79% relative to the group G1. Moreover, the tumor volume in group G6 was 2.24-fold smaller than that in group G4 and 1.58-fold smaller than that in group G5 (Fig. 5C). On day 15, the mice were euthanized, and the tumors were excised, photographed, and weighed (Fig. 5D and S17B). The tumors in the group G6 were the smallest and lightest among all groups. These results highlights that the combined administration of DOX-loaded CaCO_3_ microspheres, R848, and C6 exerts a markedly superior antitumor efficacy compared with either treatment regimen. Additionally, Kaplan-Meier analysis revealed that mice in the DRC6CaAlgMS group exhibited significantly prolonged survival compared with the other groups (Fig. 5E). DRC6CaAlgMS group showed a marked improvement, with over 40% (2/5) of mice surviving beyond day 50. These findings highlight the synergistic effect of DRC6CaAlgMS in enhancing long-term therapeutic outcomes.

Besides, the histological damages evaluated using H&E analysis and TUNEL staining of tumor slices revealed severe tissue damages were observed in tumors from mice with DRC6CaAlgMS treatment and the percentage of tunnel positive cells in Group G6 was higher than those observed in the other groups (Fig. 5F and S18). Thus, our findings suggest that DRC6CaAlgMS has potent anticancer effect *in vivo*. In addition, Ki-67 immunohistochemical staining shows a small number of positive cells in Group 6, indicating low proliferative activity of the tumor (Fig. 5F). Approximately 46.97% of cells are Ki-67-positive G1 group, and the percentage of Ki-67-positive cells decreased to approximately 9.68% in G6 group. Notably, circular bioluminescent signals were detected in the right inguinal region of some mice from groups G1, G2 and G3 group (Fig. 5A), suggestive of potential metastasis to the tumor-draining lymph nodes (TDLNs). This observation prompted further investigation into the right-sided TDLNs in subsequent analyses. At the experimental endpoint, the right TDLNs were harvested from each group for H&E and pan-cytokeratin (Pan-CK) staining. As shown in Fig. 5G, the TDLNs from G1 and G2 groups were markedly enlarged and rounded, with substantial tumor cell infiltration observed in H&E-stained sections and a high Pan-CK-positive rate. Although the TDLNs in the G3 group largely maintained a normal morphology, both H&E and Pan-CK immunohistochemical staining revealed considerable tumor cell infiltration. In contrast, the TDLNs from G4, G5, and G6 groups exhibited essentially normal size and morphology, and no metastatic tumor cells were detected in the slices. These results indicate that the DOX CaCO_3_-based drug-loaded microspheres, whether incorporating C6 or R848 individually or in combination, significantly inhibit tumor cell metastasis to the TDLNs.

Results of our *in vitro* findings, RC6CaAlgMS promoted the repolarization of TAMs towards the M1 phenotype and enhanced their release of antigen-presenting exosomes. We therefore sought to validate the *in vivo* modulatory effect of DRC6CaAlgMS on exosome function. Plasma exosomes were isolated from mouse blood collected at the experimental endpoint. TAMs revealed that these exosomes exhibited typical cup-shaped structures enclosed by a lipid bilayer. DLS confirmed a mean particle size of 95.7 ± 5.2 nm (Fig. S19A), which was consistent with the electron microscopy observations.As shown in Fig. S19B, WB analysis of the isolated exosomes demonstrated a significant increase in the expression of exosomal marker proteins in the G4, G5, and G6 groups, with the most pronounced enhancement observed in the G5 and G6 groups. These results indicate that both DRC6CaAlgMS and DC6CaAlgMS potently stimulate exosome release. Interestingly, DRCaAlgMS treatment alone also moderately increased exosome levels. This effect may be attributed to the repolarization of TAMs towards the M1 phenotype. Furthermore, the documented role of R848 in promoting dendritic cell (DC) maturation may also contribute to the elevated plasma exosome levels following DRCaAlgMS treatment. Crucially, DRC6CaAlgMS treatment resulted in the most significant increase in exosome content in tumor-bearing mice. In addition, exosomes from the G4, G5, and G6 groups exhibited significantly upregulated expression of MHC-I and MHC-II molecules, but PD-L1 expression was markedly reduced. This protein profile suggests an enhancement in the antigen-presenting capacity of these exosomes and an amelioration of the immunosuppressive microenvironment.

To evaluate the *in vivo* biosafety of the treatments, body weight was monitored and major organs were harvested for histological examination (Fig. S20). No significant differences in body weight were observed among all groups throughout the study period. Furthermore, H&E staining revealed no obvious signs of tissue damage or pathological abnormalities in the examined organs compared with the G1 group. These initial findings indicate that the treatments were well-tolerated at the administered dosage. Collectively, these results demonstrate that DRC6CaAlgMS possesses a robust capacity to inhibit tumor growth and reduce metastatic spread to TDLNs which synergistically contributed to the prolonged survival of tumor-bearing mice. More importantly, DRC6CaAlgMS remodels the phenotype of plasma exosomes in tumor-bearing mice. This remodeling contributes to the reversal of the immunosuppressive TME, highlighting the multifaceted therapeutic potential of this strategy.

**Fig. 6:**
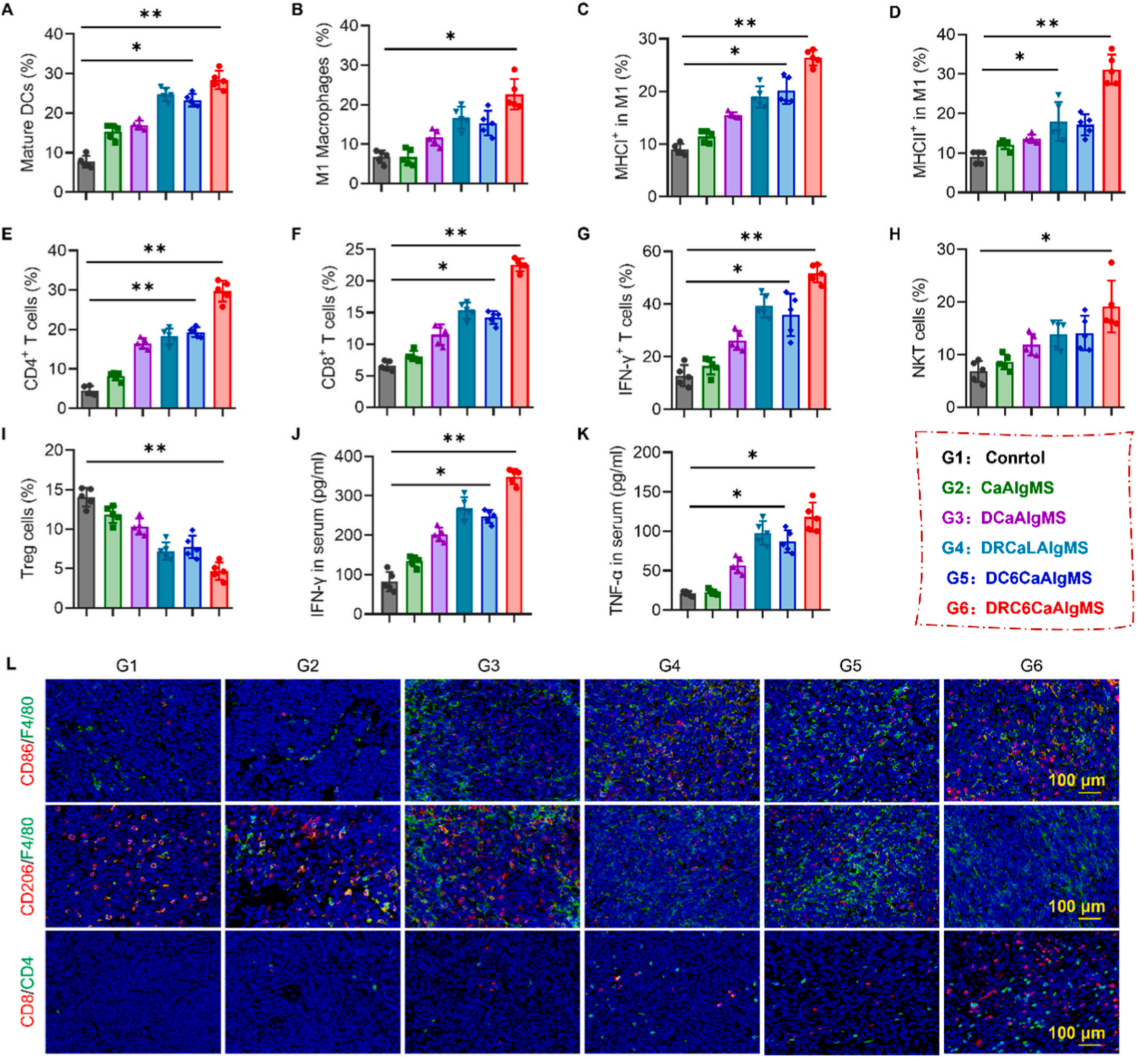
Flow cytometric analysis of immune cell infiltration in tumor tissues and draining lymph nodes after different treatments. (A) Representative quantification of mature DCs (CD80^+^CD86^+^DCs) in tumor-draining lymph nodes. Representative quantification of tumor-infiltrating immune cell subsets, including (B) M1-like macrophages, (C) MHC-I^+^ M1-like macrophages, (D) MHC-II^+^ M1-like macrophages (E) CD4^+^ T cells, (F) CD8^+^ T cells, (G) regulatory T cells (Tregs), (H) CD8^+^IFN-γ^+^ T cells and (I)natural killer T (NKT) cells. Data are presented as mean ± SD (n = 5 per group). Serum levels of IFN-γ (J)and TNF-α (K) detected by ELISA in mice from different treatment groups. (L) Representative images of tumor sections stained for (top) F4/80 CD86 (M1-like macrophages), (middle) F4/80 CD206 (M2-like macrophages), and (bottom) CD8 CD4 (T-cell subsets) in control (G1) and treated groups (G2-G6). Red, green, and blue channels indicate specific immune markers and nuclear counterstaining (DAPI). Scale bar = 100 μm. The statistical analysis was performed with ANOVA. P > 0.05 indicated no significance between two groups. *P < 0.05, **P < 0.01.

In our proposed therapy, DC6CaAlgMS regulated immune responses by inducing macrophages polarization and modulating macrophages exosome secretion. Therefore, various immune cells were first analyzed using FCM and immunofluorescence staining of tissue sections (Fig. 6). FCM analysis revealed that the proportion of mature dendritic cells (CD11c^+^CD80^+^CD86^+^) within TDLNs increased from 10.2% in G1 to 31.9% in G6, representing a 5.2-fold increase compared with the PBS group (Fig. 6A). Importantly, the proportion of MHC-I^+^ DCs increased to 16.74 ± 1.86%, which was 2.83 times higher than G1 group (Fig. S21A). In contrast, the percentage of Gr-1^+^ DCs decreased to 5.32 ± 0.30%, a value 1.36 times lower than that in the PBS-treated group, indicating enhanced antigen-presenting capacity (Fig. S21B). Similarly, FCM analysis demonstrated that the fraction of M1-like macrophages was significantly elevated in the G6 group compared to the G1 group (22.62 ± 3.60% vs. 6.85 ± 1.51%, p < 0.05), accompanied by a marked upregulation of MHC-I and MHC-II expression (Fig. 6B). Notably, quantitative analysis revealed that the proportion of MHC-I^+^ M1-like macrophages increased by approximately 2.93-fold (P < 0.001) (Fig. 6C), while MHC-II^+^ M1-like macrophages increased by 3.46-fold (P < 0.001) compared with the G1 group (Fig. 6D). Whereas M2-like macrophages were relatively decreased (Fig. S21C), suggesting successful macrophages repolarization. Additionally, the activated T cell populations (CD4^+^CD8^+^ T cells) in tumor tissues after different treatments were examined. The population of tumor‐infiltrating CD4^+^ T cells in G6 group also increased 2.83 times higher than G1 group (Fig. 6E). The population of CD8^+^ T cells in the tumors after DRC6CaAlgMS treatment was 6.60, 3.65, 1.80, 1.63, and 1.54‐fold higher than those in mice treated with PBS, CaAlgMS, DCaAlgMS, DRCaAlgMS, and DC6CaAlgMS, respectively (Fig. 6F). Correspondingly, there was an increase in the proportion of functional CD8^+^ IFN-γ^+^ T cells from 12.78 to 51.62% in the G6 group (Fig. 6G). The number of NK T cells, which are innate immune cells, was increased, although the difference was not dramatically significant (Fig. 6H). In contrast, the tumoral infiltration of regulatory T cells (Foxp3^+^ T cells, Fig. 6I), which represent the immunosuppressive TME, dramatically decreased upon DC6CaAlgMS treatment. Overall, these findings demonstrated that DC6CaAlgMS was capable of promoting immune cell maturation, inducing the production of active T cells, and reshaping the TME into an immune-activating state. Consistent with this immune activation, the cytokine secretion, including TNF-α and IFN-γ in serum, was correspondingly increased upon the DC6CaAlgMS treatment (Fig. 6J and K), indicating a robust antitumor immune response.

Furthermore, the results from immunofluorescence staining further confirmed the above findings: M1-like macrophages that were positive for F4/80 and CD86 staining were increased in tumor sections from DRC6CaAlgMS-treated mice, while M2-like macrophages infiltrating into tumor sections were significantly reduced by DRC6CaAlgMS treatment. As expected, the treatment of DRC6CaAlgMS led to the higher degree of CD4^+^ cells and CD8^+^ cells infiltration in the tumors compared to all other groups (Fig. 6L), Taken together, these results indicated that DRC6CaAlgMS exerted a strong antitumor immune response in liver cancer, relieving immunosuppression and recruiting immunostimulatory cells to remodel the TME.

To determine whether the therapeutic efficacy of DRC6CaAlgMS depends on the host immune system, we evaluated its antitumor activity in tumor-bearing mice following the depletion of specific immune cell populations. Isotype controls and Sham-Lip served as negative controls, we found that the potent tumor suppression mediated by DRC6CaAlgMS was completely abolished upon depletion of either CD8^+^ T cells or macrophages, indicating a critical reliance on these immune subsets. As shown in Fig. S22, mice treated with DRC6CaAlgMS plus Isotype or Lip exhibited profound inhibition of tumor growth, with a tumor growth inhibition rate of 87.72% and 84.80% respectively. In stark contrast, this therapeutic benefit was lost when DRC6CaAlgMS was administered in combination with CD8^+^ T cell-depleting antibodies, resulting in rapid tumor regrowth. A nearly identical reversal of efficacy was observed following macrophages depletion. These data provide direct evidence that the efficacy of DRC6CaAlgMS is not solely attributable to a direct cytotoxic effect but critically depends on a functional host immune system. The observation that depletion of either innate or adaptive immune components similarly abrogates treatment efficacy strongly suggests that the antitumor functions of DRC6CaAlgMS-reprogrammed macrophages and the cytotoxic activity of activated CD8^+^ T cells are both essential for the therapeutic success of this strategy.

### 2.7 RC6CaAlgMS potentiates chemoimmunotherapy *In vivo*

**Fig. 7:**
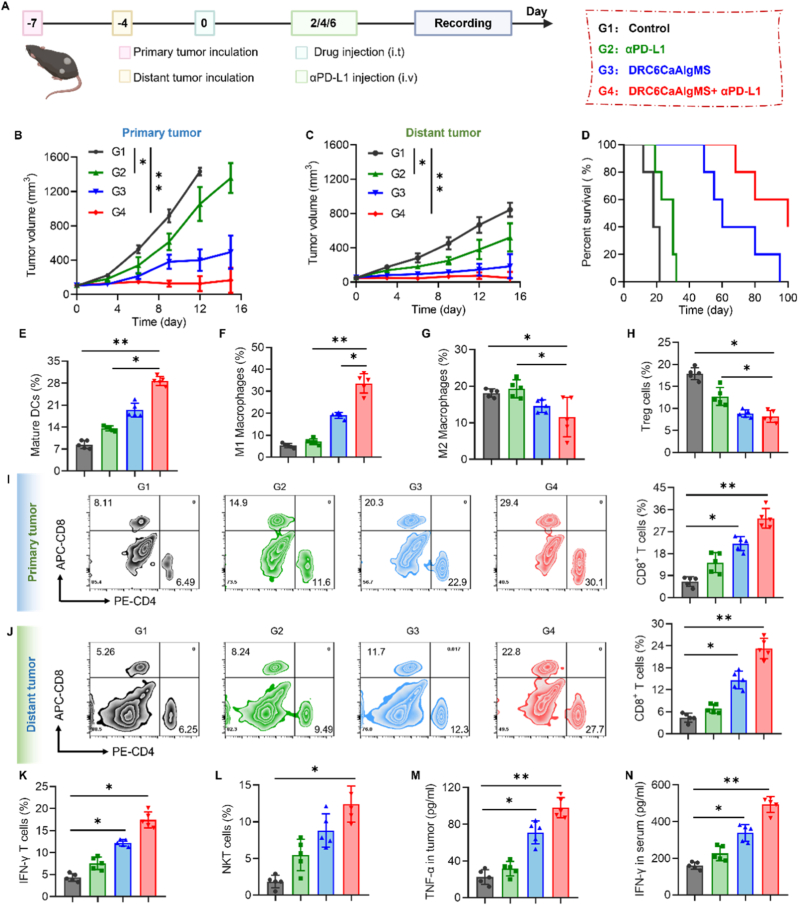
Evaluation of the abscopal effect and combination therapy in a bilateral Hepa1-6 tumor model. (A) Schematic illustration of the bilateral tumor model establishment and treatment timeline. Mice were inoculated with Hepa1-6 cells on the right flank on day −7 and on the left flank on day −3 to establish primary and distant tumors, respectively. On day 0, mice were randomized and treated with peritumoral injection of DRC6CaAlgMS near the primary tumor. αPD-L1 was administered via tail vein injection on days 0, 2, and 4. Growth curves of the primary tumors (B) and distant tumors (C) in different treatment groups. (D) Survival curves of mice bearing bilateral tumors in the different treatment groups. (E) Representative quantification of mature DCs (CD80^+^CD86^+^DCs) in TDLNs. Representative quantification of primary tumor-infiltrating immune cell subsets, including (F) M1-like macrophages, (G) M2-like macrophages, (H) Tregs, (K) IFN-γ^+^ T cells and (L) NKT cells. Representative FCM plots (left) and semi-quantitative analysis (right) of CD3^+^CD8^+^ T cells in primary tumor (I) and distant tumor(J). Serum levels of IFN-γ (M) and TNF-α (N) detected by ELISA in mice from different treatment groups. The statistical analysis was performed with ANOVA. P > 0.05 indicated no significance between two groups. *P < 0.05, **P < 0.01.

Subsequently, we rigorously evaluated the therapeutic efficacy of a combined regimen involving peritumoral DRC6CaAlgMS and systemic anti-PD-L1 antibody (αPD-L1) in a challenging bilateral Hepa1-6 tumor model, which recapitulates metastatic disease. In this model, a primary tumor was established on the right flank, followed by inoculation of a secondary, distant tumor on the left flank four days later. When the primary tumor volume reached approximately 100 mm^3^, mice were randomized into four treatment cohorts: (a) PBS control, (b) αPD-L1 (2 mg/kg), (c) DRC6CaAlgMS (10 mg per mouse), and (d) DRC6CaAlgMS+ αPD-L1 (Fig. 7A). Treatment with DRC6CaAlgMS alone resulted in a pronounced suppression of primary tumor growth, reducing the growth rate by approximately 79.8% compared to the PBS control by day 15. Strikingly, the combination therapy yielded a near-complete arrest of primary tumor progression, with final tumor volumes measuring less than 50 mm^3^, which was significantly lower than those in either monotherapy (Fig. 7B). Notably, this potent antitumor effect extended abscopal to the distant, untreated tumors. The inhibition rate of distant tumors surged from a modest 38.2% in the αPD-L1 alone group to a remarkable 89.8% in the combination group. Furthermore, complete regression of distant tumors was observed in one out of five mice (20%) receiving DRC6CaAlgMS alone and in three out of five mice (60%) receiving the combination therapy, with no recurrence during the observation period (Fig. 7C). Consequently, mice in the combination group exhibited a significant survival advantage, with a median survival exceeding 65 days, compared to 22 days for the PBS group and 30 days for the αPD-L1 monotherapy group (Fig. 7D). Critically, no significant body weight loss or other overt signs of toxicity were detected, underscoring the favorable safety profile of the combined treatment (Fig. S23).

To decipher the immunomodulatory mechanisms underlying this robust efficacy, we performed comprehensive immune profiling. FCM analysis of distant tumor-draining lymph nodes on day 5 post-treatment revealed that the combination therapy most effectively promoted dendritic cell maturation, with a frequency of mature DCs reaching 28.2%. This was substantially higher than the frequencies in the DRC6CaAlgMS (20.5%), αPD-L1 (14.5%), and PBS (9.8%) groups (Fig. 7E and S24A). Within the TME of distant lesions, the combination strategy drove a profound pro-inflammatory shift. The proportion of tumor-infiltrating M1-like macrophages increased dramatically to 33.6%, while the immunosuppressive M2-like macrophages population was concurrently suppressed to 11.6%, resulting in a highly favorable M1/M2 ratio of approximately 2.9 (Fig. 7F and G and S24B). This was accompanied by a massive influx of cytotoxic T lymphocytes (CTLs), in the primary tumors, CD3^+^CD8^+^ T cells accounted for 29.4% (Fig. 7I). Strikingly, this effect extended to distant tumors, where the infiltration of CD3^+^CD8^+^ T cells reached 20.1% (Figs. 7J), and 20.1% of these cells were highly functional, as indicated by the population of CD8^+^IFN-γ^+^ T cells (Fig. 7K and S24C). In stark contrast, the population of immunosuppressive CD4^+^Foxp3^+^ regulatory T cells (Tregs) were reduced by more than 50% compared to the control group (Fig. 7H and S24D). The innate immune response was also potentiated, as evidenced by a significant increase in the infiltration of NKT cells within the tumors of the combination group (Fig. 7L). Consistent with these cellular findings, enzyme-linked immunosorbent assays confirmed elevated secretion of key antitumor cytokines in distant tumors, with IFN-γ and TNF-α concentrations being 3.5-fold and 2.8-fold higher, respectively, in the combination group than in the PBS control (Fig. 7M and N). A similar enhancement of CD8^+^ T cell infiltration was observed in the primary tumors, indicating the successful establishment of systemic and durable antitumor immunity. Collectively, these data demonstrate that DRC6CaAlgMS acts as a powerful chemo-immunotherapy agent that synergizes with PD-L1 blockade to reinvigorate both innate and adaptive immune responses, leading to potent local and abscopal tumor regression.

### 2.8 Embolization performance of RC6CaAlgMS

**Fig. 8:**
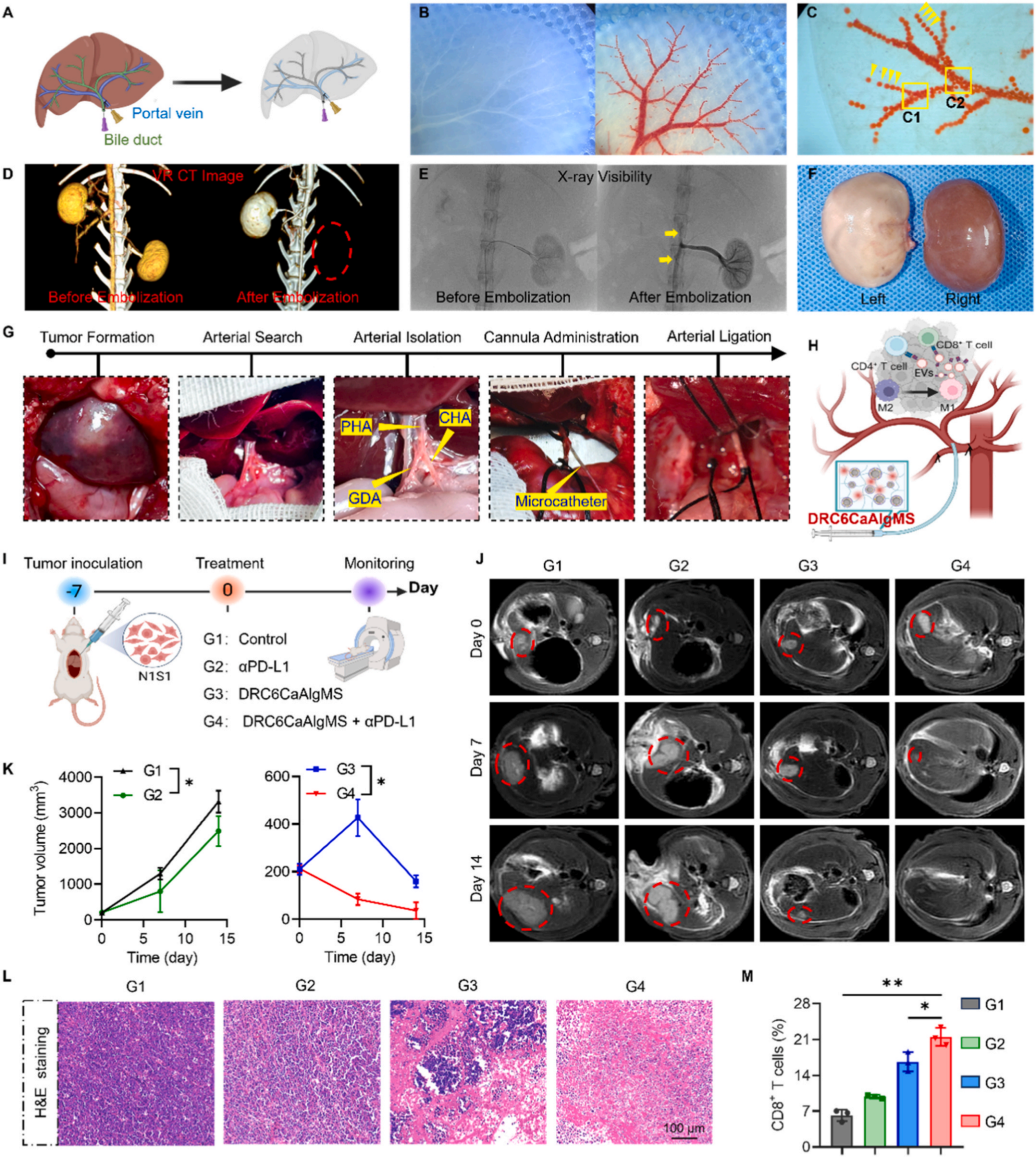
Establishment of an *ex vivo* decellularized liver model and *in vivo* evaluation of TAE therapy in a rat HCC model. (A) Schematic diagram illustrating the preparation process of the decellularized rat liver. (B) Characterization of the decellularized liver scaffold and microsphere distribution. (Left) Photograph of a decellularized rat liver, showing a transparent appearance with a clear preservation of the intrahepatic vascular architecture. (right) The decellularized liver after injection of DRC6CaAlgMS microspheres via the portal vein, demonstrating the distribution of microspheres within the vascular network. (C) High-magnification views showing DRC6CaAlgMS microspheres arranged in an end-to-end (C1) and side-by-side (C2) manner within the vascular channels. Yellow triangles indicate the microspheres. (D) Radiological assessment of renal embolization. CTA images of the kidney before and after CaAlgMS injection, showing successful vascular occlusion. (E) DSA images before and after embolization. The post-embolization image shows contrast reflux (arrow), indicating successful vascular blockage and demonstrating good embolization efficacy. (F) Macroscopic appearance of rabbit kidneys after embolization. (Left) Embolized kidney showing pale appearance indicative of successful ischemia. (Right) Non-embolized contralateral kidney showing normal appearance. (G) Photograph illustrating the actual TAE procedure performed on a rat. (H) Schematic illustration of the proposed mechanism after embolization of DRC6CaAlgMS. (I) Schematic diagram of the *in vivo* therapeutic timeline. SD rats were orthotopically inoculated with N1S1 cells in the liver on day −7. On day 0, baseline tumor size was measured by MR imaging, followed by the TACE procedure. MR imaging was performed weekly to monitor tumor progression. (J) Representative longitudinal MR images of liver tumors in rats from different groups before and after treatments, showing the changes in tumor size over time. (K) Tumor growth curves monitored throughout the treatment period. The tumor growth curves for the G1 and G2 group (left) and average tumor growth curves for the rats after DRC6CaAlgMS (25 mg per rat) and DRC6CaAlgMS +αPD-L1 (2 mg/kg) treatment (right). (L) Microscopy images of H&E-stained tumor slices from different treated groups. Scale bar = 100 μm. (M) Statistical comparison of the mean MFI for tumor-infiltrating CD3^+^CD8^+^ T cells among different treatment groups. The statistical analysis was performed with ANOVA. P > 0.05 indicated no significance between two groups. *P < 0.05, **P < 0.01.

Having validated the antitumor efficacy and immunomodulatory effect of DRC6CaAlgMS, we then investigated the embolization efficacy of RC6CaAlgMS using a decellularized organ model [13]and a rabbit renal artery embolization model [46]. As shown in Fig. 8A and S25, a decellularized organ model was constructed and validated by H&E staining. Hepatocytes were effectively removed from the liver tissue, and an intact acellular extracellular matrix was obtained (Fig. 8B). The doxorubicin-loaded microspheres DRC6CaAlgMS could be facilely delivered into the portal venous vasculature in a decellularized liver model, presenting full occlusion with compact arrangement in both distal and proximal vessel branches of varying sizes (Fig. 8B). In addition, spherical microspheres with uniform particle size were aligned end-to-end within terminal vascular branches, while in larger-diameter vessels they arranged side-by-side, forming intimate interactions with the vessel wall (Fig. 8C). These results indicate that the uniformly sized microspheres achieved excellent embolization efficacy. For validation, the embolization efficacy was tested using a rabbit renal model. It was found that CaAlgMS could easily pass through the microcatheter and be injected into the left renal artery under the guidance of DSA. As shown in Fig. 8E, the blood vessels of the left kidney were clearly visible before injection of CaAlgMS. In contrast, the vascular network of the left kidney vanished after the injection of CaAlgMS, which was due to the fact that the contrast agent iodixanol could not diffuse into the arteries of the embolized left kidney. The embolic efficacy of CaAlgMS was also evaluated using computed tomography angiography (CTA) after 2 weeks of embolization. It was clearly shown that the left kidney was not visible from the CTA photos, which suggested that the blood vessels of the left kidney were still firmly embolized by CaAlgMS. Gross examination at day 14 (Fig. 8D) showed that the embolized kidney appeared pale and shrunken compared with the contralateral control, indicative of ischemic damage. Correspondingly, H&E staining revealed widespread parenchymal necrosis and tubular structural collapse in the embolized kidney, while the contralateral kidney remained histologically intact (Fig. 8F and S26A). Importantly, histological evaluation of other major organs including the liver, spleen, and lung, showed no overt pathological changes (Fig. S26B). These results indicated the high efficacy and biosafety of RC6CaAlgMS for TAE.

### 2.9 RC6CaAlgMS increase the therapeutic efficacy of TACE

The therapeutic efficacy of RC6CaAlgMS-based TACE was tested using a rat orthotopic N1S1 liver cancer model. Rat orthotopic liver tumor models were constructed by injecting an N1S1 cell suspension into the liver lobe after laparotomy. One week following tumor inoculation, orthotopic N1S1 liver tumors with an average size of ≈250 mm^3^ were successfully formed, and the rats were then randomly divided into four treatment groups: 1) control, 2) αPD-L1 (2 mg/kg, i.v.), 3) DRC6CaAlgMS, and 4) DRC6CaAlgMS+αPD-L1(Fig. 8I). All formulations were administered via TACE in a total mass of 25 mg per rat. The embolization procedure was performed in the same manner for all groups and the retrograde gastroduodenal artery was selectively infused with these embolic agents (Fig. 8G and H). In combination with αPD-L1, DRC6CaAlgMS TACE exhibited the strongest therapeutic effect, as evidenced by a lower tumor volume compared with either DRC6CaAlgMS-TACE or αPD-L1 alone (Fig. 8J and K). Notably, one rat (33.3%) exhibited a complete response, characterized by the disappearance of the hepatic tumor and no evidence of recurrence throughout the study period. H&E staining at 1 week indicated that compared with the other treatment groups, the DRC6CaAlgMS group presented the highest level of tissue necrosis (Fig. 8L).

To further decipher the immune landscape, immunofluorescence analysis was performed. Quantitative analysis of fluorescence intensity revealed that DRC6CaAlgMS treatment alone significantly promoted the infiltration of antitumor immune cells compared to controls. This effect was substantially augmented in the combination group. Specifically, the fluorescence signal for M2-like macrophages (F4/80^+^CD206^+^) was significantly attenuated by DRC6CaAlgMS treatment, and this suppression was further enhanced by the addition of αPD-L1(Fig. S27A). In contrast, the fluorescence signal for M1-like macrophages (F4/80^+^CD86^+^) was markedly elevated following DRC6CaAlgMS treatment, and this increase was further amplified upon combination with αPD-L1(Fig. S27B). Consistent with immunofluorescence observations, qPCR analysis further confirmed that DRC6CaAlgMS treatment alone significantly upregulated M1 marker gene iNOS and downregulated the M2 marker gene Arg-1 in the tumor (Fig. S28A and 28B). This polarization shift was further potentiated in the DRC6CaAlgMS + αPD-L1 combination group. Moreover, analysis of the tumor cytokine milieu revealed that the levels of IL-12 were notably increased in the DRC6CaAlgMS-treated group relative to the control (Fig. S28C). This elevation was further amplified in the combination group. Conversely, the levels of the anti-inflammatory cytokine IL-10 were markedly decreased following DRC6CaAlgMS + αPD-L1 treatment (Fig. S28D). These results indicate a successful reprogramming of the tumor immune microenvironment towards an antitumor state. Furthermore, DRC6CaAlgMS treatment alone induced a moderate increase in CD4^+^ T cell fluorescence intensity, an effect that was markedly elevated in the combination group (Fig. S27C). This suggests that DRC6CaAlgMS+αPD-L1 treatment enhances the helper function of CD4^+^ T cells, which is crucial for sustaining CD8^+^ T cell responses. Finally, as shown in Fig. S27D and Fig. 8M, the DRC6CaAlgMS-treated group exhibited a substantial increase in CD8^+^ T cell infiltration, with the combination therapy yielding the highest fluorescence intensity and demonstrating a synergistic effect on cytotoxic T lymphocyte recruitment and activation. Concurrently, blood biochemical parameters in rats revealed no significant abnormalities (Fig. S29). indicating the biocompatibility, efficient degradation, and excretion of DRC6CaAlgMS. Taken together, these findings verify that DRC6CaAlgMS successfully remodels the TME into an immunostimulatory state, which is synergistically amplified by αPD-L1 to potentiate antitumor immunity.

## 3 Conclusion

In this study, we identify macrophage polarization and exosome-mediated antigen as critical determinants governing the therapeutic efficacy of embolization-immunotherapy in HCC. We observed that TACE profoundly reshapes macrophage function within the TAM, and the extent of macrophage infiltration is closely associated with therapeutic responsiveness to TACE. The combination of C6 and R848 synergistically reprograms TAMs toward an immunostimulatory phenotype while enhancing antigen presentation and initiating immune activation. To enable efficient integration of the functions of C6 and R848, we engineered RC6CaAlgMS as a multifunctional embolic platform. RC6CaAlgMS effectively neutralizes the acidic TME, thereby creating a permissive niche for antitumor immune activation. In parallel, RC6CaAlgMS reprograms TAMs, reverses post-TACE immunosuppression, and enhances macrophage antigen-presenting capacity as well as T-cell effector function. When combined with chemotherapy, RC6CaAlgMS elicits potent antitumor efficacy accompanied by substantial remodeling of the tumor immune microenvironment. By coordinating macrophage reprogramming and exosome-mediated antigen dissemination, RC6CaAlgMS effectively sensitizes tumors to PD-L1 blockade, resulting in enhanced systemic tumor control and a pronounced abscopal-like effect. This work establishes a biomaterial-enabled locoregional immunotherapy strategy capable of converting embolization-induced immunosuppression into systemic antitumor immunity. Collectively, this study presents a macrophage-centered immunomodulatory strategy that integrates tumor acidosis neutralization, TAM reprogramming, and exosome-mediated antigen cross-presentation to overcome post-embolization immune suppression. Beyond HCC, this biomaterial-engineered platform may provide a broadly translatable framework for enhancing embolization-based immunotherapy in solid tumors.

## CRediT authorship contribution statement

**Xiaoju Guo:** Conceptualization, Formal analysis, Investigation, Methodology, Project administration, Validation, Visualization, Writing – original draft, Writing – review & editing. **Shiji Fang:** Conceptualization, Funding acquisition, Investigation, Methodology, Project administration, Software, Visualization, Writing – review & editing. **Liyun Zheng:** Data curation, Formal analysis, Funding acquisition, Project administration, Resources, Validation. **Mengzhu Han:** Investigation, Validation, Visualization. **Yiming Ding:** Formal analysis, Validation, Visualization. **Jiale Chen:** Validation, Visualization. **Pan Qin:** Investigation, Validation. **Yeyu Zhang:** Formal analysis, Software. **Gaofeng Shu:** Conceptualization, Writing – review & editing. **Zhongwei Zhao:** Resources, Supervision. **Xiaoqiang Li:** Supervision, Writing – review & editing. **Chenying Lu:** Project administration, Resources, Supervision. **Minjiang Chen:** Project administration, Resources, Supervision, Writing – review & editing. **Jiansong Ji:** Project administration, Resources, Supervision, Writing – review & editing.

## Declaration of competing interest

The authors declare no competing interests.

## Data Availability

Data will be made available on request.
