# Supplementary material for: Macrophage-reprogramming calcium alginate microspheres enhance exosome-mediated antigen cross-presentation to boost embolization-immunotherapy in hepatocellular carcinoma
Source: Mater Today Bio. 2026 Jul 18;39:103459. doi: 10.1016/j.mtbio.2026.103459 (PMC13449422; doi:10.1016/j.mtbio.2026.103459)
Supplement: Supplementary file 1 [file mmc1.pdf]

Supporting Information

Macrophage-Reprogramming Calcium Alginate Microspheres Enhance Exosome-Mediated Antigen Cross-Presentation to Boost Embolization-Immunotherapy in Hepatocellular Carcinoma

Xiaoju Guo[1,2,3#], Shiji Fang[1,3#], Liyun Zheng[1,3], Mengzhu Han[1], Yiming Ding[1], Jiale Chen[1], Pan Qin[1], Yeyu Zhang[1,3], Gaofeng Shu[1,3], Zhongwei Zhao[1,3], Xiaoqiang Li[4*], Chenying Lu[1,2,3*], Minjiang Chen[1,2,3*], Jiansong Ji[1,2,3*]

[1] Zhejiang Key Laboratory of Imaging and Interventional Medicine, the Fifth Affiliated Hospital of Wenzhou Medical University, Lishui 323000, China.

[2] Department of Radiology, Lishui Central Hospital, the Fifth Affiliated Hospital of Wenzhou Medical University, Lishui 323000, China.

[3] Cancer center, Lishui Central Hospital, the Fifth Affiliated Hospital of Wenzhou Medical University, Lishui 323000, China.

[4] Suzhou Institute of Biomedical Engineering and Technology, Chinese Academy of Sciences, Suzhou 215163, China.

[#]*Xiaoju Guo and Shiji Fang equally contributed to this work.*

**Corresponding author.*

Jiansong Ji, MD, PhD, Zhejiang Key Laboratory of Imaging and Interventional Medicine, Fifth Affiliated Hospital of Wenzhou Medical University, No 289, KuoCang road, Lishui 323000, China. Tel.: +86 0578 2285011. E-mail: jjstcty@wmu.edu.cn

Minjiang Chen, PhD, Zhejiang Key Laboratory of Imaging and Interventional Medicine, Fifth Affiliated Hospital of Wenzhou Medical University, No 289, KuoCang road, Lishui 323000, China. Tel.: +86 0578 2285011. E-mail: minjiangchenv@163.com

Chenying Lu, PhD, Zhejiang Key Laboratory of Imaging and Interventional Medicine, Fifth Affiliated Hospital of Wenzhou Medical University, No 289, KuoCang road, Lishui 323000, China. Tel.: +86 0578 2285011. E-mail: luchenying@wmu.edu.cn

Xiaoqiang Li，PhD， Suzhou Institute of Biomedical Engineering and Technology, Chinese Academy of Sciences, Suzhou 215163, China. Tel.: +86 0578 2285011. E-mail: lixq@sibet.ac.cn

EXPERIMENTAL SECTION

Materials

C6-Ceramide (C6) and R848 were procured from Med Chem Express (MCE, Shanghai, China). Doxorubicin hydrochloride (DOX). Sodium alginate (Alg), sodium bicarbonate ($NaHCO_3$), calcium chloride ($CaCl_2$), sodium carbonate (Na_2CO_3), glutaraldehyde, Span 80, isopropanol, liquid paraffin, n-hexane, and calcium iodide were all purchased from Aladdin Bio-Chem Technology Co., Ltd. (Shanghai, China). All other chemicals were of analytical grade. Lipopolysaccharide (LPS) were purchased from Sigma-Aldrich. Exosomes Precipitation Kit was obtained from Solarbio Biotechnology (Beijing, China). DAPI was obtained from Sigma-Aldrich. The following flow cytometry (FCM) antibodies were sourced from BioLegend, Inc. (CA, USA): PerCP/Cyanine5.5 anti-mouse CD11b antibody (#101228), PerCP/Cyanine5.5 anti-mouse CD11c antibody (#117328), APC anti-mouse CD80 (#104714), FITC anti-mouse CD86 (#105006), PE/Cyanine7 anti-mouse CD45 (#157206), PE anti-mouse F4/80 (#111704), APC anti-mouse CD206 (t# 141708), APC anti-mouse CD3 (#100236), PE anti-mouse CD8a (# 100706), FITC anti-mouse CD4 (#130308), PerCP/Cyanine5.5 anti-mouse NK-1.1 (#156526), FITC- anti-mouse MHC I (#114605), PE- anti-mouse MHC II (#107607), APC- anti-mouse Gr1 (#108411), APC-anti-mouse CD25(#113709), PE-anti-mouse Foxp3(#126404),and APC-anti-mouse IFNγ(#505810). The anti-PD-L1 (αPD-L1) antibody was acquired from Biocell (CA, USA). Quantikine ELISA Kits for TNF-α, IL12, Granzyme B and IFN-γ were purchased from Enzyme-free Industrial Co., Ltd. (Jiangsu, China). Mouse M-CSF, IL-4, IL10, and IFNγ were purchased from Proteintech (Wuhan, China). Anti-iNOS antibody (# ab178945) was from Abcam. Anti-Arginase-1 (Arg-1) antibody (# ab185878) was from Abcam. Anti-Alix (clone 3A9; 2171), anti-CD63 (Clone H5C6, 353007), anti-PD-L1 (Clone 10F.9G2, 124307) were purchased from Proteintech. Anti-MHC I (# 311405), and anti-MHC II (# 307605) antibodies were obtained from Cell Signaling Technology. The MTT (3-(4,5-Dimethyl-2-thiazolyl)-2,5-diphenyl-2-H-tetrazolium bromide) assay kit (ST316), annexin V-FITC apoptosis detection kit

(C1062L), and BCA protein assay kit (P0009) were all obtained from Beyotime Biotechnology (Shanghai, China).

Cell Lines and Animals

The murine Hepa1-6 and rat N1S1 hepatocellular carcinoma cell lines were obtained from ATCC. A stable luciferase-expressing Hepa1-6 (luc-Hepa1-6) subline was generated for this study. All cells were cultured in complete DMEM medium at 37°C with 5% CO_2.

Male C57BL/6 mice and Sprague-Dawley rats were used in accordance with protocols approved by the Fifth Affiliated Hospital of Wenzhou Medical University Animal Ethics Committee. Tumor models were established as follows: (a) Unilateral subcutaneous model: luc-Hepa1-6 cells were injected into the right flank. (b) Bilateral subcutaneous model: wild-type Hepa1-6 cells were injected into the right flank (day -7) and left flank (day -4). (c) Orthotopic model: N1S1 cells were injected into the liver lobe of anesthetized rats. Mice and rats were humanely euthanized at the experimental endpoint in accordance with institutional animal care guidelines.

Analysis of the Tumor Immune Microenvironment by Single-Cell RNA Sequencing

This study conducted a systematic analysis of the changes in the tumor immune microenvironment (TIME) of HCC before and after TACE, based on the single-cell RNA sequencing (scRNA-seq) data published by Tan et al. (2023). The analytical workflow was as follows: First, we performed an integrated analysis of scRNA-seq data from 54,552 immune cells obtained from a discovery cohort comprising five primary tumors (PT) and five TACE-treated tumors (TT). t-SNE was employed for dimensionality reduction and visualization, which clearly revealed global shifts in immune cell composition following TACE treatment. Unsupervised clustering identified nine distinct immune cell subsets, including B cells, $CD4^+$ T cells, $CD8^+$ T cells, NK cells, dendritic cells, monocytes, M1 macrophages, M2 macrophages, and plasma cells. These subsets were confidently annotated based on the expression of well-established marker genes. To quantitatively assess the changes in immune cell proportions, comparative analysis between groups was performed using box plots. The gene expression dataset GSE104580 was retrieved from the Gene Expression Omnibus

(GEO) database. This dataset comprises microarray data from 147 tumor samples, classified into 81 TACE responders and 66 non-responders. Patients were stratified into respond and non-respond groups based on their clinical response to TACE. The CIBERSORT algorithm was employed to deconvolute the gene expression matrix and estimate the relative abundance of 22 immune cell types within each sample. All statistical analyses and result visualizations were performed using R studio (version 4.4.1).

***In vitro* cytotoxicity of C6 and R848**

In vitro cytotoxicity of C6 or R848 towards Hepa1-6 cells M1 Macrophage cells, and M2 Macrophage cells was evaluated by MTT assay. In brief, cells were seeded into 96-well plates at a density of 1×10^4 cells per well. After 12 h incubation, the cells were exposed to varying doses of C6 or R848 for 24 h. Subsequently, 20 μL MTT solution (5 mg/mL) was introduced to each well, which was incubated for 4 hours at 37°C to allow formazan crystal formation. Following removal of the medium carefully, and dimethyl sulfoxide (DMSO, 150 μL per well) was added to dissolve the crystals. The absorbance of each sample was then measured at 570 nm utilizing an automated microplate reader. Then, the viabilities were calculated using the following formula: Cell viability (%) = Abstestcells/Abscontrolcells×100%.

Isolation and polarization of mouse BMDM

Bone marrow cells were isolated from the femurs and tibias of C57BL/6 mice, and differentiated in complete DMEM medium supplemented with 50 ng/mL M-CSF. On day 7, BMDM were treated with LPS (100 ng/mL) plus IFN-γ (40 ng/mL) for 24 h to generate M1-like macrophages. And IL-4 (20 ng/ml) and IL-10 (20 ng/ml) was used to derived BMDM to M2-like macrophages.

Transmission electron microscopy of M1 and M2 Macrophage-Derived Exosomes

10×10^6 M1- or M2-like macrophages were activated and exosomes isolated from the conditioned supernatant using an Exosomes Precipitation Kit (Solarbio, EX0016) according to the manufacturer's instructions. In brief, the supernatant was first centrifuged at 2,000 g for 30 minutes to remove cells and debris. The resulting supernatant was transferred to a new tube and mixed thoroughly with the exosome

precipitation reagent at a volume ratio of 5:1 (supernatant: reagent). The mixture was incubated at 4 °C overnight. Following incubation, the sample was centrifuged at 13,000 g for 60 minutes at 4 °C to pellet the exosomes. The supernatant was carefully discarded, and the exosomes pellet was resuspended in an appropriate volume of PBS for applications. Exosomes characterization: The size distribution of the isolated exosomes was determined by DLS using a Malvern mastersizer 2000 (Malvern, UK). The morphology of exosomes was examined by TEM (Hitachi HT7800).

TEM analysis of ILVs and MVBs in macrophages.

Following polarization, M1- and M2-like Macrophages were subjected to various treatments with exosomes-depleted FBS for 24 hours: (a) vehicle control, (b) R848 (1 μg/mL), (c) C6 (4 μg/mL), and (d) R848+C6（1 μg/mL and 4 μg/mL, respectively). The cells were collected and immediately fixed with 2.5% glutaraldehyde in 0.1 M phosphate buffer (pH 7.4) at 4°C overnight. After rinsing in 0.1 M sodium cacodylate, pH 7.4 containing 7.5% saccharose, cells were post-fixed in 1% OsO_4 solution for 1 h. Ultrathin sections were stained with lead citrate and samples were examined TEM operated at 120 kV. Quantification of ILVs and MVBs was achieved by manually counting vesicles in five images per condition. In addition, exosomes from the cell supernatant of different treatment groups were extracted for experiments.

Western Blot Analysis

RIPA lysis buffer (Beyotime biotechnology, P0013B) was applied to extract the total cells and exosomes protein lysates. The debris were removed by centrifugation at 1,200 g for 10 min at 4 °C. The concentration of total proteins was determined by BCA protein assay kit (Beyotime biotechnology, P0010). The proteins were separated using 10% sodium dodecyl sulfate polyacrylamide gel electrophoresis and subsequently deposited onto PVDF membranes (Millipore, Bedford, MA, USA). The immunological blots were incubated overnight at 4 °C with anti-Alix, anti-CD63, anti-MHCI, anti-MHCII, anti-PD-L1(dilution ratio = 1:1000). The matching secondary antibodies were added to the blots for further visualization. The bolts were visualized under an iBright FL1500 imaging system (Thermo Fisher Scientific Inc., USA) with the presence of enhanced chemiluminescence (Bio-Rad laboratories Co., Ltd., Shanghai, China). The signals

were quantified using g ImageJ software (National Institutes of Health, Bethesda, MD, USA).

Proteomic Analysis of Exosomes Derived from M2 Macrophages

M2 macrophages were treated with vehicle control or R848+C6. Exosomes were then isolated from the cell culture supernatant using an Exosomes Precipitation Kit. For protein digestion, samples were mixed with 4 volumes of 1× PBS, followed by ultrasonic lysis. The lysates were centrifuged at 12,000 g for 10 min at 4°C, and the supernatant was collected. Equal amounts of protein from each sample were taken for digestion. The volume was adjusted to be consistent using lysis buffer, followed by reduction with 5 mM dithiothreitol (DTT) at 56°C for 30 min and alkylation with 11 mM iodoacetamide (IAA) in the dark at room temperature for 15 min. Trypsin was added at a 1:50 (enzyme-to-protein, m/m) ratio for overnight digestion, followed by a second trypsin digestion at a 1:100 ratio for 4 h. Peptides were separated using an EASY-nLC 1200 ultra-high-performance liquid chromatography system. Both the parent ions of the peptides and their secondary fragments were detected and analyzed using the high-resolution Orbitrap. Data were acquired using data-independent acquisition (DIA) mode. Statistical analysis was conducted in Perseus. Log2-transformed abundance values were compared between groups using a student's t-test ($p < 0.05$ considered significant). Hierarchical clustering of significantly altered proteins (>1.5-fold change, $p < 0.05$) was performed based on z-score normalized values. Functional enrichment of Gene Ontology (GO) terms was analyzed using R studio (version 4.4.1).

Isolation and Activation of OT-I T Cells

$CD8^+$ T cells were isolated from spleens of 6-8 week-old OT-I transgenic mice (C57BL/6 background). Briefly, spleens were harvested and mechanically dissociated into a single-cell suspension by grinding through 70 μm cell strainer in complete RPMI-1640 medium. Erythrocytes were lysed using ACK lysing buffer. $CD8^+$ T cells were then purified by negative selection using a mouse $CD8^+$ T cell isolation kit (Miltenyi Biotec). according to the manufacturer's instructions. Cells were stimulated with 2 μg/ml anti-CD3 (BioLegend, 830301) and 2 μg/ml anti-CD28 ((BioLegend, 102102).

Antigen presentation and T cell activation by exosomes

For in vitro antigen presentation assays, M2 macrophages were pretreated with vehicle control, C6, R848, or R848+C6 for 24 hours in the presence of 250 μg/ml endotoxin-free ovalbumin (OVA). Exosomes were isolated from the culture supernatants of the differently treated macrophages. To visualize exosomes-T cell interactions and specific antigen recognition, exosomes collected from antigen-loaded macrophages under various treatments were labeled with PKH67 (green). These labeled exosomes were then co-cultured with Dil (orange)-labeled $CD8^+$ OT-I T cells. After co-culture for 2h, $CD8^+$ OT-I T cells were fixed, permeabilized, stained with anti-OVA peptide-MHC-I (pMHC-I) antibody (red), and the nucleus was stained with DAPI (blue). The co-localization of exosomes (green) and pMHC-I (red) on $CD8^+$ T cells (orange) was visualized using confocal laser scanning microscopy (CLSM, Leica STELLARIS 5, Germany). To evaluate T cell proliferation, OT-I T cells were expanded in complete RPMI-1640 medium supplemented with 50 U/mL mouse IL-2 (PeproTech, # 26156-1) and labeled with 2.5 μM CFSE (Thermo Fisher Scientific, #C34554). These CFSE-labeled T cells (1×10^5 cells/well) were then co-cultured with exosomes from various groups for 5 days. The final concentration of exosomes in the co-culture was 20 μg/mL. Proliferation of antigen-specific OT-I T cells was assessed by FCM based on the percentage of $CFSE^{low}$ cells. T-cell activation: OT-I T cells were co-cultured with exosomes from various groups for 24h. Then the culture supernatants were collected, and the concentrations of IFN-γ and granzyme B were quantified by ELISA to evaluate T-cell activation.

Synthesis and Characterization of $CaCO_3$ Nanoparticles

$CaCO_3$ nanoparticles ($CaCO_3$NPs) were synthesized using a modified gas diffusion method. Briefly, 100 mg of $CaCl_2$ was dissolved in 100 μL of deionized water, followed by dilution with 50 mL of anhydrous ethanol. The mixture was transferred to a glass bottle, which was sealed with parafilm punctured with several needle holes to allow for gas exchange. This bottle, along with two additional vials each containing 1 g of $(NH_4)_2CO_3$, were placed in an incubator at 30 °C for 10 hours to allow ammonium carbonate vapor to diffuse into the $CaCl_2$ solution. After the reaction, the resulting

mixture was centrifuged at 12,000 g for 10 min (Thermo Fisher Scientific, USA) to collect the product. The harvested nanoparticles were washed several times with anhydrous ethanol, redispersed in fresh ethanol, and stored at 4 °C for subsequent use.

Preparation and Characterization of RC6CaCO3L

Hybrid $CaCO_3$ nanoparticles ($CaCO_3$L) were prepared via solvent-diffusion method. In a typical procedure for preparing R848/C6-coloaded NPs (RC6$CaCO_3$L), C6 (2 mg), R848 (0.5 mg), $CaCO_3$ NPs(5 mg), phospholipid (5 mg) (PL, mass ratio of PL:$CaCO_3$ = 1:1), and DSPE-PEG2000 (1 mg) (mass ratio of DSPE-PEG2000:PL = 0.2:1) were co-dispersed in a 2 mL mixture of ethanol and $CHCl_3$(1:1, v/v). The mixture was stirred overnight at room temperature, followed by probe sonication (300 W, 2 s pulse on/3 s pulse off for 30 cycles). The organic solvents were then removed by rotary evaporation, and the resulting thin film was vacuum-dried. The film was subsequently hydrated by dissolving it in anhydrous ethanol, which was then injected uniformly into 18 mL of deionized water under stirring. After stirring for 10 minutes at room temperature, the solution was centrifuged at 3,000 g for 10 min to remove large aggregates. The final supernatant, containing the hybrid nanoparticles, was collected and stored at 4 °C for further use. To generate control nanoparticles, the following formulations were synthesized using the same protocol by omitting specific components from the initial mixture: (a) C6$CaCO_3$L: R848 was omitted. (b) R$CaCO_3$L: C6 was omitted. (c) $CaCO_3$L: Both C6 and R848 were omitted.

Preparation of Cy5.5-labeled nanoparticles for *in vivo* distribution. For *in vivo* distribution assessment, Cy5.5-labeled $CaCO_3$L were prepared for visualization using an IVIS Lumina III optical imaging system. The procedure was identical to that described above, except that Cy5.5 (1 mg), $CaCO_3$ NPs (5 mg), PL (5 mg), and DSPE-PEG2000 (1 mg) were dispersed in the ethanol/chloroform mixture, omitting C6 and R848.

Characterization of RC6$CaCO_3$L: The size of RC6$CaCO_3$L nanoparticles was analyzed by a Malvern mastersizer 2000 (Malvern, UK). The morphology of RC6$CaCO_3$L was observed by TEM (Hitachi HT7800). To quantify the encapsulated drugs, the nanoparticles suspension was lysed to disrupt its structure. The solution was then

centrifuged at 10,000 g for 10 min, and the clear supernatant was collected for HPLC analysis. C6 was quantified using the ELSD. The concentration of each drug was calculated from its corresponding calibration curve constructed with standard solutions of known concentration. The EE was calculated according to the following equations: EE (%) = (Weight of encapsulated drug / Weight of total drug input) × 100%.

Preparation and Characterization of RC6CaAlgMS

Monodisperse calcium-crosslinked alginate microspheres containing the hybrid nanoparticles loaded with C6 and R848 (RC6CaL), denoted as RC6CaAlgMS were prepared using a simplified microfluidic device. The microfluidic device was homemade according to a procedure described in our previous study. The aqueous phase containing 1 wt% alginate and 0.2 wt% of RC6CaL was used as the dispersed phase. The continuous phase consisted of liquid paraffin with 0.15 wt% CaI_2 and 5 wt% Span 80. The dispersed phase and continuous phase were loaded into separate syringes and pumped into the microchannel. The flow rate of the dispersed phase was maintained constant at 1 μL/min, while the flow rate of the continuous phase was varied (100, 200, 300, 400, 500, and 600 μL/min) to investigate its effect on droplet formation. The monodisperse water-in-oil (W/O) droplets were collected in a beaker containing 20 mL of continuous phase, where they underwent a crosslinking reaction for 48 h at ambient temperature. Afterwards, the solidified microspheres were repeated washing with 5 wt% of hexane and 95 wt% of isopropanol to remove any remaining oil and surplus cross-linker. Finally, the as-prepared RC6CaAlgMS were air-dried and stored at room temperature prior to use. Under the condition of a 1:400 ratio of dispersed phase to flowing phase, CaAlgMS, RCaAlgMS, C6CaAlgMS, Cy5.5-CaAlgMS were prepared by $CaCO_3$L, R$CaCO_3$L, C6$CaCO_3$L, and Cy5.5-labeled $CaCO_3$L, respectively, and AlgMS was not loaded with nanoparticles.

Characterization of microspheres. The diameter of the microspheres produced under each condition were analyzed using optical microscopy images. The coefficient of variation (CV) of microspheres was calculated by the following formula: $CV=\sigma/d_{av}\times100\%$, where σ represents the standard deviation of the particle diameter, and d_{av} is the average particle diameter of the microspheres (n=100). The determination

of microsphere average size was done with Image J software, and size distribution was analyzed using Origin software. The microspheres' morphology was analyzed with a scanning electron microscope (SEM, Phenom Pharos, Phenom Scientific, Netherlands). The chemical compositions of RC6CaAlgMS were analyzed using SEM along with EDS.

***In vitro* acidity neutralization ability of CaNPsAlgMS**

The acidity neutralization capacity of CaAlgMS and RC6CaAlgMS were evaluated by measuring the change in pH of an acidic buffer solution following the introduction of CaAlgMS and RC6CaAlgMS, with AlgMS serving as the control group. Briefly, 50 mg of microspheres were added to 10 mL of PBS solution (pH=6.5), followed by gently mixing under a magnetic stirrer. At a designated time point, the pH value of microsphere suspensions was measured by using a digital pH meter (Mettler-Toledo Instruments Co., Ltd., Shanghai, China).

***In vitro* drug release behavior**

The pH-responsive release profiles of R848 and C6 from the RC6CaAlgMS nanoparticles were investigated using a dialysis method under conditions simulating the physiological (pH 7.4) and tumor microenvironment (pH 6.5). Briefly, 50 mg of the RC6CaAlgMS suspension was placed in a dialysis bag (Molecular Weight Cut-Off: 3.5 kDa). The bag was then immersed in 20 mL of PBS, pre-adjusted to either pH 7.4 or 6.5. The entire system was incubated in a shaking water bath at 37 °C with a constant agitation speed of 100 rpm. At 0, 2, 4, 6, 8, 12, 24, 36, 48, and 72 hours, 1 mL of the external release medium was withdrawn and replaced with an equal volume of fresh, pre-warmed medium to maintain a constant volume, The concentrations of R848 and C6 were quantified.

Macrophage polarization capacity of RC6CaAlgMS *in Vitro*

To evaluate the effects of different treatments on TAM repolarization, BMDMs were first polarized into a TAM-like phenotype by culturing them for 24 hours in a medium consisting of 40% conditioned medium from Hepa1-6 tumor cells and 60% DMEM medium supplemented with 50 ng/mL MCSF. These induced TAMs were then treated with PBS, AlgCaMS, RCaAlgMS, C6CaAlgMS, or RC6CaAlgMS for 24 hours. For

FCM analysis, treated cells were harvested and stained with fluorescently labeled antibodies against mouse FITC-F4/80 (dilution ratio = 1:200), PE-CD80 (dilution ratio = 1:200), and APC-CD206 (dilution ratio = 1:200). The populations of M1-type TAMs ($F4/80^+CD80^+$) and M2-type TAMs ($F4/80^+CD206^+$) were quantified using a flow cytometer (BD FACSCanto, BD Biosciences, USA), and the data were analyzed with FlowJo software (version, V10.8.1).

Immunofluorescence staining was performed to evaluate the expression of intracellular markers. After being fixed by 4% paraformaldehyde for 20 min, permeabilized in 0.3% Triton-X for 10 min and blocked with 10% bovine serum albumin (BSA) solution for 2 h, the cells were treated with iNOS (Abcam, USA, dilution ratio = 1:500) and Arg-1 (Abcam, USA, dilution ratio = 1:200) primary antibody. Thereafter, the as-treated cells were incubated with Alexa Fluor 594 and 488 conjugated secondary antibody (Abcam, USA, dilution ratio = 1:500) for iNOS and Arg-1, respectively. Subsequently, the specimens were stained by DAPI (Sigma, USA) for cell nuclei before the observation using a CLSM (Leica STELLARIS 5, Germany).

The concentrations of TNF-α and IL-12 in cell culture supernatants were quantified using commercial ELISA kits, following the manufacturer's protocols. Briefly, following the indicated treatments, supernatant was collected by centrifugation at 1,000 g for 3 min. The supernatant was then incubated with pre-coated capture antibodies, followed by multiple washing steps and incubation with an enzyme-conjugated detection antibody. Absorbance was measured using a microplate reader (BioTek, Winooski, VT, USA), and cytokine concentrations were determined based on a standard curve.

Phagocytosis assay *in Vitro*

To quantitatively assess the phagocytic capacity of TAMs after 24 hours of treatment, we employed FCM and fluorescence microscopy assays. TAMs and Hepa1-6 cells were differentially labeled with CellTracker Green CMFDA (Beyotime, C2231) and CellTracker Deep Red (Thermo Fisher, A66433), respectively. Labeled TAMs (4×10^5) were then co-cultured with Hepa1-6 cells at an effector-to-target (E:T) ratio of 1:2 for 2 hours. Subsequently, non-internalized tumor cells were thoroughly removed by gentle

washing, and the remaining cells were harvested to generate a single-cell suspension. For flow cytometric analysis, phagocytosis was quantified by FCM (BD Accuri C6), gating on the TAM population and determining the percentage of double-positive ($Green^{+}Deep\ Red^{+}$) cells, which represent TAMs that have engulfed tumor cells. For spatial visualization and confirmation, Phagocytic activity was quantified by enumerating $Green^{+}Deep\ Red^{+}$ TAMs across ten random fields per replicate using fluorescence microscopy (Leica DMi8, Germany).

Cytotoxic Activity of $CD8^{+}$ T Cells against Tumor Cells *in Vitro*

Preparation of antigen-pulsed TAMs and exosomes isolation. Whole-cell lysates were prepared from Hepa1-6 cells through three repeated freeze-thaw cycles in liquid nitrogen, followed by sonication on ice (40% amplitude, 5 sec pulse-on, 5 sec pulse-off, total 2 min) using an ultrasonicator (SCIENTZ-IID, China). The protein concentration was determined using a BCA protein assay kit (Beyotime Biotechnology, China). TAMs were then treated for 24 hours with PBS (control), CaAlgMS, RCaAlgMS, C6CaAlgMS, or RC6CaAlgMS in the presence of Hepa1-6 lysates (50 μg/mL total protein). Exosomes were subsequently isolated from the conditioned supernatants using the Exosomes Precipitation Kit (Solarbio, EX0016). To investigate T-cell priming and proliferation. Total $CD3^{+}$ T cells were isolated from the spleens of 6-8 week-old syngeneic C57BL/6 mice using the MojoSort™ Mouse CD3 T Cell Isolation Kit (BioLegend). The isolated T cells (1×10^{6} cells/mL) were co-cultured for 24 hours in RPMI-1640 complete medium (Gibco) with exosomes (20 μg/mL) derived from the respective TAM treatment groups. The percentages of $CD4^{+}$ (FITC anti-mouse CD4, BioLegend, 100412) and $CD8^{+}$ T cells (PE anti-mouse CD8a, BioLegend, 100706), as well as intracellular IFN-γ (APC anti-mouse IFN-γ, BioLegend, 505808) expression in $CD8^{+}$ T cells, were analyzed by FCM (BD FACSCanto, BD Biosciences, USA). The cytotoxic activity of $CD8^{+}$ T cells was evaluated against live Hepa1-6 cells. Target cells were seeded in 12-well plates at a density of 1×10^{5} cells per well and allowed to adhere overnight. $CD8^{+}$ T cells were added at an effector-to-target (E:T) ratio of 1:1, together with exosomes (20 μg/mL) from differentially treated TAMs. A control group of Hepa1-6 cells cultured without T cells was included. Following 24

hours of co-culture, all cells were harvested and subjected to apoptosis analysis using an Annexin V-FITC kit (Beyotime Biotechnology) by staining for 30 min at room temperature in the dark. The apoptotic cells were analyzed by FCM (BD FACSCanto, USA).

Fluorescence Imaging and Biodistribution Study *In Vivo*

When the tumor volumes reached 200 mm^3, the mice were randomly assigned to three groups (n = 3 per group). The following formulations were administered via peritumoral injection in a total volume of 50 μL and a total mass of 10mg, (I) free Cy5.5 dye (1 mg/kg, Lumiprobe, #41020), (II) Cy5.5-labeled AlgMS microspheres (equivalent to 1 mg/kg Cy5.5), or (III) Cy5.5-labeled CaAlgMS microspheres (equivalent to 1 mg/kg Cy5.5). *In vivo* and *ex vivo* fluorescence imaging. Whole-body fluorescence imaging was performed at predetermined time points (0, 1, 2, 3, 5, 7, and 9 days post-injection) using an IVIS SpectrumCT *in vivo* imaging system (PerkinElmer, USA). The fluorescence intensity (expressed as radiant efficiency in [$p/s/cm^2/sr$] / [$\mu W/cm^2$]) at the injection site was quantified using Living Image software (version 4.7.2, PerkinElmer). On day 9 post-injection, the mice were euthanized by CO_2 asphyxiation. The tumors and major organs (heart, liver, spleen, lungs, and kidneys) were harvested, rinsed with PBS, and immediately subjected to *ex vivo* imaging using the same system and parameters. The fluorescence signal of each organ was quantified to assess the final biodistribution and clearance profile of the different formulations.

Quantitative Assessment of Intratumoral pH

The efficacy of the microspheres in ameliorating tumor acidosis was quantitatively assessed in a Hepa1-6 subcutaneous tumor model using the pH-sensitive ratiometric fluorescent probe, SNARF®-1 AM (Invitrogen, Thermo Fisher Scientific, #S22803). Female C57BL/6 mice (6-8 weeks old) were subcutaneously inoculated with Hepa1-6 cells (2×10^6 cells per mouse) into the right flank. When tumor volumes reached approximately 200 mm^3, the mice were randomly assigned to six treatment groups (n = 3 per group). The groups received a single peritumoral injection of the following formulations: Group I: PBS, Group II: CaAlgMS microspheres, Group III: DCaAlgMS, Group IV: DRCaAlgMS, Group V: DC6CaAlgMS, Group VI: DRC6CaAlgMS. The

following formulations were administered via peritumoral injection in a total volume of 50 μL and a total mass of 10mg. At 24 hours post-injection of the microspheres, each mouse was intravenously injected with 1 nmol of the pH-sensitive dye SNARF-1 in a volume of 100 μL. After a 20 minutes interval to allow for probe distribution and intracellular pH equilibration, the mice were euthanized via CO_2 asphyxiation. Tumors were immediately excised. Fluorescence imaging and pH calculation. The tumor tissues were then imaged using an IVIS Lumina III *in vivo* imaging system (PerkinElmer) with the following filter sets: excitation = 548 nm, emission = 580 nm and 640 nm. The fluorescence intensity ratio (I_{640} / I_{580}) was calculated for each tumor section using Living Image software (v4.7.2, PerkinElmer). The intratumoral pH values were determined by interpolating these ratios against a standard calibration curve generated according to the manufacturer's protocol using pH-standard buffers.

Antitumor Efficacy and Survival Analysis of DRC6CaAlgMS *In Vivo*

The antitumor efficacy of the microspheres was evaluated in a subcutaneous luciferase-expressing Hepa1-6 (Luc-Hepa1-6) tumor model established in C57BL/6 mice by inoculating 2 × 10^6 cells into the right flank. When tumor volumes reached approximately 60-80 mm^3, the mice were randomly assigned to six treatment groups (n = 5): G1: Control (PBS), G2: CaAlgMS, G3: DCaAlgMS, G4: DRCaAlgMS, G5: DC6CaAlgMS, or G6: DRC6CaAlgMS. All formulations were administered via intratumoral injection in a total volume of 50 μL and a total mass of 10mg per mouse. The injections were performed once on day 0. Tumor growth was monitored every three days using digital calipers, with volume calculated as (length × $width^2$)/2. Tumor viability was assessed via *in vivo* bioluminescence imaging at days 0, 7, and 14 post-treatments. Mice were intraperitoneally injected with D-luciferin (150 mg/kg) and imaged 10-15 minutes later using an IVIS Spectrum system, with signal intensity quantified to determine tumor burden. For histological and immunohistochemical analysis. On day 14 post-treatment, mice were euthanized. Primary tumors and draining lymph nodes were harvested. Tissues were fixed in 4% paraformaldehyde for 24 hours at 4°C, and then processed for histology. For histological analysis, sections were subjected to standard H&E staining to evaluate general morphology and necrotic areas.

For the safety assessment, mice were euthanized at the end of follow-up. The main organs (heart, liver, spleen, lung, and kidney) were collected and cut into slices. The slices were subjected to H&E analysis to determine the histological damages under a light microscope (Leica DMi8, Germany). For proliferation and apoptosis assessment, deparaffinized tumor tissue sections were processed for Ki67 IHC staining and TUNEL assay, respectively.

For survival analysis, a separate cohort of mice (n = 5 per group) received the same treatments and was monitored for 60 days. Survival rates were recorded, with the endpoint defined as tumor volume exceeding 1500 mm^3, ulceration, or significant body weight loss (>20%), in accordance with institutional animal ethics guidelines. The survival time of each mouse was recorded, and Kaplan-Meier survival curves were plotted and analyzed using the log-rank test in GraphPad Prism (version 8.0.0).

Isolation and Protein Analysis of Plasma Exosomes

Plasma samples were collected from tumor-bearing mice orbital blood on day 14 post-treatment and were subsequently processed for exosomes isolation using the ExoQuick ULTRA EV Isolation Kit (SBI System Biosciences). Briefly, 250 µL of clarified plasma was combined with 67 µL of ExoQuick reagent. After a 30 min incubation on ice and centrifugation at 3000 g for 10 min, the pelleted exosomes were resuspended and further purified through a provided chromatography column, following the kit's instructions. The hydrodynamic diameter of the isolated exosomes was determined by DLS using a Malvern mastersizer 2000 (Malvern, UK). The morphology of exosomes was assessed by TEM operated at 120 kV. Exosomal protein extraction and quantification. The purified exosomes suspension was mixed with an equal volume of RIPA lysis buffer (Beyotime biotechnology, P0013B). Protein concentration was quantified via a BCA assay, and equal amounts of protein were subjected to SDS-PAGE and Western blotting to detect specific markers (CD63, Alix) and proteins of interest (MHC-I, MHC-II, PD-L1).

Immune profiling evaluation of TME and Systemic Response.

A comprehensive immune profiling analysis was conducted to delineate the immunomodulatory effects of the treatments. At 7 days post-treatment, TDLNs were

harvested from euthanized mice. The tissues were mechanically dissociated and enzymatically digested into single-cell suspensions. For surface staining, cells were incubated with an Fc receptor block and then stained with a cocktail of fluorescently conjugated antibodies for 30 min at 4°C in the dark. The antibody panel included: anti-CD45, anti-CD3, anti-CD4, anti-CD8a, anti-NK1.1, anti-F4/80, anti-CD86, anti-CD206, anti-CD11c, anti-CD80, and anti-MHC-II (all used at 1:200 dilution). For IFN-γ and Foxp3 intracellular staining, cells were fixed, and permeabilized prior to staining with anti-IFN-γ (1:100) or anti-Foxp3 (1:100). Cells were analyzed by FCM (BD FACSCanto, USA), and the data were analyzed using Flow Jo software. Serum concentrations of IFN-γ and TNF-α were quantified using commercial ELISA kits.

Tumor tissues were harvested from mice 7 days post-treatment, then fixed, dehydrated, and paraffin-embedded. Following sectioning, deparaffinization, and antigen retrieval, the sections were blocked with 5% BSA for 30 min at room temperature and subsequently incubated overnight at 4°C with the following primary antibodies. Anti-F4/80 (1:200) with anti-CD86 (1:200) to identify M1 macrophages, anti-F4/80 with anti-CD206 (1:200) to identify M2 macrophages, and anti-CD4 (1:200) or anti-CD8 (1:200) to evaluate T cell subsets. After washing, sections were incubated with species-appropriate Alexa Fluor-conjugated secondary antibodies for 1 h at room temperature in the dark, followed by nuclear staining with DAPI. Images were captured using a CLSM (Leica DMi8, Germany), and fluorescence intensity and positive cell counts were quantified using ImageJ software.

Evaluating the Contribution of Macrophages and $CD8^+$ T Cells to DRC6CaAlgMS Efficacy

To establish the subcutaneous tumor model, (Luc-Hepa1-6) tumor model established in C57BL/6 mice. Mice were randomly assigned to the following experimental groups upon reaching a tumor volume of approximately 60-80 mm^3: (a) DRC6CaAlgMS+Isotype control antibody; (b) DRC6CaAlgMS+anti-CD8α antibody; (c) DRC6CaAlgMS+PBS-liposome control; (d) DRC6CaAlgMS+Clodronate liposomes. For the depletion of $CD8^+$ T cells, mice were administered intraperitoneal (i.p.) injections of an anti-mouse CD8α antibody (Bio X Cell) at a dose of 200 μg/mouse

every 4 days throughout the entire tumor monitoring period. An isotype control antibody (Bio X Cell) was administered on the same schedule to the control group. Macrophages were depleted using clodronate liposomes. Mice received intravenous (i.v.) injections of 200 μL of clodronate liposomes (Yesen, China) one day before treatment and every five days thereafter. Control mice received equivalent injections of PBS-loaded liposomes. Tumor dimensions were measured every three days using a digital caliper, and volumes were calculated using the formula: Volume = (length × width^2) / 2. Tumor viability was assessed via *in vivo* bioluminescence imaging at days 0, 7, and 14 post-treatments using an IVIS Spectrum system, with signal intensity quantified to determine tumor burden.

Antitumor Effects of the Combination of DRC6CaAlgMS and αPD-L1 Therapy

To investigate whether DRC6CaAlgMS treatment could elicit a potent systemic antitumor immune response capable of controlling untreated distant lesions, a bilateral Hepa1-6 tumor model was established in 6-8 week old female C57BL/6 mice. The primary tumor was initiated on Day -7 by subcutaneous injection of 2 × 10^6 Hepa1-6 cells (suspended in 100 μL PBS) into the right dorsal flank. A distant tumor was subsequently established on the left dorsal flank on Day -3 by injecting an identical number of cells. On Day 0, when the primary tumor volumes reached approximately 60-80 mm^3, mice were randomly assigned to four treatment groups (n=5): (a) PBS, (b) αPD-L1 monotherapy, (c) DRC6CaAlgMS monotherapy, and (d) DRC6CaAlgMS+αPD-L1 combination therapy. Mice in groups 3 and 4 received intratumoral injections of DRC6CaAlgMS (10 mg/mouse in 50 μL) specifically into the primary tumor on Days 0. Concurrently, mice in groups 2 and 4 received intravenous injections of anti-PD-L1 antibody (αPD-L1, Bio X Cell, 2 mg/kg) via the tail vein every three days for a total of three doses, starting on Day 0. The growth of both the treated primary tumor and the untreated distant tumor was meticulously monitored every 3 days by measuring the length (L) and width (W) with a digital caliper; tumor volume was calculated using the formula $V = (L \times W^2) / 2$. Animal body weight was recorded concurrently as an indicator of treatment-related toxicity. Survival was tracked for 60 days post-treatment initiation, with the humane endpoint defined as a tumor volume

exceeding 1500 mm^3 or the presentation of any signs of distress. The immune responses were systematically analyzed by FCM and ELISA as aforementioned.

Evaluation of Embolic Efficacy Using *Ex Vivo* and *In Vivo* Models

The embolic performance and biocompatibility of the microspheres were rigorously evaluated through a combination of *ex vivo* and *in vivo* models. For initial *ex vivo* assessment, liver scaffolds were prepared from male Sprague-Dawley rats (8-10 weeks). Following euthanasia by CO_2 asphyxiation, livers were harvested via midline laparotomy. The portal vein, inferior vena cava, and bile duct were cannulated (using 21-, 22-, and 23-gauge needles, respectively), flushed with PBS , and stored at -80 °C. Decellularization was achieved by sequential perfusion via the portal vein and bile duct with distilled water (30 min), 4% (v/v) Triton X-100 containing 0.02% EGTA (3 h), and 0.5% (w/v) sodium dodecyl sulfate (SDS, 3 h) at a constant flow rate of 7 $mL\cdot min^{-1}$, followed by a 20-minute perfusion with 0.1% peracetic acid for disinfection and a final rinse with deionized water. The resulting acellular scaffolds were then used to assess the injectability and distribution of DRC6CaAlgMS, which were infused via the portal vein cannulation and visualized under a stereomicroscope.

For *in vivo* functional assessment, an established rabbit renal artery embolization model was employed. New Zealand white rabbits (2.5-3.0 kg) were anesthetized via intravenous injection of sodium pentobarbital (30 mg/kg) into the marginal ear vein. The right femoral artery was surgically exposed, and a 4-Fr vascular sheath (Cook, USA) was inserted. Under real-time digital subtraction angiography (DSA; AlluraXper, Philips, Netherlands) guidance, a 2.7-Fr microcatheter (Terumo, Japan) was advanced to selectively catheterize the target renal artery. Baseline renal arteriography was performed by injecting iodixanol contrast medium (Visipaque™, 320 mg I/mL) to delineate the renal vasculature. A homogeneous suspension of CaAlgMS in iodixanol was then slowly infused under fluoroscopic monitoring until complete angiographic stasis was achieved. Post-embolization angiography was immediately conducted to confirm the cessation of blood flow. The durability of embolization was assessed via follow-up computed tomography angiography (CTA) at 2 weeks post-procedure. At this endpoint, rabbits were euthanized, and the embolized kidney, contralateral kidney, heart,

liver, spleen, and lungs were harvested. Macroscopic examination of the kidneys revealed a marked reduction in size and extensive pallor in the embolized kidney compared to the normal, well-perfused contralateral kidney, indicating successful infarction. All tissues were fixed in 4% neutral buffered formalin for 48 hours, processed for paraffin embedding, and sectioned. Histopathological evaluation of tissue morphology and infarction extent was performed on H&E-stained sections.

***In Vivo* TACE Therapeutic Efficacy**

The therapeutic efficacy of the developed formulation was evaluated in an orthotopic N1S1 rat hepatocellular carcinoma model. Seven days post-tumor inoculation, baseline tumor volume was quantified using T2-weighted MRI on a 3.0-T scanner (Philips Ingenia, Netherlands). Rats bearing tumors of approximately 200 mm^3 were randomly allocated into four experimental groups (n = 3 per group): (a) Control; (b) αPD-L1 monotherapy (2 mg/kg, i.v.); (c) DRC6CaAlgMS embolization; (d) DRC6CaAlgMS+αPD-L1. All formulations were administered via TACE in a total mass of 25mg per rat. For the TACE procedure, a laparotomy was performed to expose the gastroduodenal artery. After ligation of the distal segment, a microcatheter was retrogradely inserted into the proximal artery, with successful placement confirmed by the observation of a pulsatile blood column. The proximal artery was then ligated, and the catheter was carefully withdrawn. To minimize embolic agent reflux, the common hepatic artery was temporarily occluded during the infusion. Rats in the treatment groups received a superselective intra-arterial injection of 200 μL of the respective embolic suspension, while control animals underwent sham surgery without infusion. Tumor growth was monitored via serial MRI at days 0, 7, and 14. On day 14, all rats were euthanized, and tumor tissues were harvested for subsequent histopathological analysis. H&E staining was performed on tissue sections to comprehensively assess treatment-induced alterations in tumor morphology, necrotic area, and overall tissue architecture. In addition, seven days post-treatment, tumor tissues were harvested from the rats for immunofluorescence analysis. Tissue sections were stained to detect $F4/80^+CD206^+$ M2 macrophages, $CD3^+CD8^+$ T cells, and $CD3^+CD4^+$ T cells, following the protocol described earlier. Images were acquired using a CLSM (Leica DMi8,

Germany). Subsequent quantification of fluorescence intensity and the enumeration of positively stained cells were performed using ImageJ software.

Statistical analysis

Statistical analyses were conducted using GraphPad Prism 8.0. Data are expressed as the mean ± SD from at least three independent experiments, each performed with triplicate technical replicates, unless stated otherwise. No data points were excluded. As specified in the figures, comparisons were made using unpaired two-tailed Student's t-test, one-way or two-way ANOVA. Survival curves were generated by the Kaplan-Meier method, and differences were assessed with the log-rank test.

SUPPLEMENTARY FIGURES AND FIGURE LEGENDS

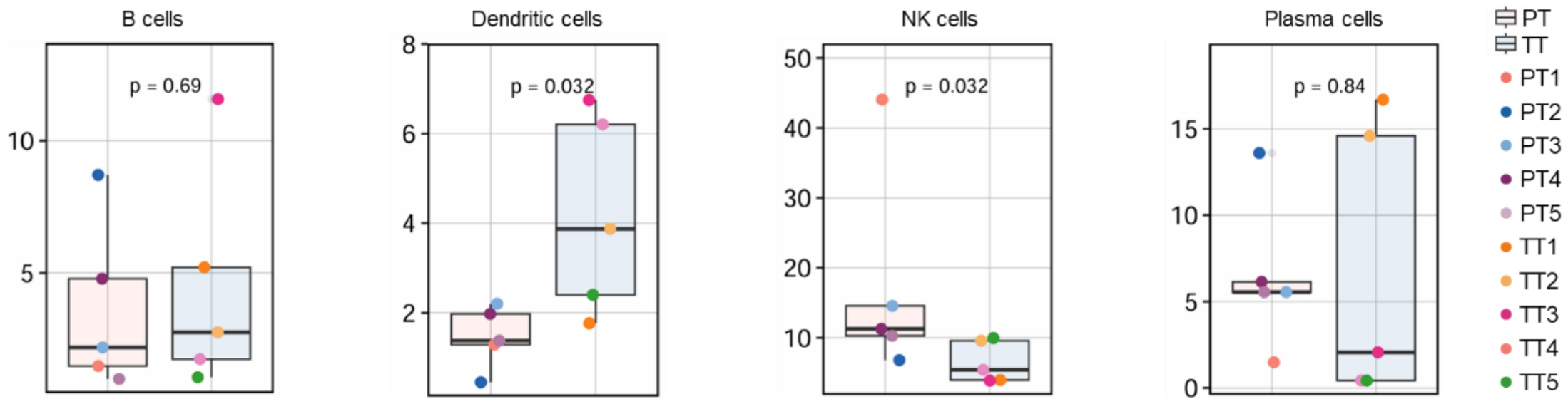

Fig. S1. Box plots showing the relative proportions of immune cell clusters across the PT and TT groups in the discovery cohort (n = 5 per group). PT, primary tumor; TT, TACE tumor.

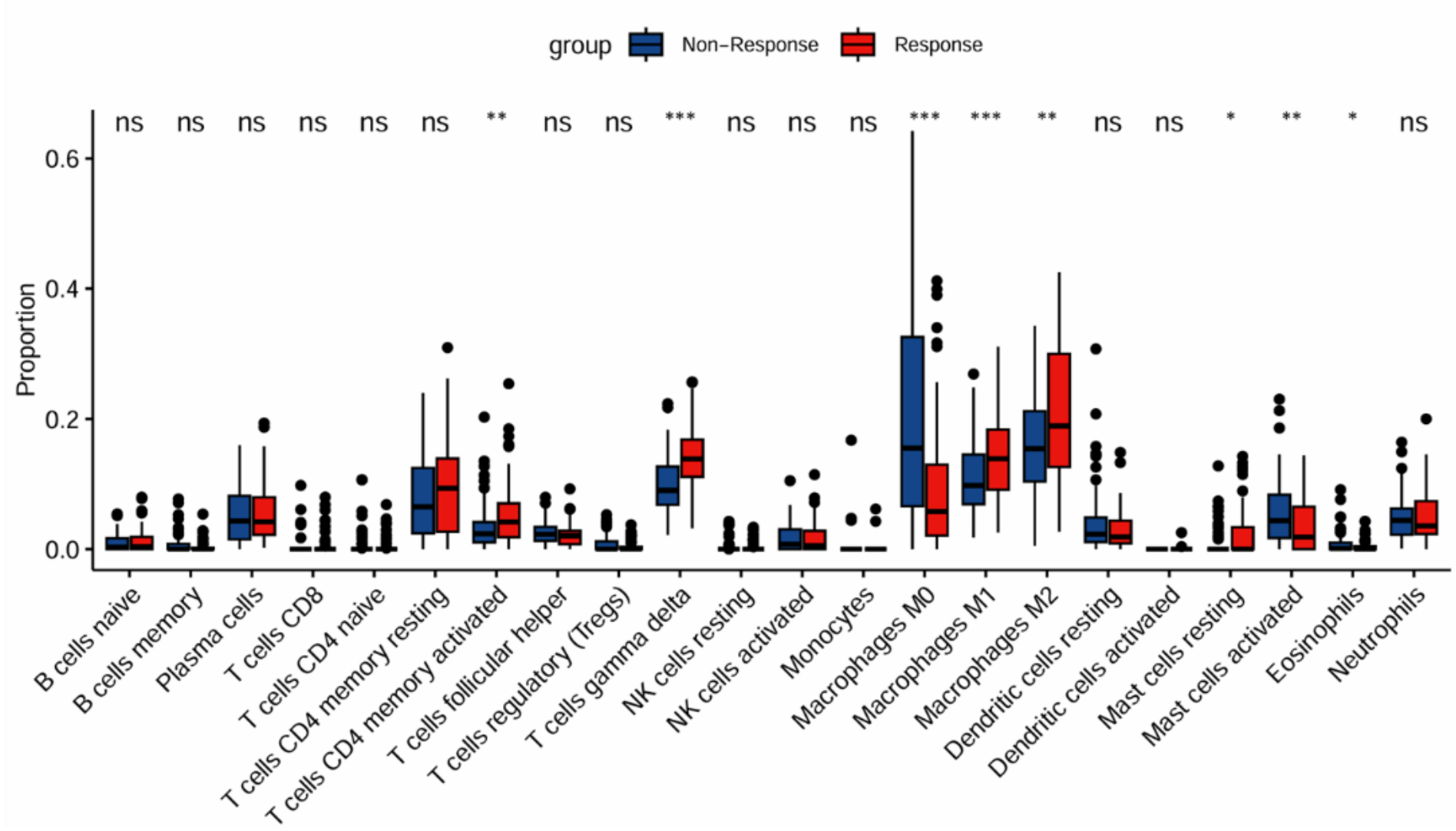

Fig. S2. CIBERSORT-based immune cell profiling in tumor tissues from TACE responders and non-responders. Box plots illustrate the estimated proportions of 22 tumor-infiltrating immune cell subsets in TACE responders (red) and non-responders (blue) analyzed using the CIBERSORT algorithm. The statistical analysis was performed with ANOVA. P > 0.05 indicated no significance between two groups. *P < 0.05, **P < 0.01, ***P < 0.001.

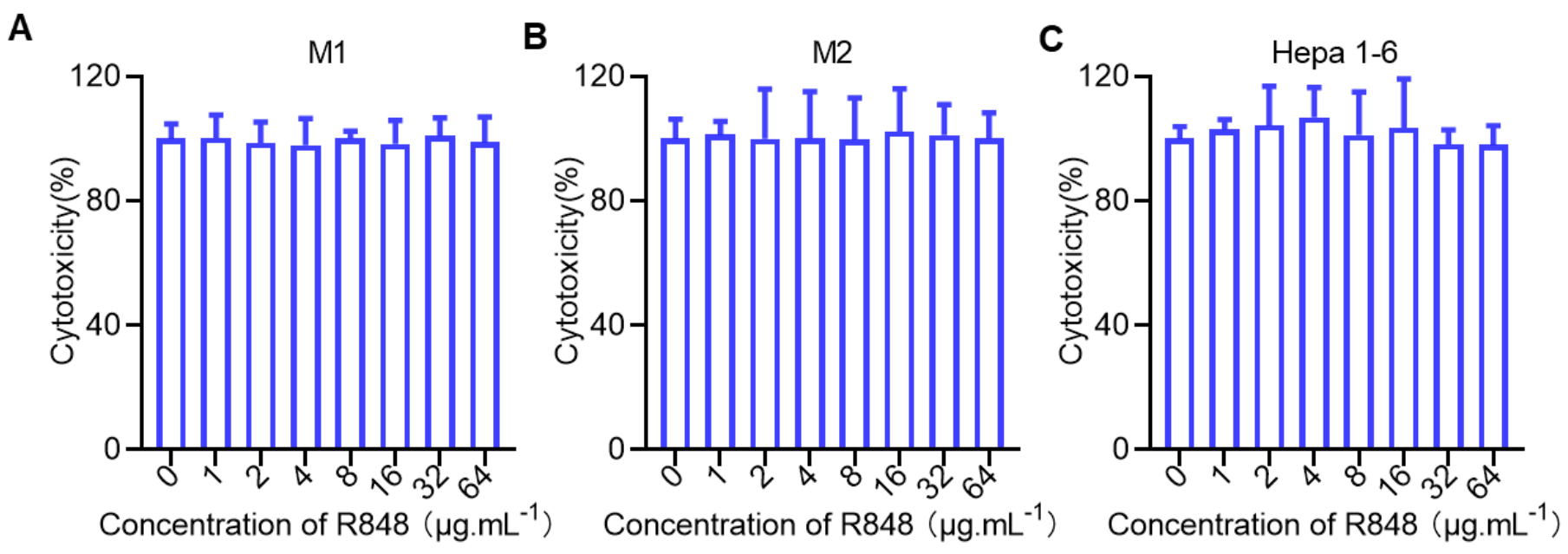

Fig. S3. Cell viability of M1 macrophages(A), M2 macrophages(B), and Hepa1-6 tumor cells(C) after incubation with different concentrations of free R848 for 24 h under normal.

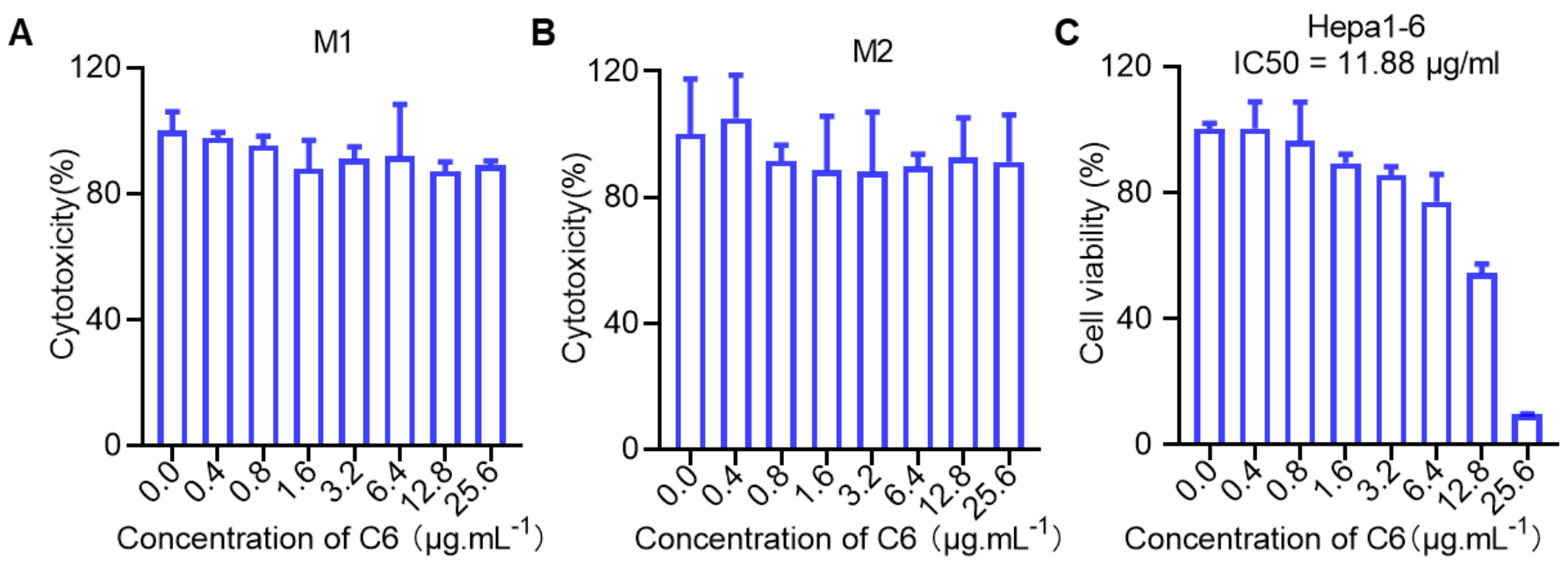

Fig. S4. Cell viability of M1 macrophages(A), M2 macrophages(B), and Hepa1-6 tumor cells(C) after incubation with different concentrations of free C6 for 24 h under normal condition.

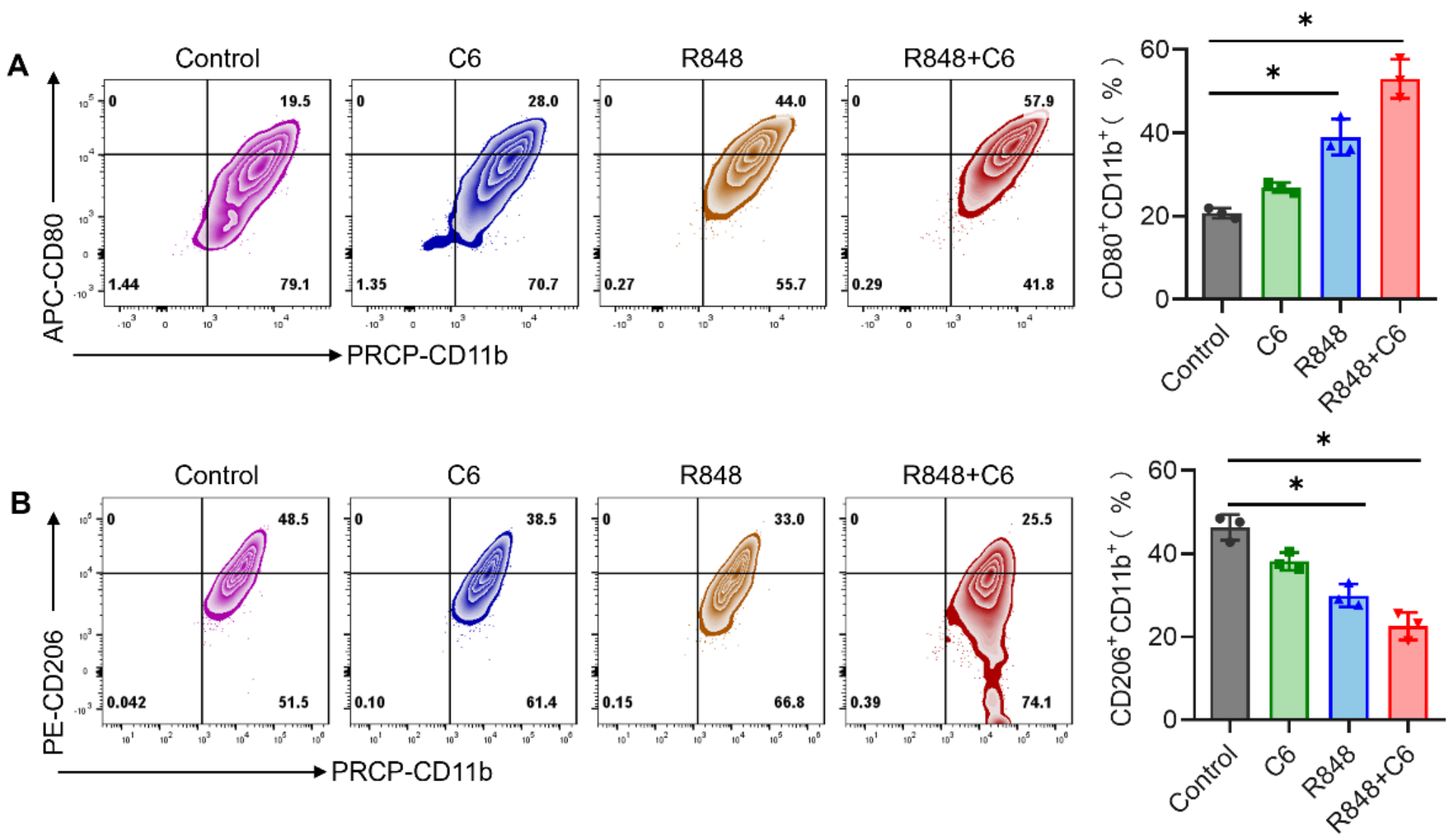

Fig. S5. Flow cytometry analysis and corresponding quantification of (A) M1 macrophages ($CD86^+$) and (B) M2 macrophages ($CD206^+$) with different treatments. The statistical analysis was performed with ANOVA. $P > 0.05$ indicated no significance between two groups. $^*P < 0.05$.

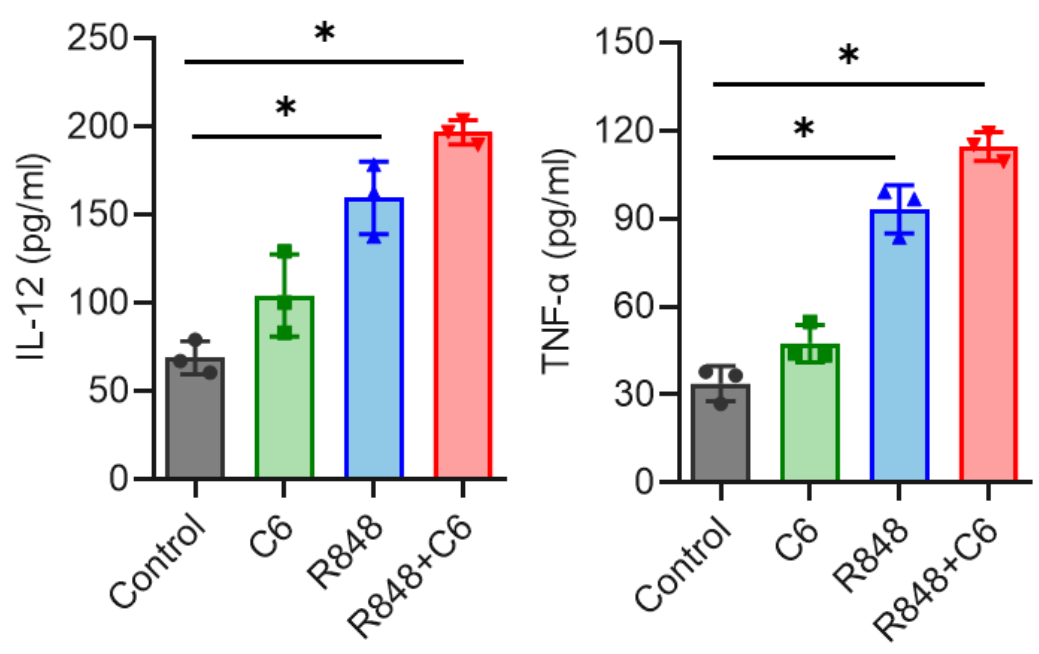

Fig. S6. IL-12 and TNF-α measurement in culture supernatants of differently treated TAMs. The statistical analysis was performed with ANOVA. P > 0.05 indicated no significance between two groups. *P < 0.05.

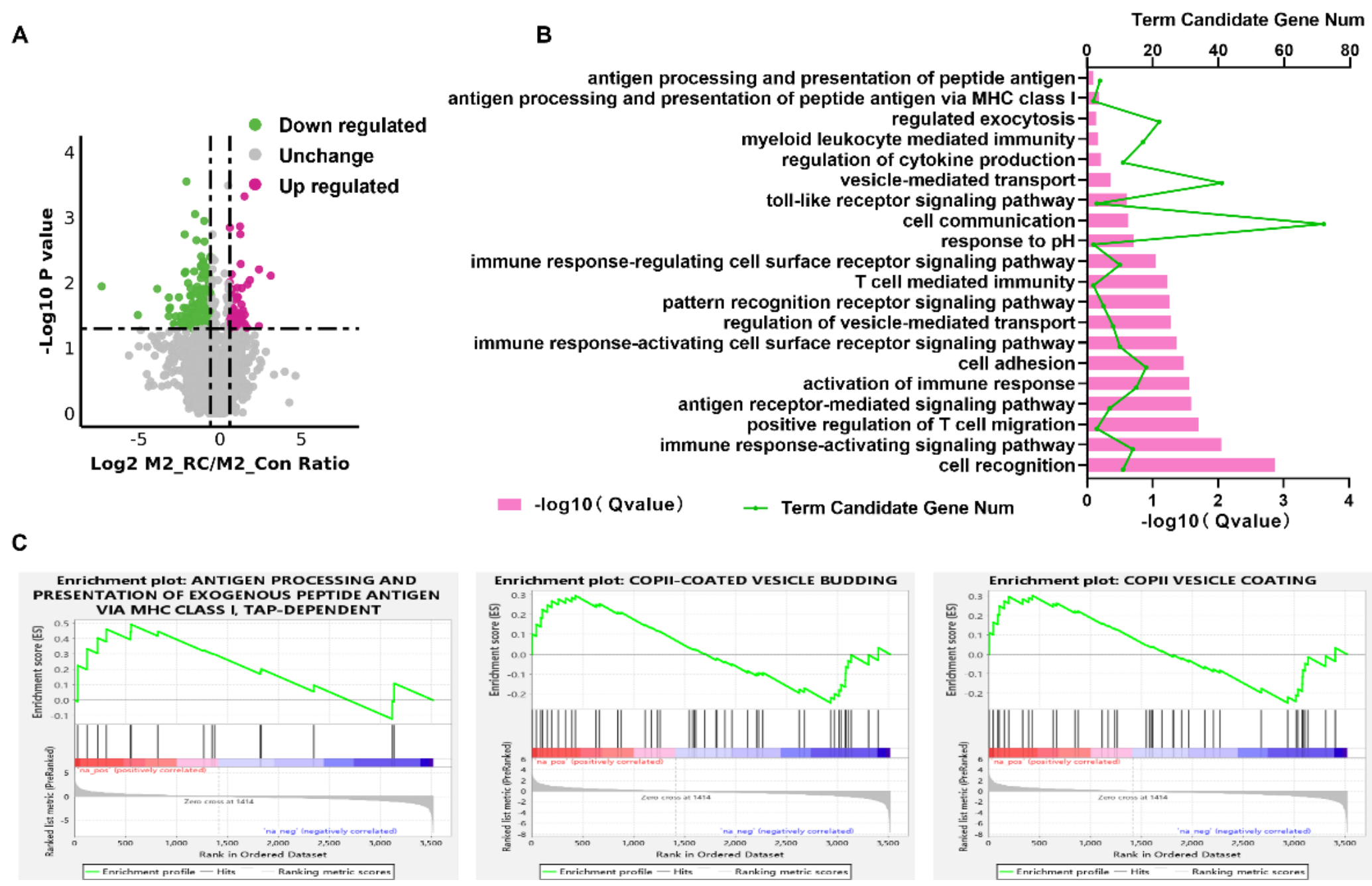

Fig. S7. Proteomic profiling of macrophage treated by R848+C6(M2-RC) and vehicle(M2-Con) (A) Volcano plot delineates the differential protein expression between M2-RC and M2-Con. (B) GO enrichment analysis of biological processes reveals significantly enriched pathways associated with proteins upregulated in M2RC macrophages. The enrichment pattern indicates a functional shift in M2RC macrophages. (C) GSEA enrichment plot of antigen processing and presentation of exogenous peptide antigen via MHC class I TAP-dependent pathway, COPI-coated vesicle budding, and COPII vesicle coating.

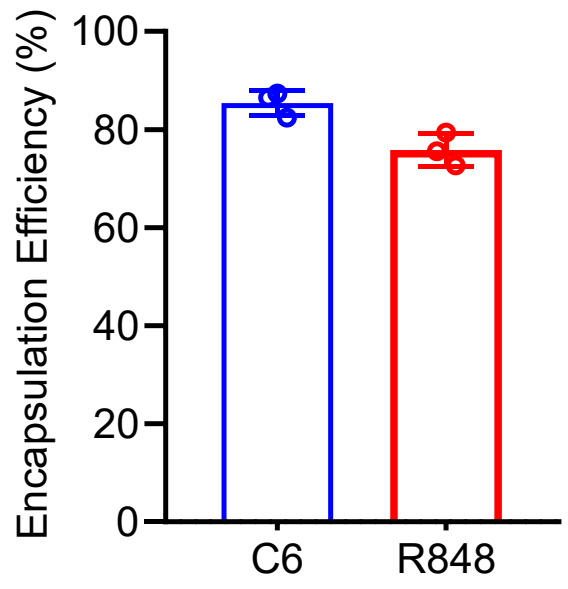

Fig. S8. The encapsulation efficiency (EE) of R848 and C6 in the RC6CaL.

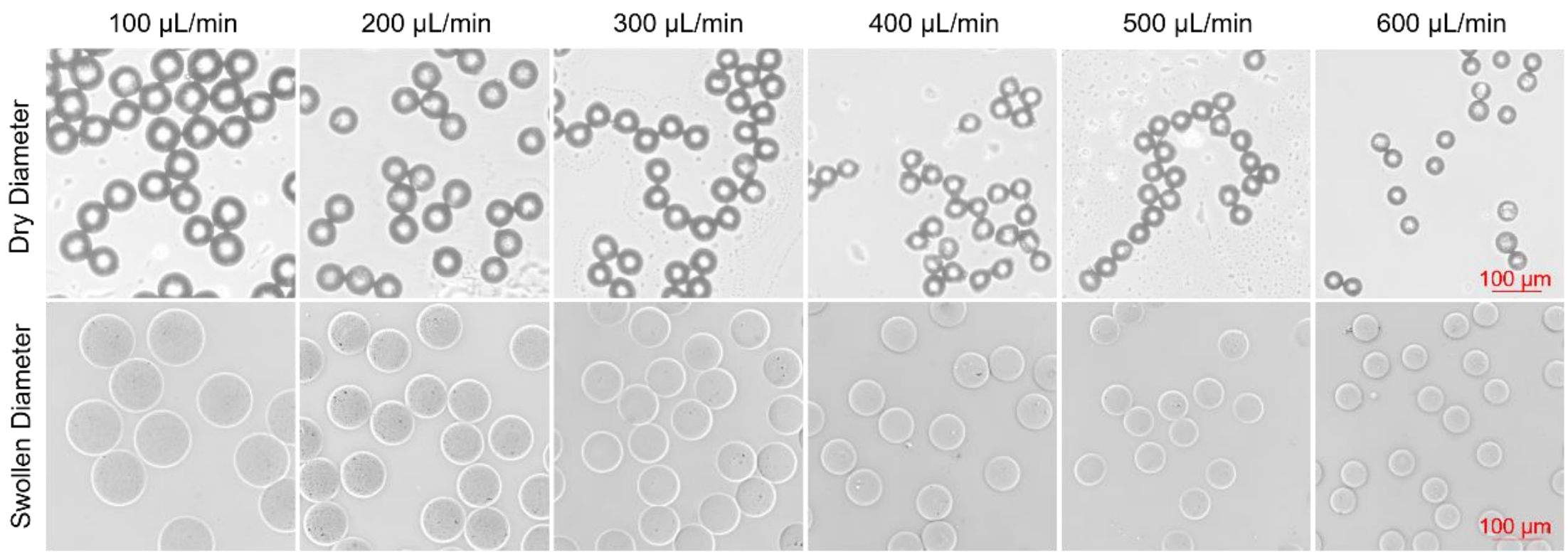

Fig. S9. Effect of the continuous-phase flow rate on the preparation of RC6CaLAlgMS. The optical micrographs of RC6CaLAlgMS prepared using a continuous-phase flow rate ranged from 100 to 600 μL/min. Scale bar: 100 μm.

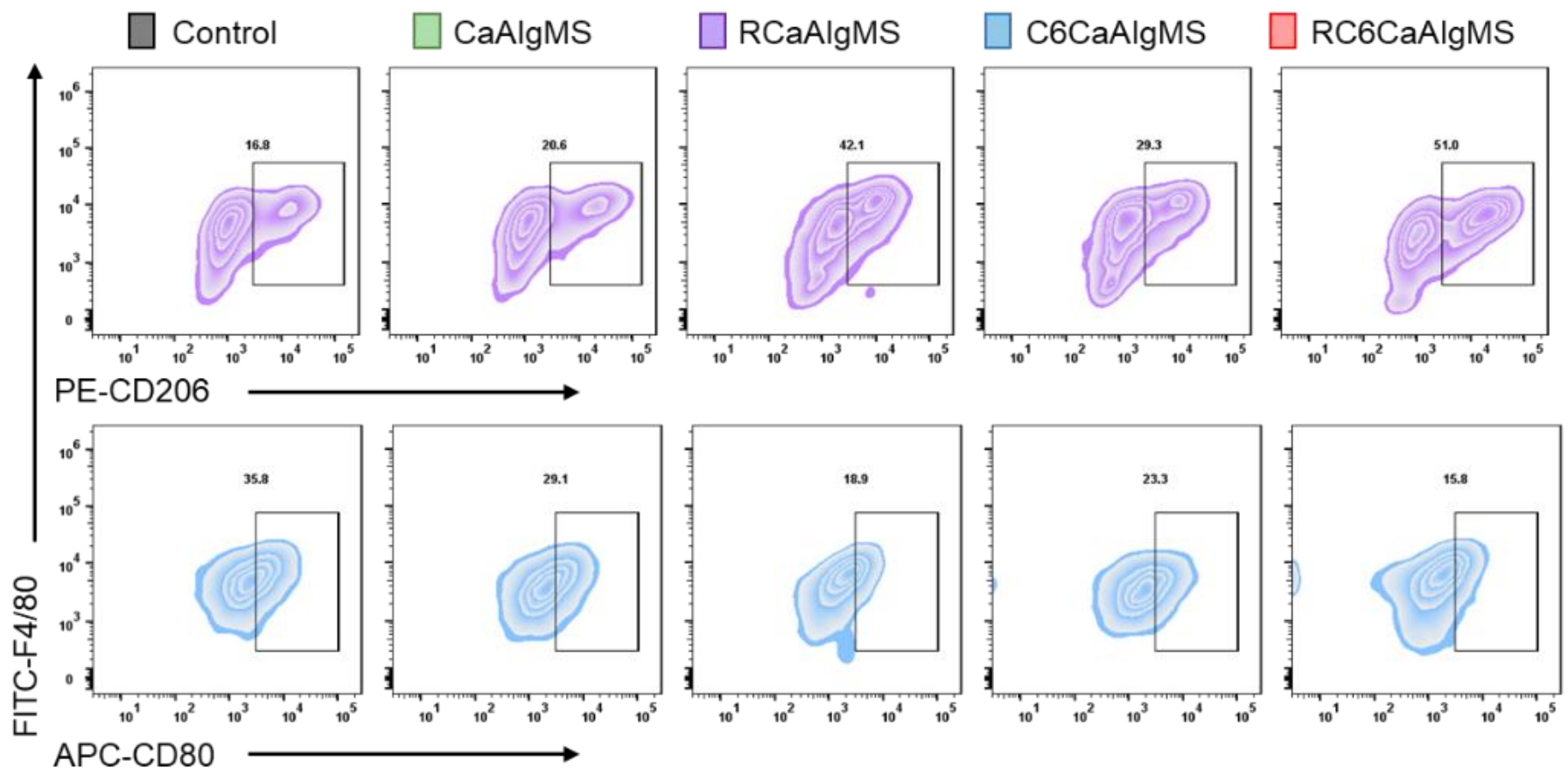

Fig. S10. Representative flow cytometry plots of M2 (F4/80+CD206+) and M1 (F4/80+CD80+) polarization markers in TAMs after various treatments.

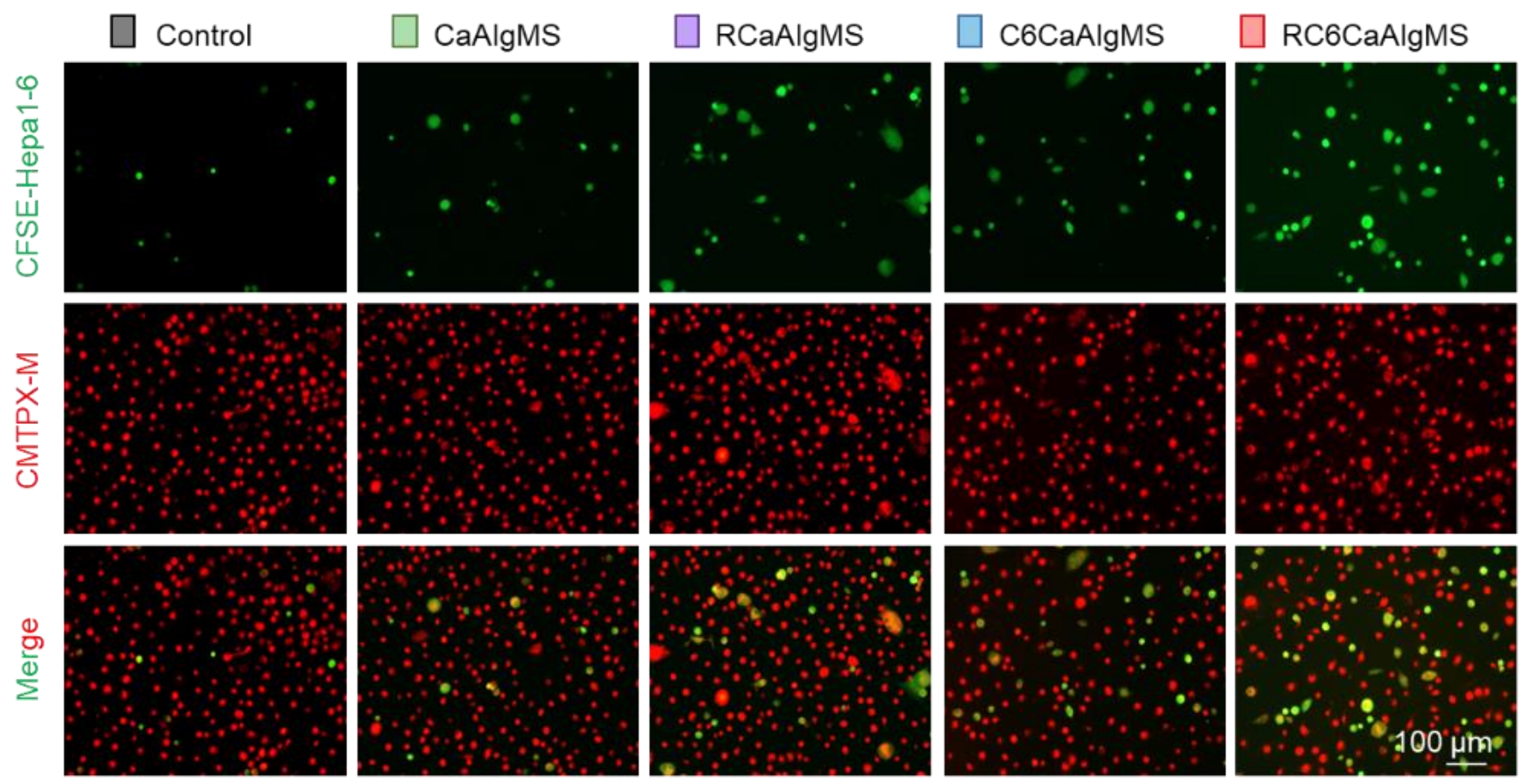

Fig. S11. Representative fluorescence micrographs showing phagocytosis of tumor cells by macrophages across different treatment groups. TAMs were pre-stained with a red Cell Tracker dye (red), and CFSE-labeled tumor cells are shown in green. Yellow arrowheads indicate internalized tumor cells. Scale bar = 100 μm.

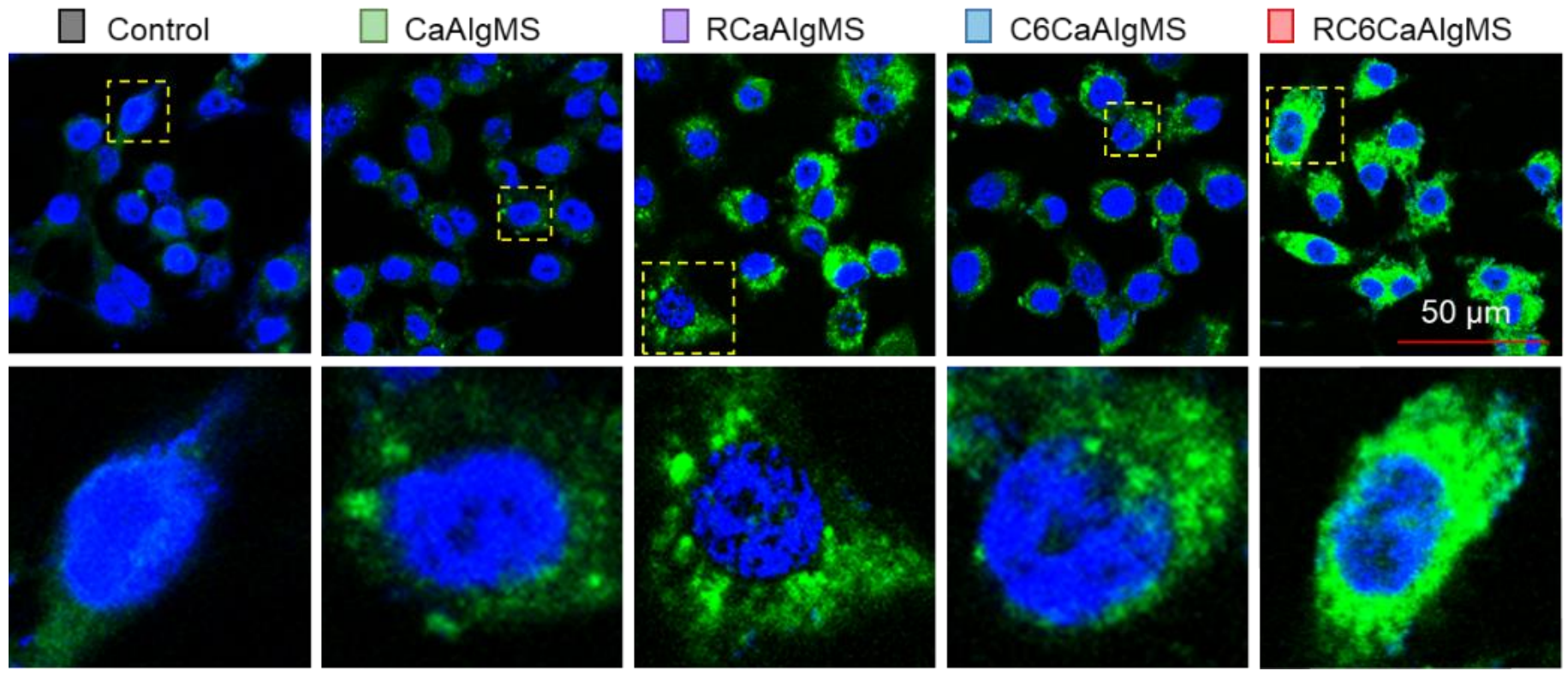

Fig. S12. Representative confocal laser scanning images of phagocytic macrophages. OVA was labeled by FITC (green) and nucleus were stained with DAPI (blue). Scale bar=50 μm.

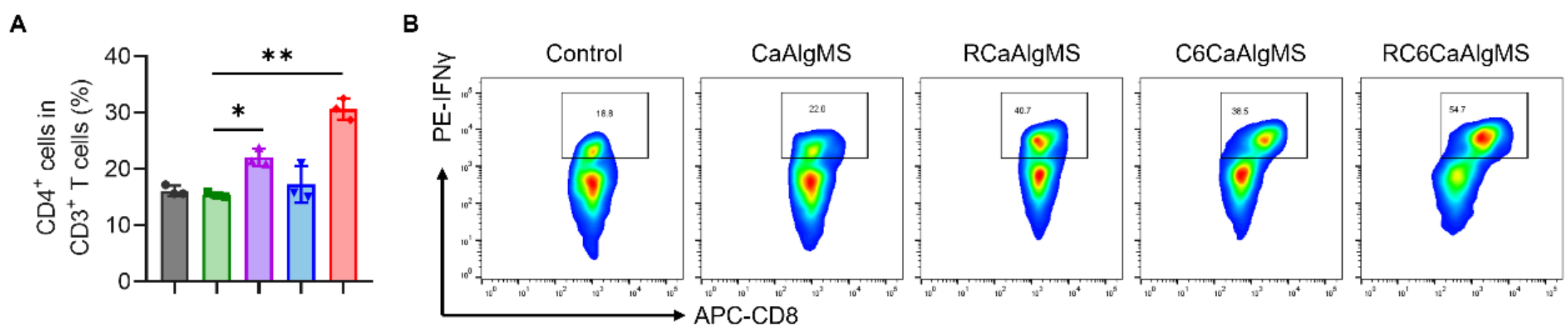

Fig. S13. (A) Semi-quantitative analysis of $CD3^{+}CD4^{+}$ T cell after co-culture with exosomes. (B) Representative flow cytometry plots of $CD8^{+}IFN\gamma^{+}$T cells after co-culture with exosomes. The statistical analysis was performed with ANOVA. P > 0.05 indicated no significance between two groups. *P < 0.05, **P < 0.01.

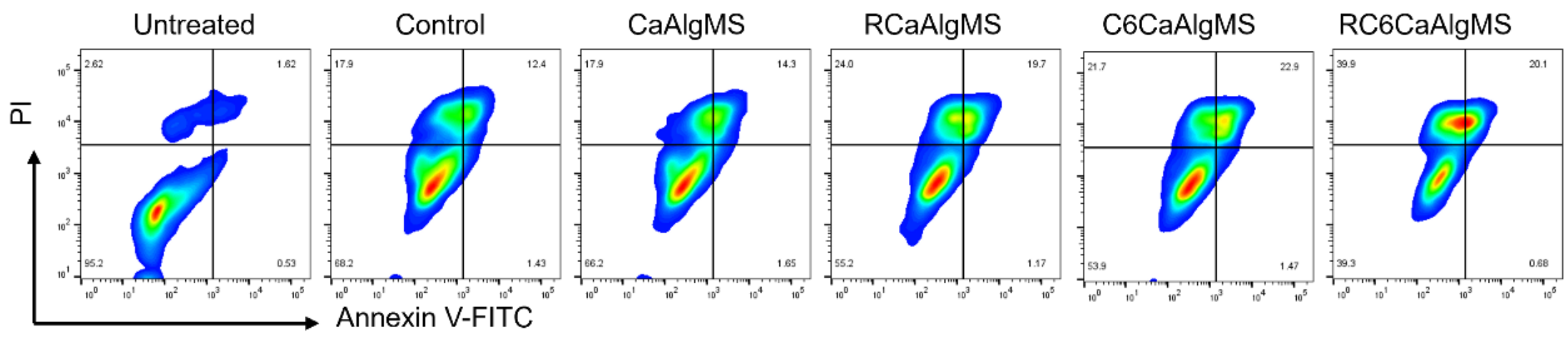

Fig. S14. Representative flow cytometry plots of tumor cell apoptosis when T cells were co-cultured with tumor cells in the presence of antigen and exosomes derived from differently treated macrophages.

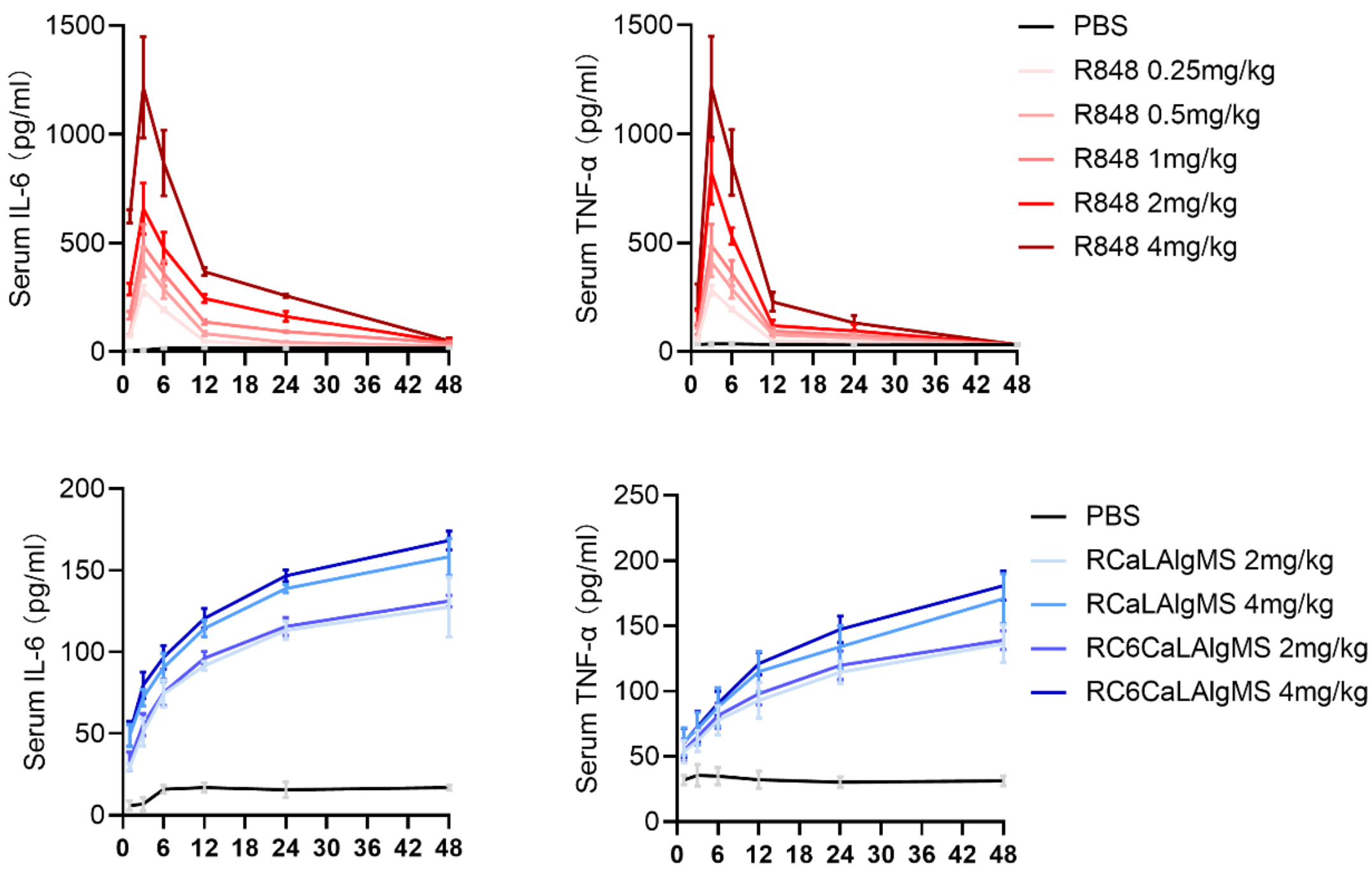

Fig. S15. Serum levels of IL-6 and TNF-α within 48 h after peritumoral injection of R848 and RC6CaLAlgMS in Hepa1-6-tumor-bearing mice.

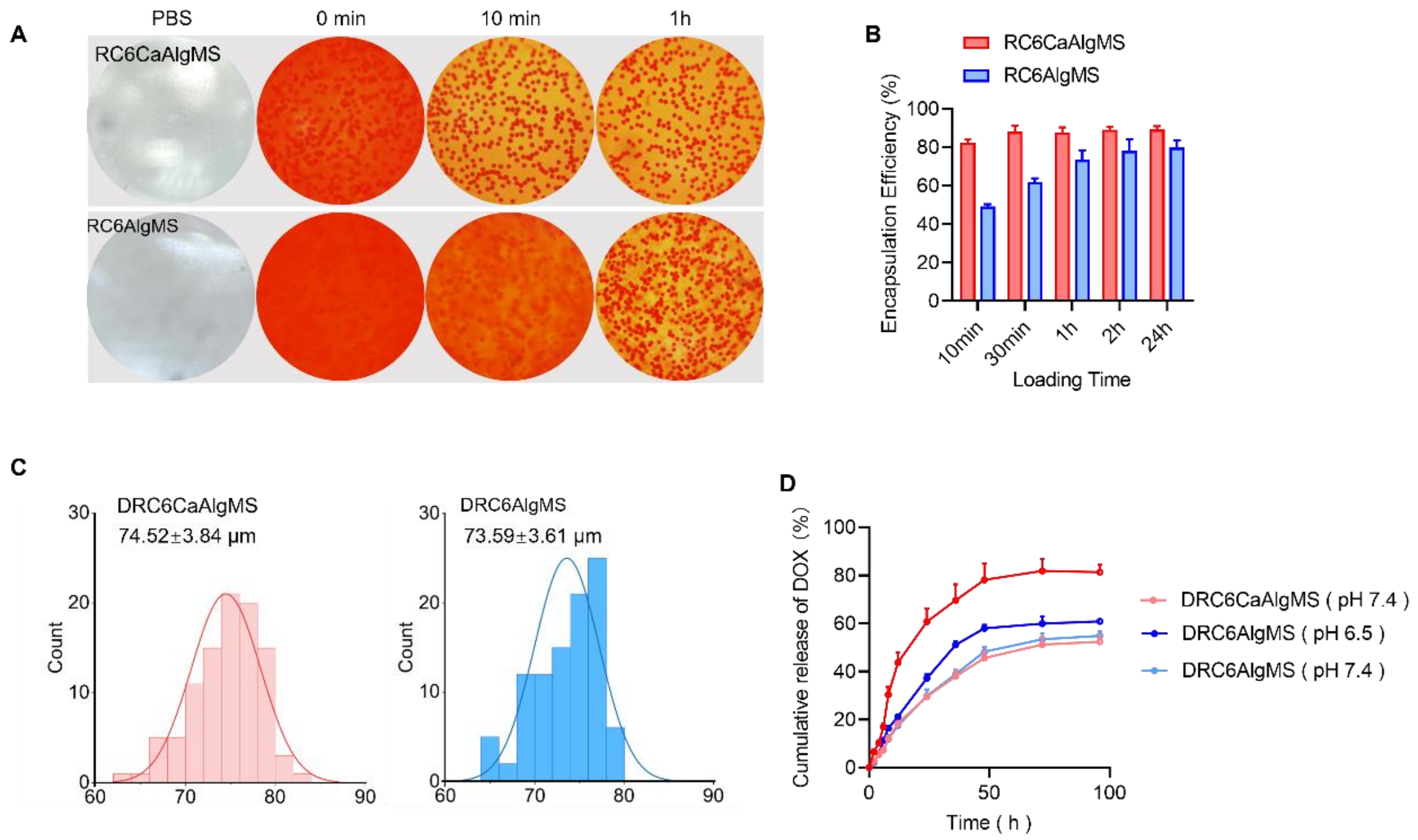

Fig. S16. Characterization of drug loading capacity and physicochemical properties of RC6CaLAlgGMS and RCAlgMS. (A) Doxorubicin (DOX) loading capacity in RC6CaAlgGMS and RCAlgMS microspheres. (B) EE% of DOX in RC6CaLAlgGMS and RCAlgMS microspheres. (C) Size distribution of RC6CaLAlgGMS and DRC6CaLAlgGMS microspheres. (D) The cumulative release of DOX from DRC6CaMs and DRC6Ms was evaluated in PBS at different pH values (6.5 and 7.4) over a period of 96 hours at 37 °C. Data are presented as mean ± SD (n = 3).

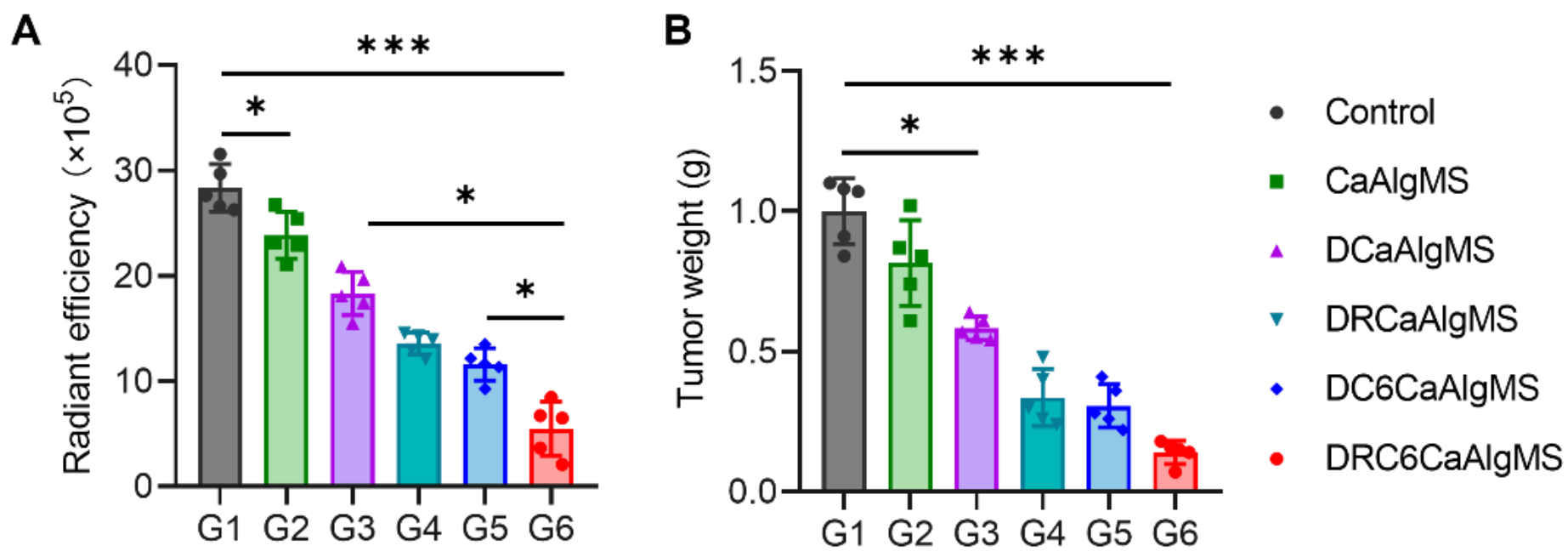

Fig. S17. Antitumor efficacy evaluation in vivo. (A) Quantitative analysis of bioluminescence intensity at the endpoint, revealing the greatest reduction in tumor burden in the DRC6CaAlgMS group. (B) Tumor weights measured at 15 day. The statistical analysis was performed with ANOVA. P > 0.05 indicated no significance between two groups. *P < 0.05, **P < 0.01, ***P < 0.001.

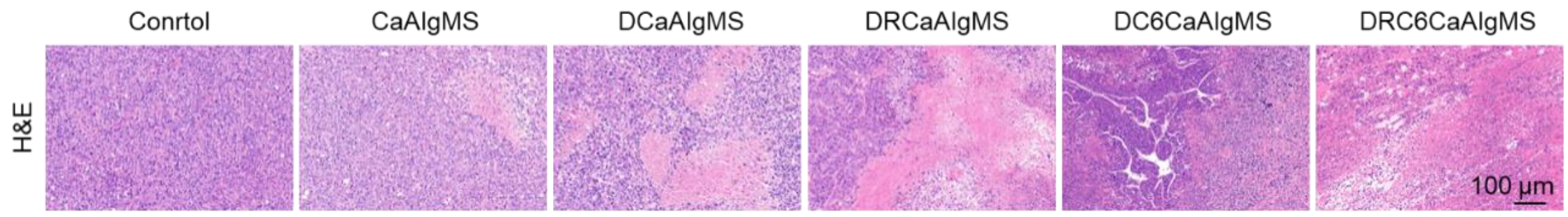

Fig. S18. H&E-stained tumor sections from various treatment groups, assessing overall morphology and necrotic areas. Scale bar, 100 μm.

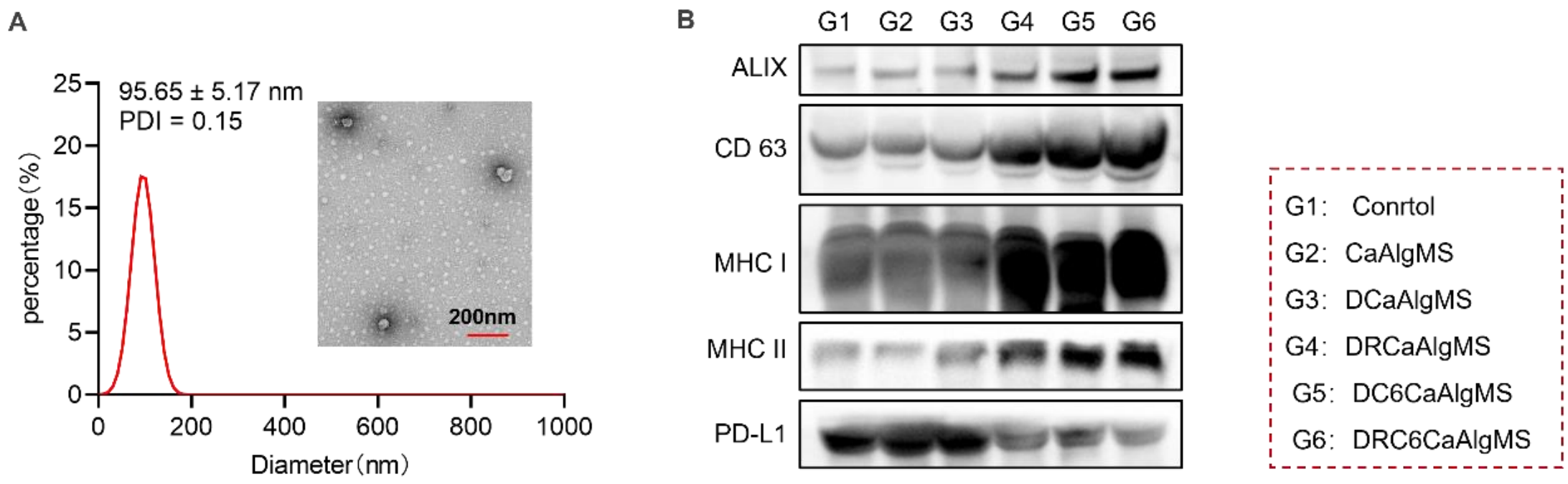

Fig. S19. (A) Characterization of exosomes isolated from plasma. Representative TEM image of the isolated exosomes, showing the classic cup-shaped morphology. Scale bar: 200 nm. Size distribution and concentration of the exosomes as determined by DLS. (B) Western blot analysis of CD45, ALIX, CD63, MHC I, MHC II and PD-L1 in plasma-derived exosomes.

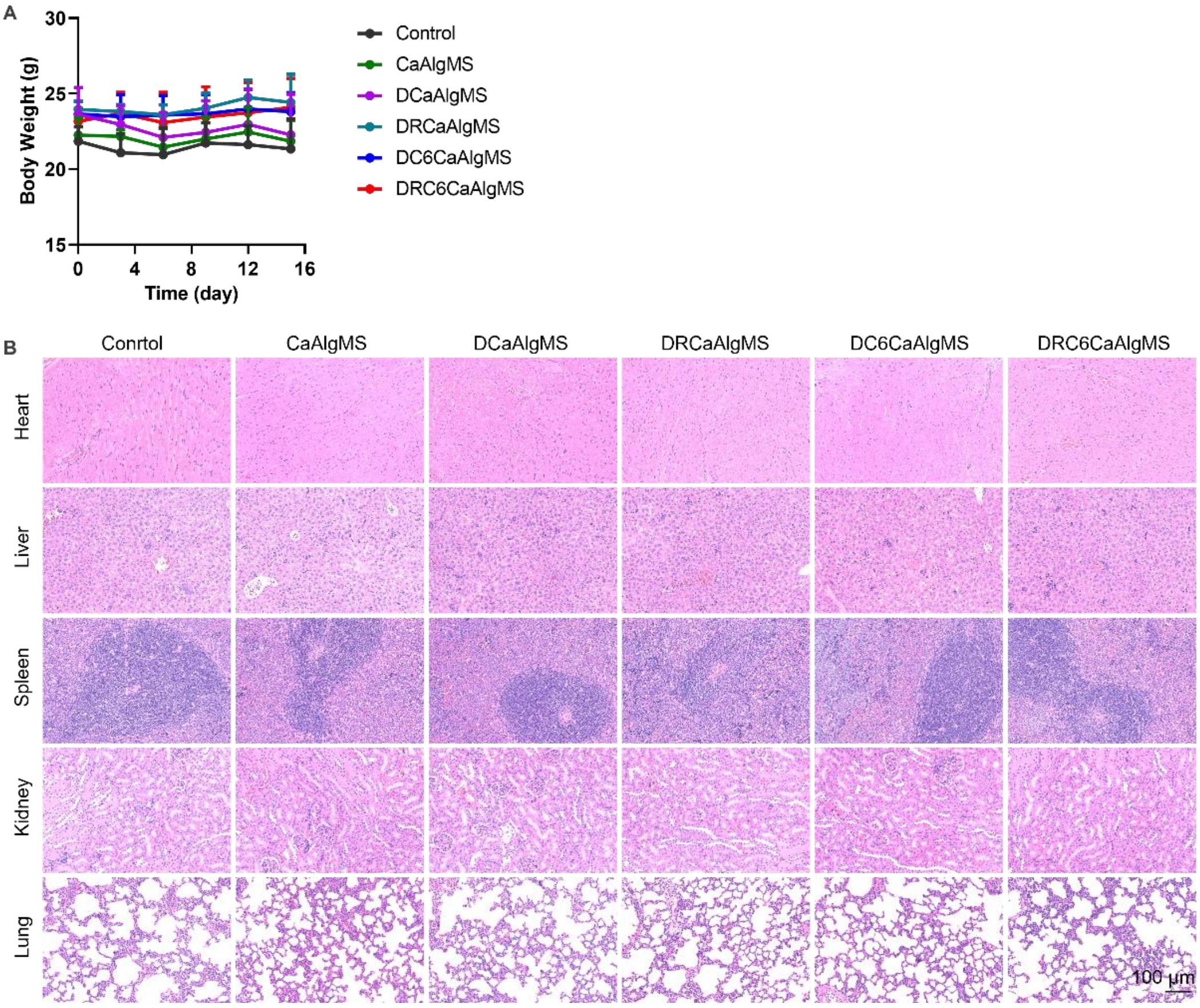

Fig. S20. Systemic safety evaluation. (A) Body weight monitoring throughout treatments. (B) Representative H&E-stained images of major organs (heart, liver, spleen, lungs, and kidneys) collected from mice in different treatment groups 15 day. No significant histopathological abnormalities or lesions were observed in any of the groups, indicating the favorable biosafety profile of the treatments. Scale bar:100 μm.

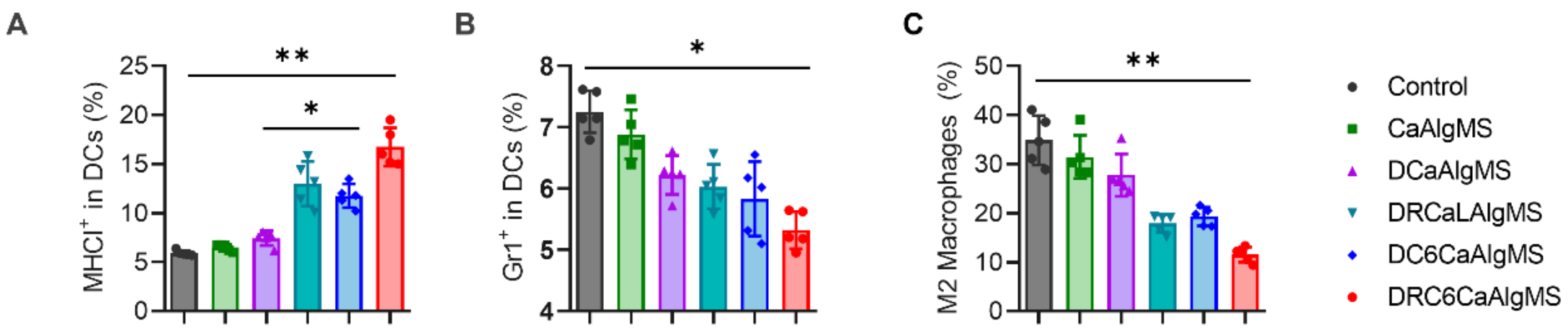

Fig. S21. Semi-quantitative analysis of MHCI^{+} DCs (A) Gr1^{+} DCs (B) in TDLNs and M2 macrophages in tumors (C) The statistical analysis was performed with ANOVA. P > 0.05 indicated no significance between two groups. *P < 0.05, **P < 0.01.

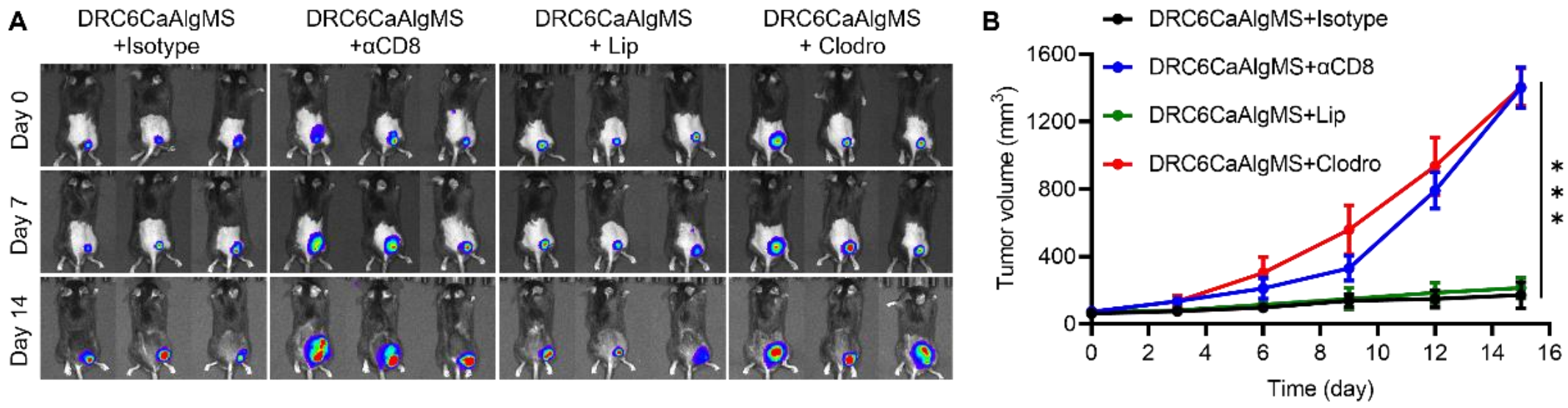

Fig. S22. (A) In vivo bioluminescence images of representative mice from different combination therapy groups at 0,7 and14 day post-treatment. Mice were treated with RC6CaLAlgMS in combination with Isotype, αCD8, lipsome and clodro, respectively. (B) Tumor growth curves of the different treatment groups over a 15-day period. The statistical analysis was performed with ANOVA. P > 0.05 indicated no significance between two groups. *P < 0.05, **P < 0.01, ***P < 0.001.

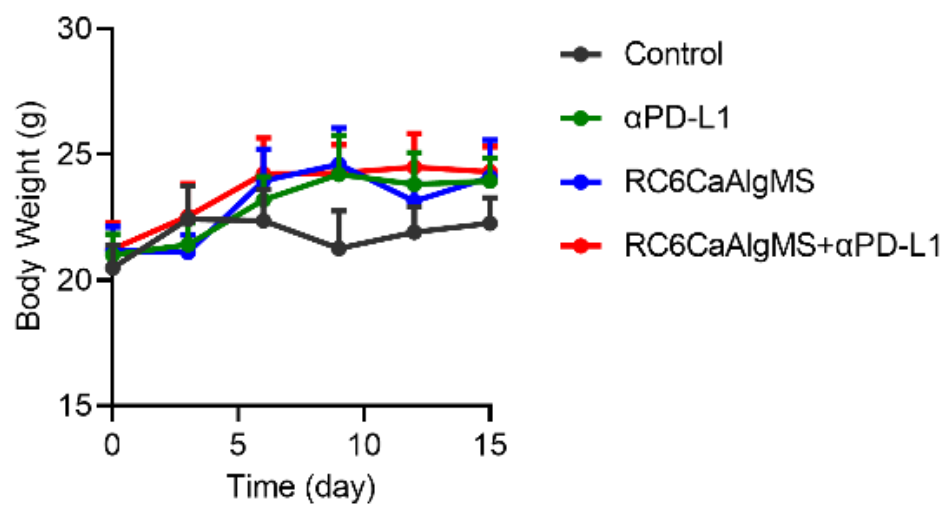

Fig. S23. Body weight monitoring throughout treatment. Data are presented as mean ± SD (n = 5 per group).

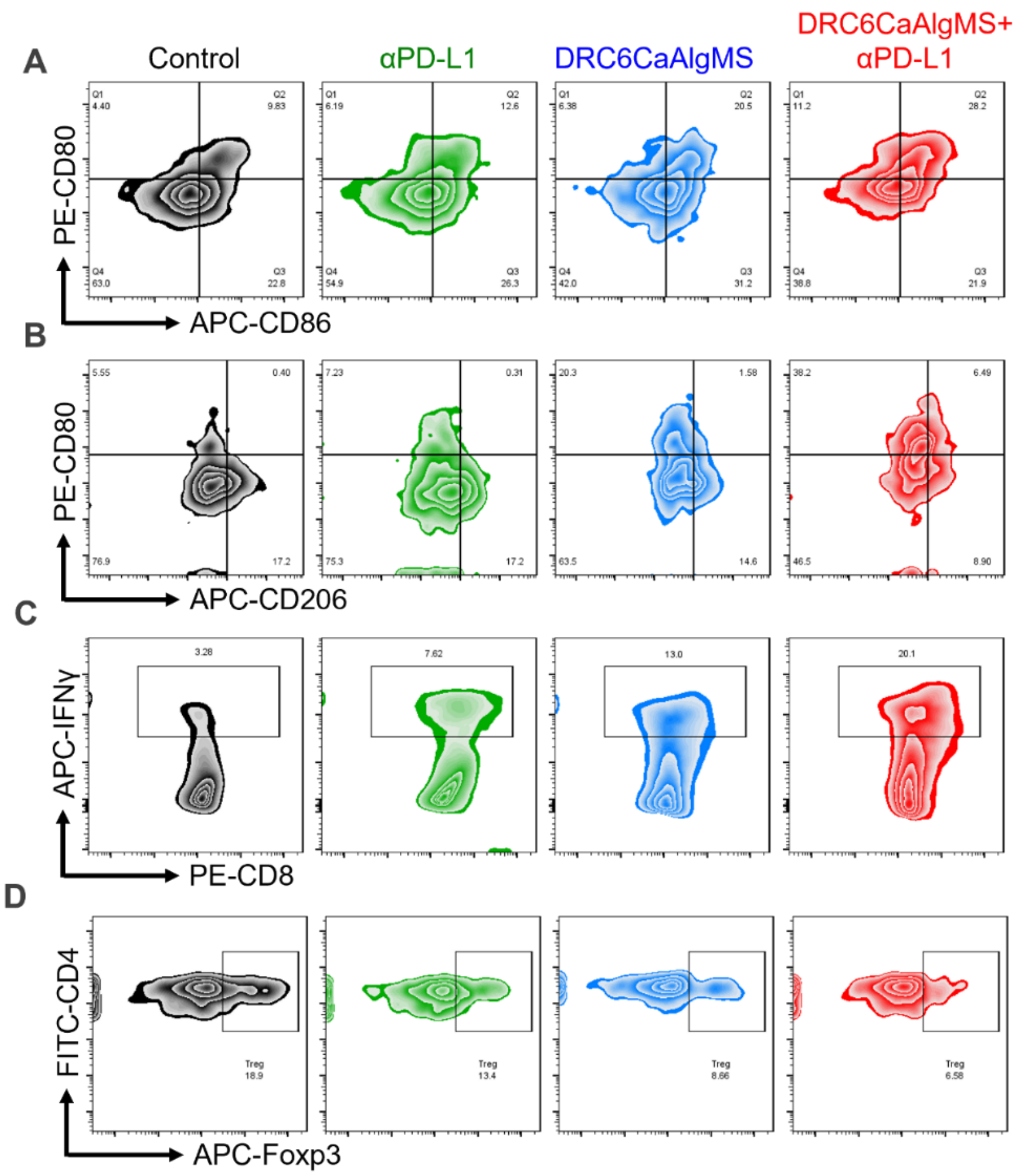

Fig. S24. Representative flow cytometry plots of immune cell infiltration in TDLNs and tumor tissues after different treatments. (A)Representative flow cytometry plots of mature dendritic cells ($CD80^{+}CD86^{+}$DCs) in tumor-draining lymph nodes. Representative flow cytometry plots of tumor-infiltrating immune cell subsets, including (B) M1 macrophages, M2 macrophages, (C) $CD3^{+}CD8^{+}IFN\gamma^{+}$Tcells and (D)Treg cells.

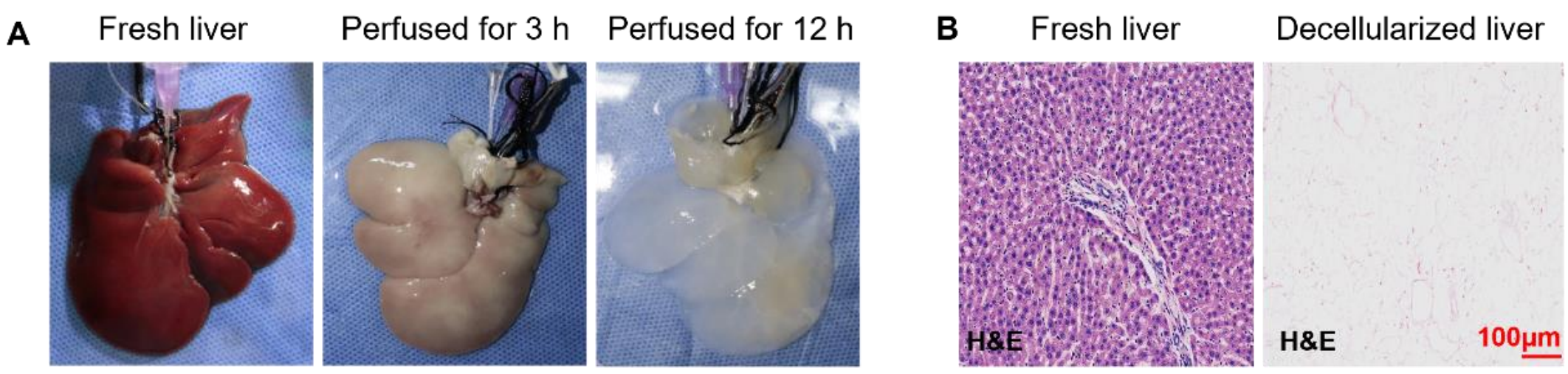

Fig. S25. Process and validation of liver decellularization. (A) (Left) Fresh liver after thawing at -80°C. (Middle) Liver treated with 4% Triton X-100/0.02% EGTA solution

for 3 hours. (Right) Liver further treated with 0.5% sodium dodecyl sulfate (SDS) solution for 3 hours. (B) H&E staining of fresh liver and decellularization liver.

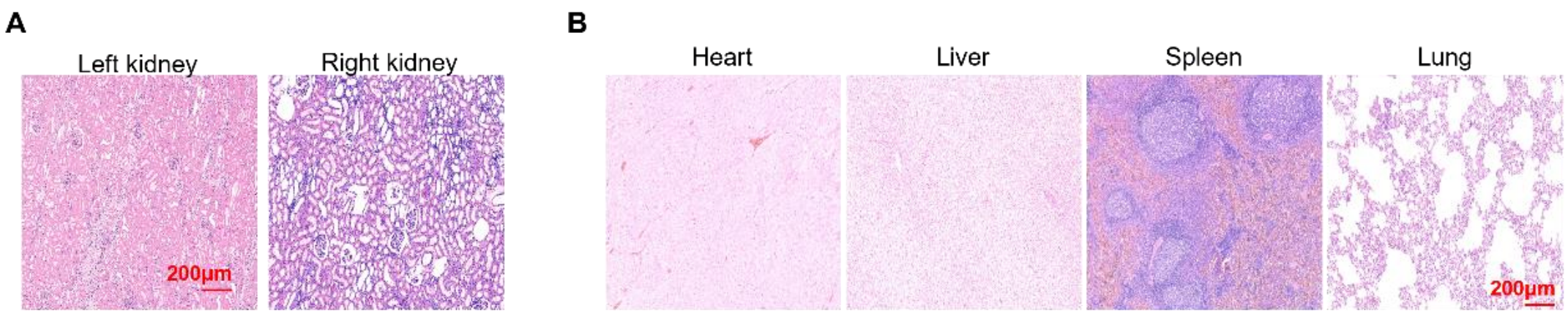

Fig. S26. Evaluation of embolization efficacy and safety in a rabbit renal embolism model. (A) Histological analysis of embolized and non-embolized kidneys by H&E staining. The embolized kidney shows extensive coagulative necrosis and loss of cellular architecture, while the non-embolized kidney maintains normal renal histology. Scale bar: 200 μm.(D) Safety evaluation by H&E staining of major organs (heart, liver, spleen, and lung) after embolization. No significant pathological abnormalities or embolic complications were observed in these organs, demonstrating the localized effect and good safety profile of the embolization procedure. Scale bar: 200 μm.

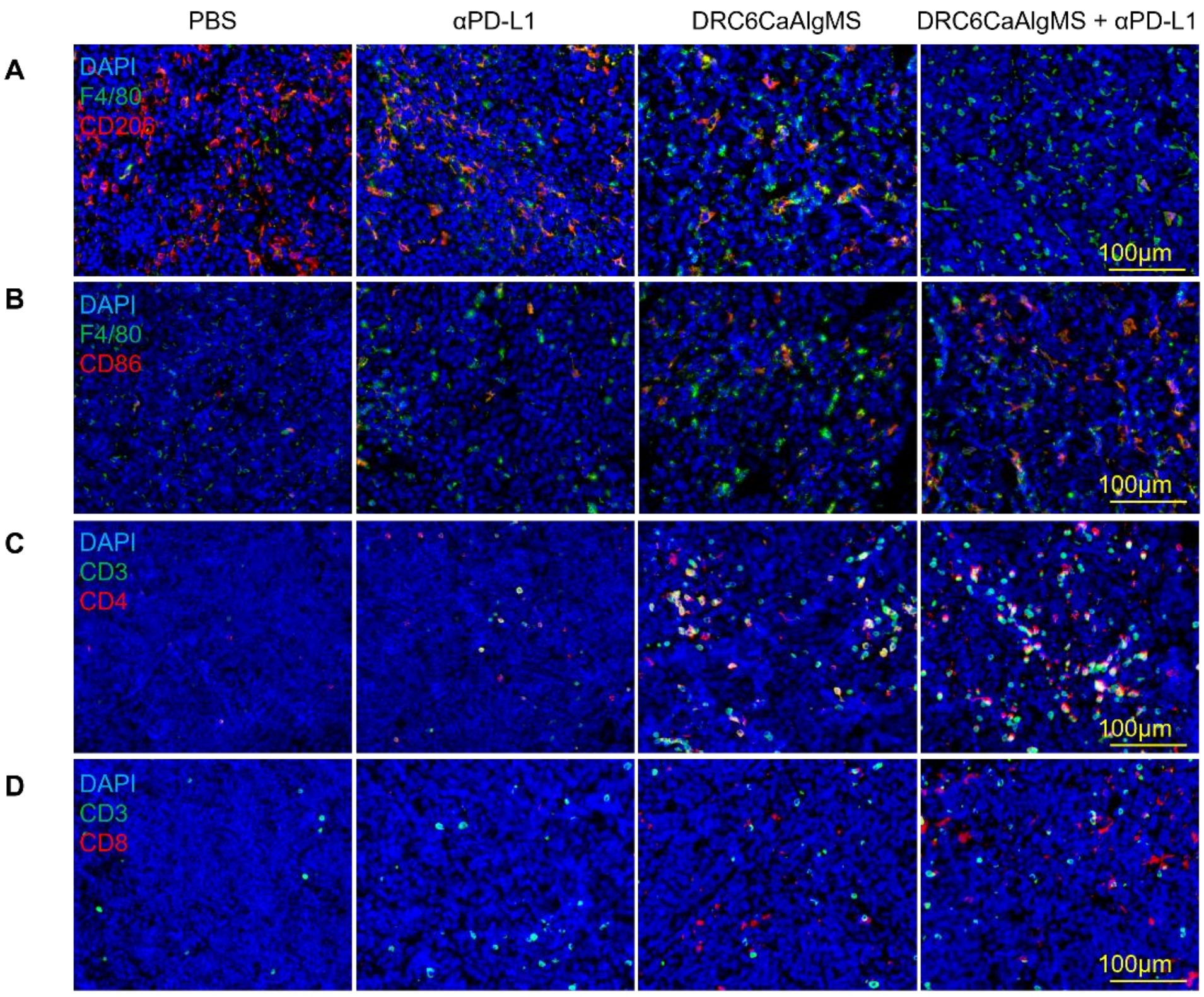

Fig. S27. Confocal microscopic observations F4/80$^+$CD206$^+$cells (A), F4/80$^+$CD86$^+$cells (B), CD3$^+$CD4$^+$ cells (C) and CD3$^+$CD8$^+$ cells (D) infiltration within rat tumors after various treatments.

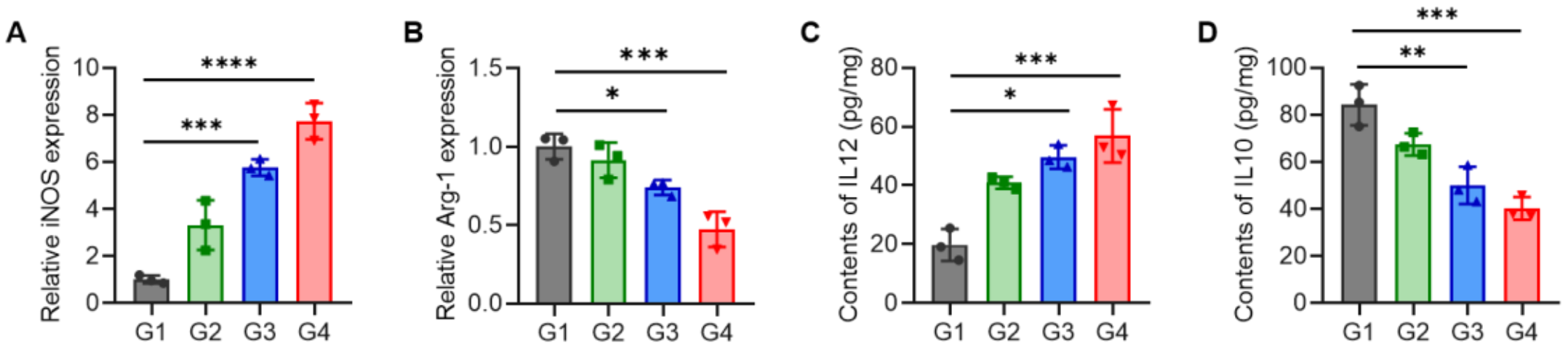

Fig. S28. (A) Represents M1 marker genes, and (B) represents M2 marker genes. Represents M1 marker proteins, and (b) represents M2marker proteins. G1: Control, G2: αPD-L1, G3: DRC6CaAlgMS, G4: DRC6CaAlgMS + αPD-L1. *P < 0.05, **P < 0.01, ***P < 0.001, ****P < 0.0001.

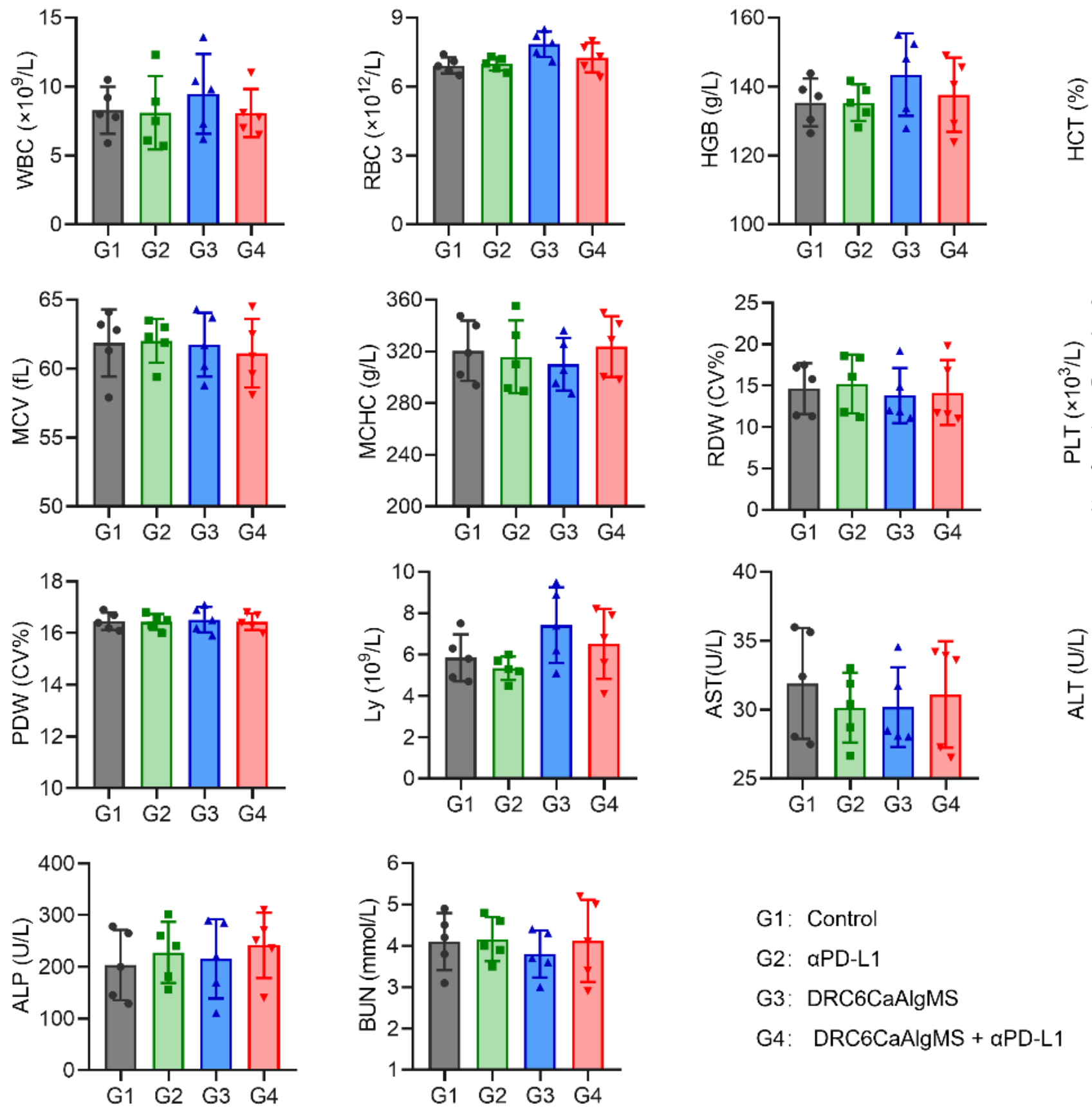

Fig. S29. The results of blood biochemistry tests of these rats after various treatment. The results of White blood cell count (WBC, 5.0-15.0 $\times 10^9$/L), Red blood cell count (RBC, 6.0-9.0 $\times 10^{12}$/L), Hemoglobin (HGB, 120-160 g/L), Hematocrit (HCT, 35-50 %), Mean corpuscular volume (MCV, 50-70 fL), Mean corpuscular hemoglobin concentration (MCHC, 280-360 g/L), Red cell distribution width (RDW, 10-20 CV%), Platelet count (PLT, 400-1000 $\times 10^3$/L), Platelet distribution width (PDW, 8-15 CV%),

Absolute lymphocyte count (Ly, 2.0-10.0 $\times 10^9$/L), Aspartate aminotransferase (AST, 21.53-61.75 U/L) Alanine aminotransferase (ALT, 30-100 U/L), Alkaline phosphatase (ALP, 12.04-610.97 U/L), Blood urea nitrogen (BUN, 3.0-7.0 mmol/L)
